# Unexpected Domino Silyl-Prins/Aryl Migration Process from Geminal Vinylsilyl Alcohols

**DOI:** 10.1021/acs.orglett.1c03121

**Published:** 2021-10-07

**Authors:** Carlos Díez-Poza, Asunción Barbero

**Affiliations:** Department of Organic Chemistry, Faculty of Sciences, University of Valladolid, Campus Miguel Delibes, Paseo de Belén 7, 47011 Valladolid, Spain

## Abstract

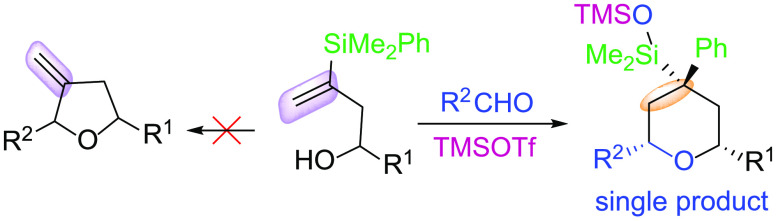

The silyl-Prins cyclization of geminal vinylsilyl alcohols and aldehydes, promoted by TMSOTf, provides access to polysubstituted tetrahydropyrans in which the silyl group remains in the molecule and an aryl group has migrated from silicon to carbon. This domino silyl-Prins/aryl migration process is general and high-yielding for aryl, vinyl, or alkyl aldehydes. Moreover, cyclization proceeds with very high stereocontrol in a one-pot reaction in which both quaternary and tertiary stereogenic centers have been created.

The range of structurally diverse oxygen-containing heterocyclic natural products is enormous. Within them, polysubstituted tetrahydropyrans represent one of the most common structural features in biologically active heterocycles.

Numerous methodologies have been developed to synthesize these types of structures. Within them, Prins cyclization has proven to be an efficient and reliable protocol to build tetrahydropyrans in a very stereoselective manner.^1^ The process typically involves the reaction of homoallylic alcohols with aldehydes in the presence of an acid (either a protonic or a Lewis acid) to provide intermediate tetrahydropyranyl cations, which are then trapped by nucleophiles. An interesting modification of the Prins cyclization, which involves the participation of an electron-rich silylated alkene, is the so-called silyl-Prins cyclization.

Within the silylated alkenes used in silyl-Prins cyclizations, allylsilanes have frequently shown a great potential for the stereoselective synthesis of different-sized oxygen and nitrogen heterocycles.^2^ The alternative use of vinylsilanes in this process has been much less developed, although it has effectively been applied to the synthesis of dihydropyrans,^3,4^ starting from (*Z*)-vinylsilyl homoallylic alcohols. However, a rather limited number of examples has been reported for the synthesis of alkylidene oxacycles from vinylsilyl homoallylic alcohols in which the silyl group and the alcohol (or the corresponding oxocarbenium ion precursor) are bonded to the same sp^2^ carbon. The main feature of both types of vinylsilyl-mediated Prins cyclizations is the stabilization of the intermediate carbocation β to silicon and the subsequent elimination of the silyl group, to form an endocyclic or exocyclic double bond (respectively) (Scheme 1a,b).

**Scheme 1:**
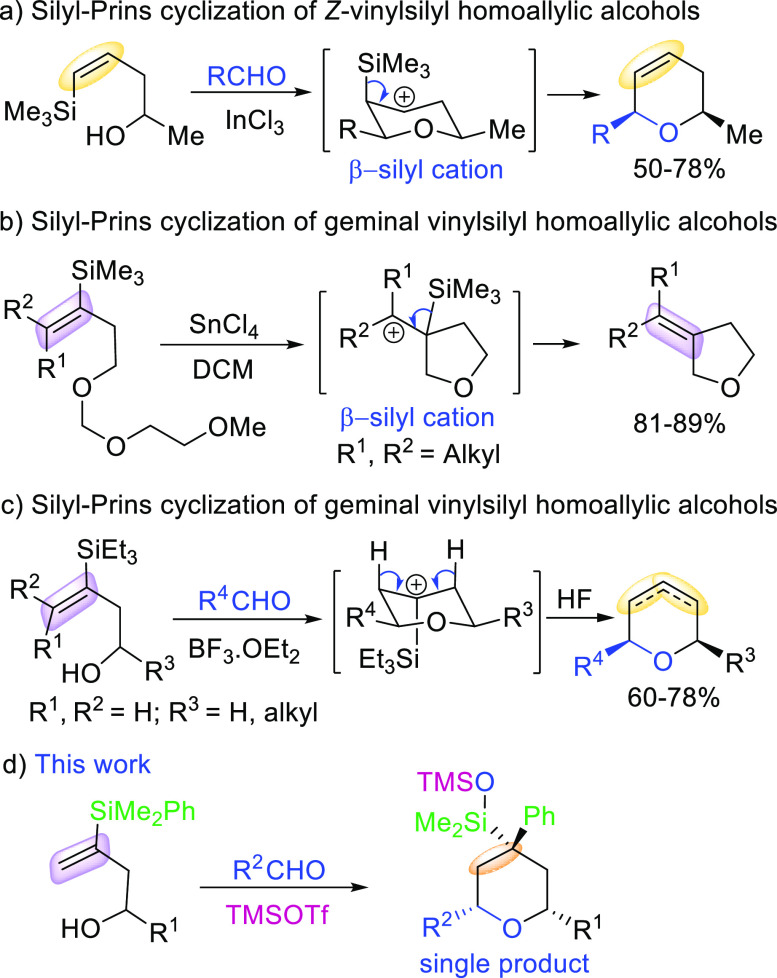
Silyl-Prins Cyclization of Vinylsilyl Homoallylic Alcohols

Interestingly, Saikia has recently reported an example in which the silyl-Prins cyclization of a similar geminal vinylsilyl homoallylic alcohol (with both R^1^ and R^2^ being hydrogens) follows a different pathway, now leading to dihydropyran derivatives.^5^ Prins cyclization would provide a carbocation α to silicon, which then could eliminate an adjacent proton to give a silylated dihydropyran. Final desilylation by *in situ* generated HF acid would explain the formation of the final product (Scheme 1c).^6^

In our continuing effort to develop new synthetic methodologies for the preparation of different-sized carbo-^7^ and heterocycles^8^ from organosilanes, we have recently found that allylsilyl alcohols^9^ and amines^10^ undergo a silyl-Prins cyclization to provide seven- or eight-membered heterocycles in a very effective manner. In this paper we report an unprecedented cyclization of geminal vinylsilyl alcohols leading to 4-silyl-4-aryltetrahydropyrans through a domino Prins/aryl migration process.

We discovered this new multicomponent reaction during the screening of the optimized conditions for the silyl-Prins cyclization of vinylsilyl alcohol **1a** with cinnamaldehyde, in the presence of a variety of acids (both protonic and Lewis acids). The results are illustrated in Table 1.

**Table 1:**
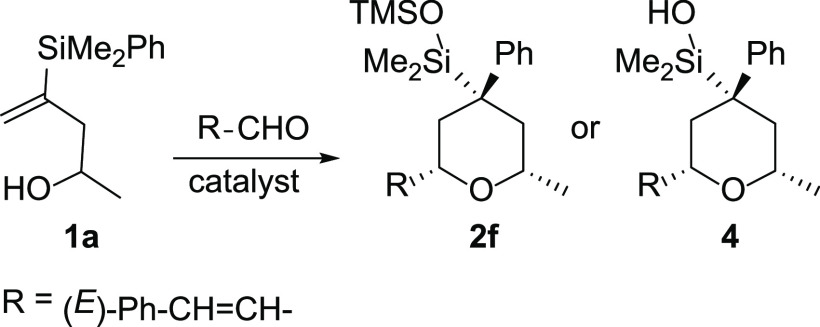
Optimization of the Silyl-Prins Cyclization of Vinylsilyl Alcohol **1a** with Cinnamaldehyde

entry	acid	conditions^a^	product (yield, %)
1	Bi(OTf)_3_	rt, 30 min	CM^b^
2	Bi(OTf)_3_	–78 to 0 °C, 3 h	CM^b^
3	Sc(OTf)_3_	rt, 2 days	CM^b^
4	pTSA	rt, 6 days	CM^b^
5	TFA	rt, 7 h	CM^b^
6	TfOH	–80 °C, 1 h	**4** (20)^c^
7	TfOH	–80 °C, 1 h	**4** (35)
8	TMSOTf	–78 °C, 2 h	**2f** (15)^c^
9	TMSOTf	–78 °C, 30 min	**2f** (70)

a Unless otherwise indicated, all of the reactions were carried out in CH_2_Cl_2_ using 1 equiv of the acid.

b CM stands for complex mixture.

c 0.5 equiv of the acid was used.

As shown in Table 1, a complex reaction mixture is obtained in the presence of Bi(OTf)_3_, Sc(OTf)_3_, pTSA, or TFA (Table 1, entries 1–5). Surprisingly, the reaction under TMSOTf activation provided the 4,4-disubstituted tetrahydropyranyl derivative **2f** in which the silyl group remains in the ring and the phenyl moiety has migrated from silicon to the adjacent carbon (Table 1, entry 9). The use of 1 equiv of TMSOTf is necessary to achieve full conversion of the starting alcohol (Table 1, entry 8). The analogous product **4** is obtained when the acid used is TfOH, although in this case the reaction proceeds in lower yields (Table 1, entries 6 and 7). In both cases, a single stereoisomer is obtained with total stereocontrol.

Next, we examined the applicability of this multicomponent process to the synthesis of different 2,4,4,6-tetrasusbstituted tetrahydropyrans, exploring the reaction of a variety of aldehydes with alkylic alcohols **1a**–**c**. The results are shown in Scheme 2.

**Scheme 2:**
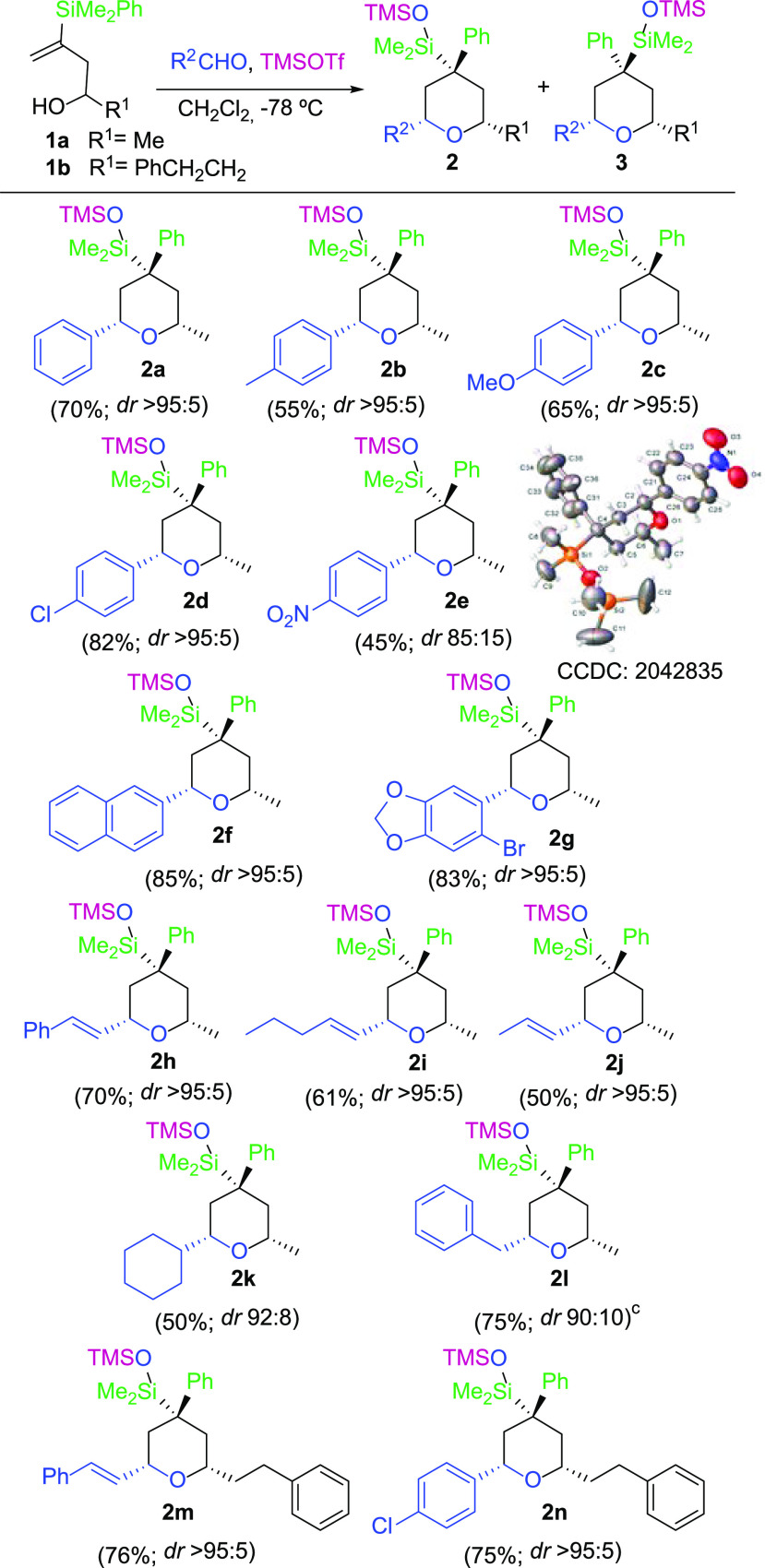
Scope of the Domino Silyl-Prins Migration Process Using Vinylsilyl Alcohols

As depicted in Scheme 2, aryl aldehydes bearing both electron-donating and electron-withdrawing groups give satisfactory yields of the corresponding polysubstituted tetrahydropyrans. In addition, vinyl and aliphatic aldehydes also furnish the desired products in good yields.

Moreover, excellent stereoselectivities are observed for most products, in a multicomponent reaction in which both tertiary and quaternary stereocenters are created in one pot.

The structure and stereochemical assignment of tetrahydropyrans **2** was unambiguously determined by NMR techniques as well as by an X-ray diffraction analysis of compound **2e**(11) (Scheme 2).

A plausible mechanism for this multicomponent reaction is shown in Scheme 3. The reaction, most likely, starts with the condensation of the alcohol and the aldehyde to give the oxocarbenium ion **I**, which readily undergoes attack by the vinylsilane moiety. The subsequent formation of the tetrahydropyranyl tertiary carbocation **II** α to silicon (more stable than the primary β-silyl cation)^12^ will be followed by a Friedel–Crafts reaction of the phenyl group attached to silicon onto the adjacent cation, to form a stabilized cation β to silicon. Further attack of the alkoxide onto the silicon would induce the migration process.

**Scheme 3:**
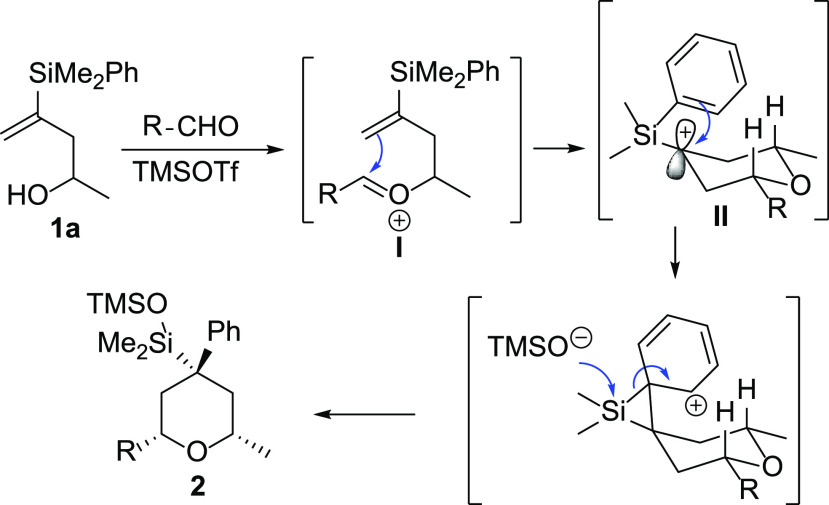
Mechanism of the Domino Silyl-Prins Migration Process

No methyl migration was observed in any case, which seems to indicate that the driving force for this process is the ability of the phenyl migrating group to stabilize the positive charge at the adjacent carbocation.^13^

It has to be noted that group migrations from silicon to carbon are rather uncommon processes.^14^ In fact, apart from the rearrangement of α-chloroalkylsilanes in the presence of acids,^15^ the number of processes that evolve with alkyl migration from silicon to the contiguous carbon are very scarce and, as far as we know, have never been reported for silyl-Prins cyclizations.

Thus, we now describe an unprecedented domino multicomponent silyl-Prins cyclization in which a quaternary center is formed by 1,2-Si to C migration. Furthermore, this novel process is general and proceeds with satisfactory yields in every case studied (Scheme 2).

However, the most fascinating feature of this migration process, no doubt, is the highly stereoselective manner in which it occurs, since an almost unique diastereoisomer is obtained in most cases. The formation of the 2,6-*cis* tetrahydropyran is easily rationalized through a chairlike transition state in which both substituents adopt the most stable equatorial conformation. More striking is the stereoselective formation of the quaternary center at C-4. Our hypothesis is that the observed axial migration^16^ from silicon to the adjacent carbocation can arise from a preferred chairlike conformation in which there is an efficient hyperconjugative overlap between the antibonding σ* orbital of the forming C–C bond and the vicinal C–H σ donor bond. This electron-donating stabilization effect would favor the axial attack, overriding the steric preference for the equatorial approach (Figure 1). To the best of our knowledge, this is the first 1,2-Si to C migration in which a quaternary stereogenic carbon is created with total stereocontrol. Thus, the simultaneous construction of quaternary and tertiary stereogenic carbons with total stereocontrol is one of the crucial aspects of this multicomponent process.

**Figure 1:**
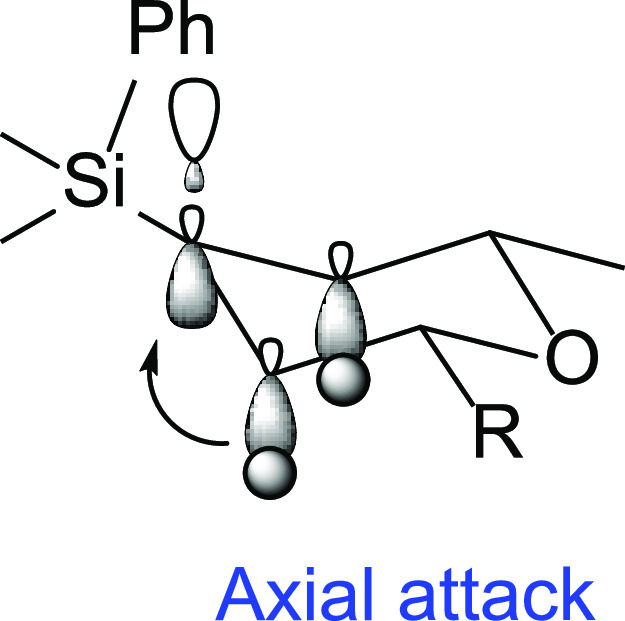
Stereoselectivity of the process.

We then decided to study the influence of the vinylsilyl alcohol substituents on the outcome of the process. For that purpose, we chose a homoallylic alcohol with an aromatic ring (phenyl) in the side chain (alcohol **1c**). The results are shown in Table 2.

**Table 2:**
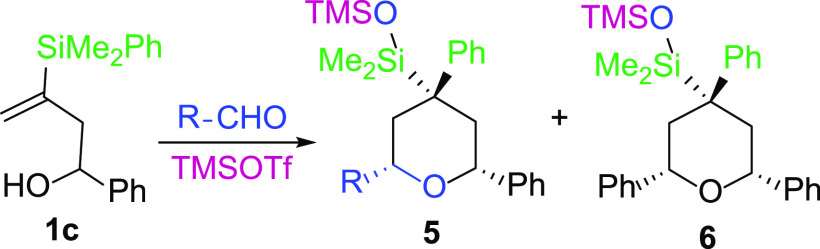
Scope of the Process Using Benzylic Alcohol **1c**

entry	R-CHO	amt of RCHO (equiv)	conditions	ratio **5**:**6**^b^	product (yield, %)
1	PhCH_2_	1.3	–78 °C, 0.1 M	93:7	**5a** + **6** (77)^c^
2	PhCH_2_	2^a^	–78 °C, 0.1 M	94:6	**5a** + **6** (30)^c^
3	PhCH_2_CH_2_	1.3	–95 °C, 0.05 M	93:7	**5b** + **6** (55)^c^
4	4-MeOPh	1.1	–78 °C, 0.1 M	50:50	**5c** + **6** (50)^d^
5	4-MeOPh	1.1	–95 °C, 0.1 M	50:50	**5c** + **6** (45)^d^
6	4-MeOPh	1.1^a^	–78 °C, 0.05 M	60:40	**5c** + **6** (55)^d^
7	4-MeOPh	2^a^	–78 °C, 0.05 M	85:15	**5c** + **6** (60)^d^
8	4-MeOPh	3^a^	–95 °C, 0.05 M		CM
9	4-ClPh	2^a^	–95 °C, 0.05 M		**5d** + **6** (70)^c^^,^^e^^,^^f^
10	(*E*)-PhCH=CH	1.1	–95 °C, 0.05 M	25:75	**5e** + **6** (40)^c^^,^^d^
11	(*E*)-PhCH=CH	2^a^	–78 °C, 0.1 M	75:25	**5e** + **6** (45)^c^^,^^d^

a Inverse addition: the alcohol is added to a solution of the aldehyde and catalyst in DCM.

b The ratio of products was determined by a ^1^H NMR (400 MHz) analysis of the crude mixture.

c The Prins and the Cope products could not be separated.

d Small amounts of benzaldehyde were observed in the reaction mixture.

e Conversion.

f The coalescence of signals in the H NMR spectrum of the Prins and the Cope products made it impossible to measure the ratio.

As shown (Table 2, entry 4), the reaction of alcohol **1c** with anisaldehyde mediated by TMSOTf, under the standard conditions, afforded an equimolar mixture of the expected tetrahydropyran **5c** and another compound **6** bearing a plane of symmetry, which was shown to be the corresponding product of an oxonia-Cope rearrangement. An oxonia-Cope rearrangement is known to be a competitive reaction in Prins cyclizations,^17^ whose mechanism is shown in Scheme 4.

**Scheme 4:**
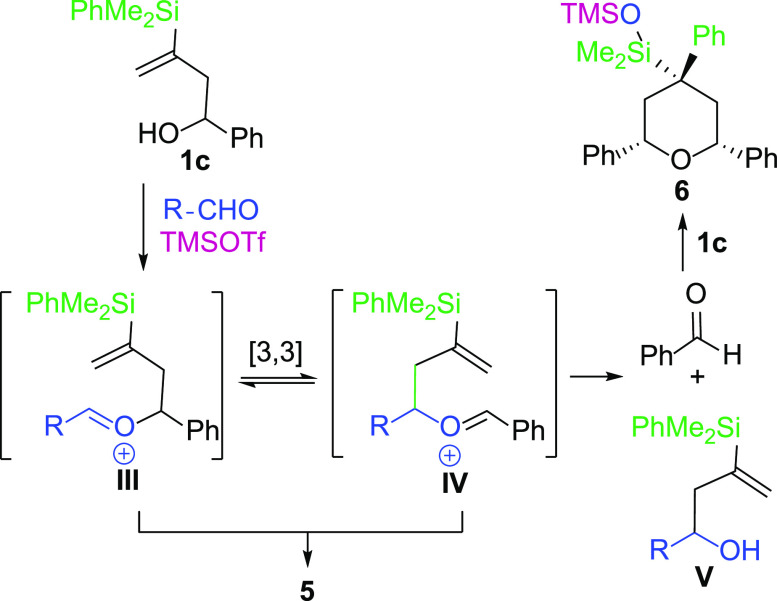
Mechanism of the Oxonia-Cope Rearrangement

As shown in Table 2, the occurrence of the oxonia-Cope side-chain reaction is dependent on the electronic nature of the aldehyde used. Thus, the use of very reactive alkylic aldehydes (Table 2, entries 1–3) seems to favor the silyl-Prins cyclization vs the oxonia-Cope rearrangement, since tetrahydropyrans **5** are obtained as the almost unique products. This result is interesting, since both Willis^18^ and Martín^19^ have reported that the reaction of benzylic alcohols with alkylic aldehydes provides mixtures of the Prins and the oxonia-Cope products with no or moderate predominance of the Prins derivative.

However, in the presence of less reactive aldehydes (arylic or vinylic) the competence of the side reaction is relevant (Table 2, entries 4–11). In order to try to diminish the amount of the side product, we then decided to change the order of addition of reagents: thus, by adding the alcohol **1c** to a mixture of the aldehyde and Lewis acid we could decrease the possibility of reaction of benzaldehyde with it. In fact, under these conditions we could obtain a slight increase in the ratio of **5** toward **6** (Table 2, entry 6). We next reasoned that the use of an excess of aldehyde (2 equiv instead of 1) could further decrease the chances of formation of the oxonia-Cope product **6**. The results are in accordance with our hypothesis, since the oxonia-Cope product is obtained as the minor product when an excess (2 equiv) of aldehyde is employed (Table 2, entries 6, 7 and 10, 11).

Finally, we decided to explore the scope of this interesting domino reaction by studying the electronic effect of the migrating group in the process. For that purpose, we synthesized vinylsilyl alcohols bearing either electron-rich or electron-poor aryl groups bonded to silicon. The results are shown in Scheme 5.

**Scheme 5:**
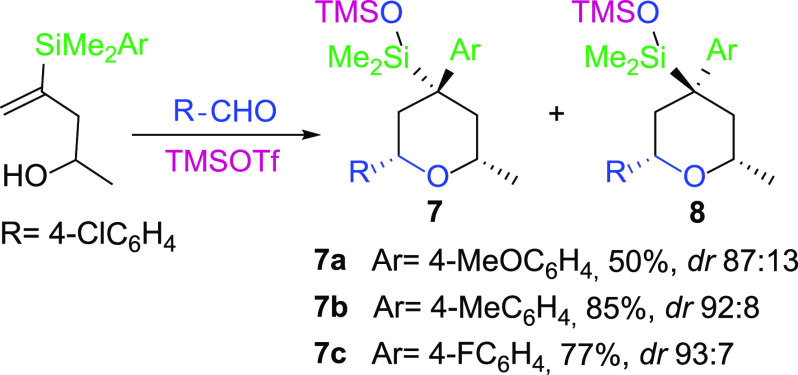
Electronic Effect of the Migrating Group

As shown in Scheme 5, the migration process is effective for all types of arylic groups bonded to silicon (either electron-rich or electron-poor), which significantly broadens the outcome of this interesting process. There is special interest in the reaction of the 4-fluorophenyl derivative **7c**, since Saikia^20^ has reported that the synthesis of 4-aryl tetrahydropyrans, through a Prins–Friedel–Crafts process, is limited to the use of electron-rich aryl nucleophiles.

In summary, we herein report a novel access to 2,4-diaryl tetrahydropyrans through a multicomponent reaction in which a quaternary stereocenter is created with high stereocontrol through a 1,2-Si to C migration of a phenyl group. The process is general and high-yielding for alkylic alcohols and a variety of aldehydes. Moreover, when a benzylic alcohol is used in the process, an oxonia-Cope rearrangement competes with the direct cyclization, whose occurrence is dependent on the nature of the aldehyde employed and on the use or not of an excess of aldehyde. Therefore, the use of alkylic aldehydes or an excess of the corresponding arylic or vinylic aldehydes gives the Prins product almost exclusively or in a very predominant manner.

## Supporting Information Available

Experimental details, characterization data and NMR spectra for new compounds (PDF)

## Accession Codes

CCDC 2042835 contains the supplementary crystallographic data for this paper. These data can be obtained free of charge via www.ccdc.cam.ac.uk/data_request/cif, or by emailing data_request@ccdc.cam.ac.uk, or by contacting The Cambridge Crystallographic Data Centre, 12 Union Road, Cambridge CB2 1EZ, UK; fax: +44 1223 336033.
