# Supplementary material for: Unexpected Domino Silyl-Prins/Aryl Migration Process from Geminal Vinylsilyl Alcohols
Source: Org Lett. 2021 Oct 7;23(21):8385–9. doi: 10.1021/acs.orglett.1c03121 (PMC8576834; doi:10.1021/acs.orglett.1c03121)
Supplement: Supplementary file 1 — ol1c03121_si_001.pdf [file ol1c03121_si_001.pdf]

Supporting Information

Unexpected Domino Silyl-Prins/Aryl Migration Process from Geminal Vinylsilyl Alcohols

Carlos Díez-Poza and Asunción Barbero*

Department of Organic Chemistry, Faculty of Science, University of Valladolid, Campus Miguel Delibes, 47011, Valladolid, SPAIN.
E-mail: asuncion.barbero@uva.es

Web: http://organosilanesorganicsynthesis.blogs.uva.es/

Table of Contents

1. General Procedures S-1

2. Experimental Section S-1

2.1. TMSOTf-promoted cyclization of alcohol **1a-b** S-1

2.2. TMSOTf-promoted cyclization of alcohol **1c** and alkylic aldehydes S-9

2.3. TMSOTf-promoted cyclization of alcohol **1c** and arylic aldehydes S-10

2.4. TMSOTf-promoted cyclization of alcohols **1d-f** S-12

3. X-Ray Crystallographic Data of compound **2e** S-15

4. Copies of NMR Spectra S-17

5. References S-33

1. **GENERAL PROCEDURES**

Unless otherwise noted, experiments were carried out with dry solvents under nitrogen atmosphere. Dichloromethane was dried with preactivated molecular sieves. Flash column chromatography was performed using Silica Gel 60 (230-400 mesh ASTM). Thin layer chromatography (TLC) was performed using aluminium backed plate, pre-coated with silica gel (0.20 mm, silica gel 60) with a fluorescent indicator (254 nm) from Macherey. NMR spectra were recorded at nuclear magnetic resonance service of the Laboratory of Instrumental Techniques (L.T.I., www.laboratoriotecnicasinstrumentales.es) University of Valladolid at Varian 400 MHz (1 H, 399.85 MHz; 13C, 100.61 MHz), Varian 500 MHz (^{1}H, 500.12 MHz; ^{13}C, 100.61 MHz) spectrometers at room temperature (25 ºC). Chemical shifts (δ) were reported in parts per million (ppm) relative to the residual solvent peaks recorded, rounded to the nearest 0.01 for 1 H-NMR and 0.1 for ^{13}C-NMR (reference: $CDCl_3$ [^{1}H: 7.26, ^{13}C: 77.2]). Spin-spin coupling constants (J) in ^{1}H-NMR were given in Hz to the nearest 0.1 Hz, and peak multiplicity was indicated as follows s (singlet), d (doublet), t (triplet), q (quartet), m (multiplet) and br (broad). ^{13}C NMR were recorded with complete proton decoupling. Carbon types, structure assignments and attribution of peaks were determined from two-dimensional correlation experiments (HSQC, COSY and HMBC). Relative stereochemistry was assigned based on the 2D-NOE experiments. High-resolution mass spectra (HRMS) were measured at mass spectrometry service of the Laboratory of Instrumental Techniques, University of Valladolid, on a UPLC-MS system (UPLC: Waters ACQUITY H-class UPLC; MS: Bruker Maxis Impact) by electrospray ionization (ESI positive and negative).

2. EXPERIMENTAL SECTION

2.1. TMSOTf-promoted cyclization of alcohol 1a-b

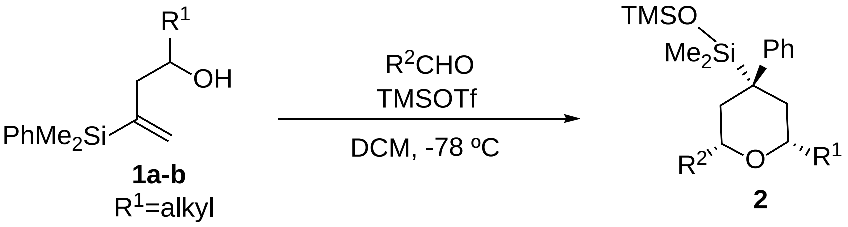

A solution of 100 mg (0.454 mmol) of the homoallylic alcohol **1a** and the corresponding aldehyde (0.499 mmol) in 4.5 ml dichloromethane is cooled to -78 °C (under nitrogen). Then, 0.123 ml TMSOTf (0.681 mmol, 1.5 equiv) is added dropwise. The mixture is stirred for 30 min-1 hour while monitored by TLC. When starting materials are consumed, it is hydrolyzed with 4.5 ml of NaOH (aq) 2M. Phases are then separated, extracting the aqueous phase three times with dichloromethane. The organic phases are combined and dried over anhydrous $MgSO_4$. The solvent is then evaporated under reduced pressure. The crude mixture is analyzed by NMR and then purified by column chromatography in silica gel, using mixtures of hexane-ethyl acetate, yielding tetrahydropyrans **2**.

Compound 2a: (2S*, 4R*, 6S*)-2-methyl-4-(dimethyl(trimethylsilyloxy)silyl)-4,6-diphenyltetrahydropyran.

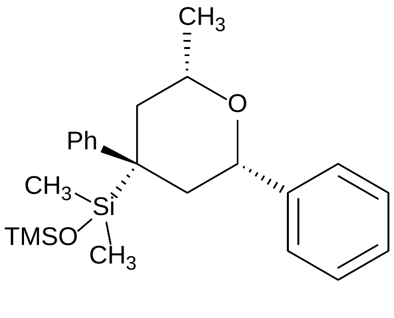

According to the general procedure, the title compound **2a** was obtained from alcohol **1a** (100 mg, 0.454 mmol) and benzaldehyde to give, after column chromatography (hexane/EtOAc: 30:1), a colorless oil (127 mg, 70%). **^{1}H NMR** (500 MHz, $CDCl_3$) δ 7.38 – 7.31 (m, 6H), 7.29 – 7.23 (m, 3H) 7.18 – 7.13 (m, 1H), 4.51 (dd, *J* = 11.2 and 1.9 Hz, 1H), 3.81 – 3.74 (m, 1H), 2.41 (dt, *J* = 14.2 and 1.9 Hz, 1H), 2.30 (dt, *J* = 14.3 and 2.1 Hz, 1H), 1.92 (dd, *J* = 14.2, 11.2 Hz, 1H), 1.74 (dd, *J* = 14.3, 11.0 Hz, 1H), 1.24 (d, *J* = 6.2 Hz, 3H), 0.01 (s, 9H), -0.09 (s, 3H), -0.09 (s, 3H).

^{13}C NMR (101 MHz, $CDCl_3$) δ 143.6 (C), 142.5 (C), 128.3 (CH), 127.6 (CH), 127.2 (CH), 125.9 (CH), 124.3 (CH), 74.8 (CH), 68.9 (CH), 37.2 (CH_2), 36.3 (CH_2), 34.7 (C), 22.3 (CH_3), 1.8 (CH_3SiO), -3.2 (CH_3Si).

HRMS (ESI+) *m/z* calc. for $C_{23}H_{34}O_2Si_2Na$ ($[M+Na]^+$): 421.1990, found 421.1997.

Compound 2b: (2S*, 4R*, 6S*)-2-methyl-4-(dimethyl(trimethylsilyloxy)silyl)-4-phenyl-6-(*p*-tolyl)tetrahydropyran.

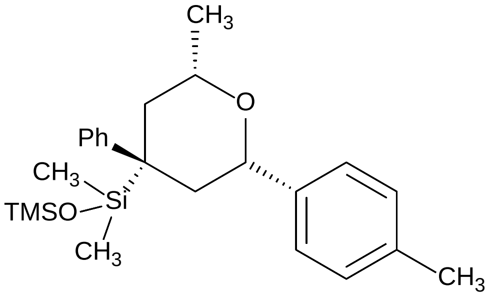

According to the general procedure, the title compound **2b** was obtained from alcohol **1a** (100 mg, 0.454 mmol) and 4-tolualdehyde to give, after column chromatography (hexane/EtOAc: 30:1), a colorless oil (103 mg, 55%). **^{1}H RMN** (500 MHz, $CDCl_3$) δ 7.37 – 7.32 (m, 2H), 7.28 – 7.23 (m, 4H), 7.17 – 7.12 (m, 3H), 4.48 (dd, *J* = 11.2, 1.7 Hz, 1H), 3.80 – 3.72 (m, 1H), 2.39 (dt, *J* = 14.3, 2.2 Hz, 1H), 2.34 (s, 3H), 2.29 (dt, *J* = 14.1, 2.1 Hz, 1H), 1.92 (dd, *J* = 14.3, 11.2 Hz, 1H), 1.73 (dd, *J* = 14.1, 11.0 Hz, 1H), 1.23 (d, *J* = 6.2 Hz, 3H), 0.01 (s, 9H, $(CH_3)_3Si$), -0.09 (s, 6H, $(CH_3)_2Si$).

^{13}C RMN (101 MHz, $CDCl_3$) δ 142.5 (C), 140.7 (C), 136.8 (C), 129.0 (CH), 128.3 (CH), 127.6 (CH), 125.8 (CH), 124.2 (CH), 74.6 (CH), 68.9 (CH), 37.1 (CH_2), 36.3 (CH_2), 34.7 (C), 22.3 (CH_3), 21.1 (CH_3), 1.8 (CH_3SiO), -3.3 (CH_3Si).

HRMS (ESI+) *m/z* calc. for $C_{24}H_{36}O_2Si_2Na$ ($[M+Na]^+$): 435.2146, found 435.2165.

Compound 2c: (2S, 4R, 6S)-2-(*p*-methoxyphenyl)-6-methyl-4-(dimethyl(trimethylsilyloxy)silyl)-4-phenyl-tetrahydropyran.

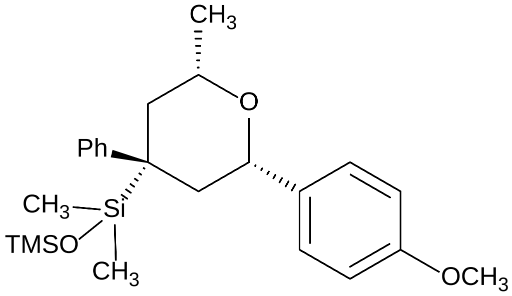

Compound **2c** was prepared in a 2.4 mmol scale, as follow: a solution of 0.531 g (2.41 mmol) of the homoallylic alcohol **1a** and anisaldehyde (2.89 mmol, 1.2 equiv) in 24 ml dichloromethane is cooled to -78 °C (under nitrogen). Then, 0.653 ml TMSOTf (3.61 mmol, 1.5 equiv) is added dropwise. The mixture is stirred while followed by TLC. When starting materials are consumed (ca. 30 min), it is hydrolyzed with 20 ml of NaOH (aq) 2M. Phases are then separated, extracting the aqueous phase three times with dichloromethane. The organic phases are combined and dried over anhydrous $MgSO_4$. The solvent is then evaporated under reduced pressure. The crude mixture is purified by column chromatography in silica gel, using hexane-ethyl acetate (30:1), affording tetrahydropyran **2c** as a colorless oil (0.669 g, 1.56 mmol, 65%).

^{1}H RMN (400 MHz, $CDCl_3$) δ 7.37 – 7.20 (m, 6H), 7.16 – 7.09 (m, 1H), 6.86 (d, *J* = 8.8 Hz, 2H), 4.44 (d, *J* = 11.2 Hz, 1H), 3.78 (s, 3H, CH_3O), 3.77 – 3.70 (m, 1H, C*H*Me), 2.36 (d, *J* = 14.3 Hz, 1H, CHPh), 2.27 (d, *J* = 14.1 Hz, 1H), 1.91 (dd, *J* = 14.3, 11.2 Hz, 1H), 1.71 (dd, *J* = 14.1, 10.9 Hz, 1H), 1.21 (d, *J* = 6.2 Hz, 3H, CH_3), -0.00 (s, 9H, $(CH_3)_3SiO$), -0.11 (s, 6H, $(CH_3)_2Si$).

^{13}C RMN (101 MHz, $CDCl_3$) δ 158.8 (C), 142.5 (C), 135.9 (C), 128.3 (CH), 127.6 (CH), 127.2 (CH), 124.2 (CH), 113.7 (CH), 74.4 (CH), 68.9 (CH), 55.3 (CH_3O), 36.9 (CH_2), 36.3 (CH_2), 34.7 (C), 22.3(CH_3), 1.8 (CH_3SiO), -3.3 (CH_3Si).

HRMS (ESI+) *m/z* calc. for $C_{24}H_{36}O_3Si_2Na$ ($[M+Na]^+$): 451.2095, found 451.2107.

Compound 2d: (2S*, 4R*, 6S*)-2-(*p*-chlorophenyl)-6-methyl-4-(dimethyl(trimethylsilyloxy)silyl)-4-phenyltetrahydropyran.

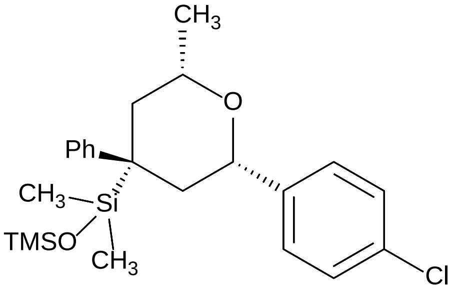

According to the general procedure, the title compound **2d** was obtained from alcohol **1a** (100 mg, 0.454 mmol) and 4-chlorobenzaldehyde to give, after column chromatography (hexane/EtOAc: 30:1), a colorless oil (161 mg, 82%). **^{1}H RMN** (400 MHz, $CDCl_3$) δ 7.35 – 7.20 (m, 8H), 7.15-7.12 (m, 1H), 4.46 (d, *J* = 11.2 Hz, 1H), 3.79 – 3.70 (m, 1H), 2.35 (d, *J* = 14.1 Hz, 1H), 2.28 (d, *J* = 14.3 Hz, 1H), 1.82 (dd, *J* = 14.1, 11.2 Hz, 1H), 1.70 (dd, *J* = 14.3, 11.0 Hz, 1H), 1.21 (d, *J* = 6.2 Hz, 3H), -0.01 (s, 9H), -0.11 (s, 6H).

^{13}C RMN (101 MHz, $CDCl_3$) δ 142.3 (C), 142.2 (C), 132.8 (C), 128.4 (CH), 128.4 (CH), 127.5 (CH), 127.2 (CH), 124.4 (CH), 74.1 (CH), 68.0 (CH), 37.3 (CH_2), 36.2 (CH_2), 34.6 (C), 22.2 (CH_3), 1.8 (CH_3SiO), -3.2 (CH_3Si).

HRMS (ESI+) *m/z* calc. for $C_{23}ClH_{33}NaO_2Si_2$ ($[M+Na]^+$): 455.1600, found 455.1610.

Compound 2e: (2S*, 4R*, 6S*)-2-(4-nitrophenyl)-6-methyl-4-(dimethyl(trimethylsilyloxy)silyl)-4-phenyltetrahydropyran.

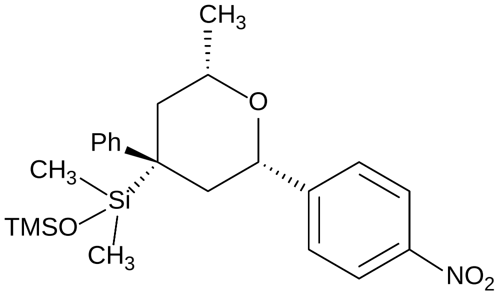

Major isomer:

According to the general procedure, the title compound **2e** was obtained from alcohol **1a** (100 mg, 0.454 mmol) and 4-nitrobenzaldehyde to give, after column chromatography (hexane/EtOAc: 30:1), a colorless oil (91mg, 45%). **^{1}H NMR** (400 MHz, $CDCl_3$) δ 8.23 – 8.12 (m, 2H), 7.54 – 7.46 (m, 2H), 7.39 – 7.31 (m, 2H), 7.28 – 7.21 (m, 2H), 7.19 – 7.13 (m, 1H), 4.59 (d, *J* = 11.1 Hz, 1H), 3.84 – 3.71 (m, 1H), 2.40 (d, *J* = 14.1 Hz, 1H), 2.31 (d, *J* = 14.4 Hz, 1H), 1.79 (dd, *J* = 14.1, 11.1 Hz, 1H), 1.71 (dd, *J* = 14.4, 11.0 Hz, 1H), 1.25 (d, *J* = 6.3 Hz, 3H), -0.01 (s, 9H), -0.09 (s, 3H), -0.11 (s, 3H).

^{13}C NMR (101 MHz, $CDCl_3$) δ 151.2 (C), 147.0 (C), 142.0 (C), 128.5 (CH), 127.4 (CH), 126.4 (CH), 124.6 (CH), 123.6 (CH), 73.9 (CH), 69.0 (CH), 37.7 (CH_2), 36.1 (CH_2), 34.6 (C), 22.1 (CH_3), 1.8 (CH_3SiO), -3.3 (CH_3Si).

HRMS (ESI+) *m/z* calc. for $C_{23}H_{33}NNaO_4Si_2$ ($[M+Na]^+$): 466.1840, found 466.1838.

Compound 3e: (2S*, 4S*, 6S*)-2-(4-nitrophenyl)-6-methyl-4-(dimethyl(trimethylsilyloxy)silyl)-4-phenyltetrahydropyran.

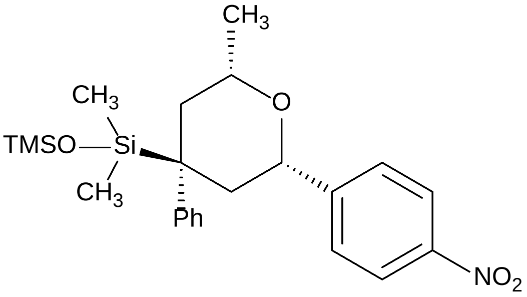

Minor isomer:

^{1}H NMR (400 MHz, $CDCl_3$) δ 8.20 (d, *J* = 8.9 Hz, 2H), 7.53 (d, *J* = 8.9 Hz, 2H), 7.24 – 7.18 (m, 2H), 7.13 – 7.05 (m, 3H), 5.07 (dd, *J* = 12.1, 1.6 Hz, 1H), 4.26 – 4.13 (m, 1H), 2.61 (d, *J* = 13.5 Hz, 1H), 2.52 (d, *J* = 13.3 Hz, 1H), 1.59 (dd, *J* = 13.5, 12.1 Hz, 1H), 1.56 – 1.48 (m, 1H), 1.32 (d, *J* = 6.1 Hz, 3H), 0.11 (s, 9H), -0.01 (s, 3H), -0.04 (s, 3H).

^{13}C NMR (101 MHz, $CDCl_3$) δ 151.4 (C), 147.7 (C), 147.1 (C), 127.8 (CH), 126.6 (CH), 126.1 (CH), 125.1 (CH), 123.7 (CH), 75.8 (CH), 70.9 (CH), 41.6 (CH_2), 40.8 (CH_2), 33.6 (C), 22.6 (CH_3), 2.0 (CH_3SiO), -0.8 (CH_3Si), -0.9 (CH_3Si).

HRMS (ESI+) *m/z* calc. for $C_{23}H_{33}NNaO_4Si_2$ ($[M+Na]^+$): 466.1840, found 466.1841.

Compound 2f: (2S*, 4R*, 6S*)-2-methyl-4-(dimethyl(trimethylsilyloxy)silyl)-6-(naphthalen-1-yl)-4-phenyltetrahydropyran.

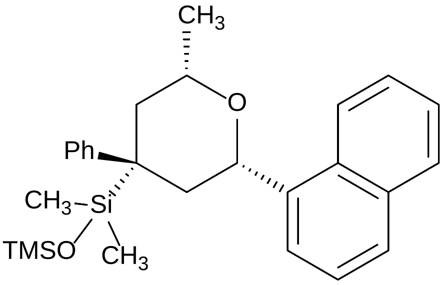

According to the general procedure, the title compound **2f** was obtained from alcohol **1a** (100 mg, 0.454 mmol) and 1-naphtaldehyde to give, after column chromatography (hexane/EtOAc: 30:1), a colorless oil (173 mg, 85%). **^{1}H RMN** (500 MHz, $CDCl_3$) δ 8.05 – 7.98 (m, 1H), 7.89 – 7.83 (m, 1H), 7.81 – 7.74 (m, 1H), 7.74 – 7.69 (m, 1H), 7.54 – 7.39 (m, 5H), 7.39 – 7.34 (m, 2H), 7.25 – 7.18 (m, 1H), 5.25 (d, *J* = 11.1 Hz, 1H), 4.06 – 3.99 (m, 1H), 2.65 – 2.61 (m, 1H), 2.46 – 2.43 (m, 1H), 2.14 (dd, *J* = 14.3, 11.1 Hz, 1H), 1.89 (dd, *J* = 14.2, 11.0 Hz, 1H), 1.36 (d, *J* = 6.2 Hz, 3H, CH_3), 0.05 (s, 9H, $(CH_3)_3SiO$), -0.04 (s, 6H, $(CH_3)_2Si$).

^{13}C NMR (101 MHz, $CDCl_3$) δ 142.6 (C), 139.4 (C), 133.8 (C), 130.5 (C), 128.8 (CH), 128.5 (CH), 127.7 (CH), 127.6 (CH), 125.7 (CH), 125.7 (CH), 125.3 (CH), 124.5 (CH), 123.3 (CH), 72.1 (CH), 69.3 (CH), 36.7 (CH_2), 36.2 (CH_2), 35.0 (C), 22.5 (CH_3), 1.9 (CH_3SiO), -3.2 (CH_3Si).

HRMS (ESI+) *m/z* calc. for $C_{27}H_{36}O_2Si_2Na$ ($[M+Na]^+$):471.2146, found 471.2147.

Compound 2g: (2S*, 4R*, 6S*)-2-(6-bromo-1,3-benzodioxol-5-yl)-2-methyl-4-(dimethyl(trimethylsilyloxy)silyl)-4-phenyltetrahydropyran.

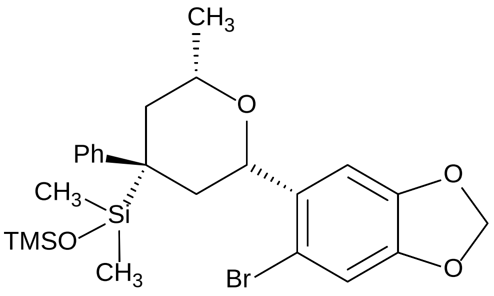

According to the general procedure, the title compound **2g** was obtained from alcohol **1a** (100 mg, 0.454 mmol) and 6-bromopiperonal to give, after column chromatography (hexane/EtOAc: 30:1), a colorless viscous liquid (196 mg, 83%). **^{1}H RMN** (500 MHz, $CDCl_3$) δ 7.41 – 7.29 (m, 4H), 7.17 – 7.11 (m, 2H), 6.95 (s, 1H), 6.00 – 5.91 (m, 2H, OCH_2O), 4.73 (dd, *J* = 11.0, 2.0 Hz, 1H, CH), 3.81 – 3.72 (m, 1H, CH), 2.46 (dt, *J* = 14.3, 2.0 Hz, 1H), 2.29 (dt, *J* = 14.2, 1.9 Hz, 1H), 1.70 (dd, *J* = 14.2, 10.9 Hz, 1H), 1.62 (dd, *J* = 14.3, 11.0 Hz, 1H), 1.23 (d, *J* = 6.1 Hz, 3H, CH_3), 0.01 (s, 9H, $(CH_3)_3SiO$), -0.10 (s, 3H, $(CH_3)_2Si$), -0.11 (s, 3H, $(CH_3)_2Si$).

^{13}C NMR (101 MHz, $CDCl_3$) δ 147.7 (C), 147.3 (C), 141.8 (C), 136.6 (C), 128.1 (CH), 127.9 (CH), 124.4 (CH), 112.2 (CH), 111.5 (C), 107.7 (CH), 101.6 (CH_2), 74.0 (CH), 68.7 (CH), 36.3 (CH_2), 35.9 (CH_2), 34.5 (C), 22.3 (CH_3), 1.8 (CH_3SiO), -3.2 (CH_3Si), -3.3 (CH_3Si).

HRMS (ESI+) *m/z* calc. for $C_{24}H_{33}BrO_4Si_2Na$ ($[M+Na]^+$):543.0993, found 543.1011.

Compound 2h: (2S*, 4R*, 6S*)-2-methyl-4-(dimethyl(trimethylsilyloxy)silyl)-4-phenyl-6-((*E*)-styryl)tetrahydropyran.

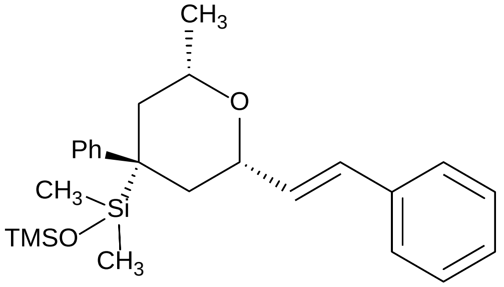

According to the general procedure, the title compound **2h** was obtained from alcohol **1a** (100 mg, 0.454 mmol) and *trans*-cinnamaldehyde to give, after column chromatography (hexane/EtOAc: 30:1), a colorless oil (135 mg, 70%). **^{1}H RMN** (400 MHz, $CDCl_3$) δ 7.41 – 7.07 (m, 10H), 6.55 (dd, *J* = 16.0, 1.2 Hz, 1H), 6.24 (dd, *J* = 16.0, 6.5 Hz, 1H), 4.16 –4.12 (m, 1H), 3.70-3.65 (m, 1H), 2.33 (d, *J* = 14.1 Hz, 1H), 2.25 (d, *J* = 14.3 Hz, 1H), 1.81 (dd, *J* = 14.1, 11.2 Hz, 1H), 1.66 (dd, *J* = 14.3, 11.0 Hz, 1H), 1.22 (d, *J* = 6.2 Hz, 3H), 0.01 (s, 9H), -0.08 (s, 6H).

^{13}C RMN (101 MHz, $CDCl_3$) δ 142.4 (C), 136.9 (C), 130.9 (CH), 130.3 (CH), 128.4 (CH), 128.3 (CH), 127.6 (CH), 127.4 (CH), 126.4 (CH), 124.3 (CH), 73.3 (CH), 68.4 (CH), 36.3 (CH_2), 35.0 (CH_2), 34.1 (C), 22.2 (CH_3), 1.8 (CH_3SiO), -3.2 (CH_3Si).

HRMS (ESI+) *m/z* calc. for $C_{25}H_{36}O_2Si_2Na$ ($[M+Na]^+$): 447.2146, found 447.2153.

Compound 2i: (2S*, 4R*, 6S*)-2-methyl-4-(dimethyl(trimethylsilyloxy)silyl)-6-((*E*)-pent-1-enyl)-4-phenyl-tetrahydropyran.

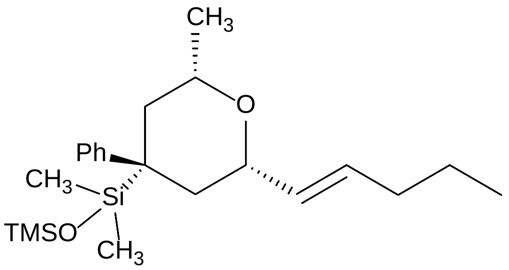

According to the general procedure, the title compound **2i** was obtained from alcohol **1a** (100 mg, 0.454 mmol) and *trans*-2-hexen-1-al to give, after column chromatography (hexane/EtOAc: 30:1), a colorless oil (108 mg, 61%). **^{1}H NMR** (400 MHz, $CDCl_3$) δ 7.31 – 7.25 (m, 2H), 7.18 – 7.14 (m, 2H), 7.12 – 7.06 (m, 1H), 5.62 (dt, *J* = 15.5, 6.5 Hz, 1H), 5.47 (dd, *J* = 15.5, 6.8 Hz, 1H), 3.90 (dd, *J* = 11.1, 6.8 Hz, 1H), 3.64 – 3.53 (m, 1H), 2.22 – 2.15 (m, 2H), 2.02 – 1.95 (m, 2H), 1.70 (dd, *J* = 14.1, 11.1 Hz, 1H), 1.59 (dd, *J* = 14.3, 10.9 Hz, 1H), 1.45 – 1.33 (m, 2H), 1.16 (d, *J* = 6.2 Hz, 3H), 0.89 (t, *J* = 7.3 Hz, 3H), -0.01 (s, 9H), -0.10 (s, 3H), -0.11 (s, 3H).

^{13}C NMR (101 MHz, $CDCl_3$) δ 142.6 (C), 132.0 (CH), 131.5 (CH), 128.2 (CH), 127.6 (CH), 124.1 (CH), 73.3 (CH), 68.2 (CH), 36.3 (CH_2), 34.9 (CH_2), 34.4 (CH_2), 34.0 (C), 22.2 (CH_3), 22.1 (CH_2), 13.8 (CH_3), 1.8 (CH_3SiO), -3.2 (CH_3Si).

HRMS (ESI+) *m/z* calc. for $C_{22}H_{38}O_2Si_2Na$ ($[M+Na]^+$): 413.2303, found 413.2304.

Compound 2j: (2S*, 4R*, 6S*)-2-methyl-4-(dimethyl(trimethylsilyloxy)silyl)-4-phenyl-6-((*E*)-propen-1-enyl)tetrahydropyran.

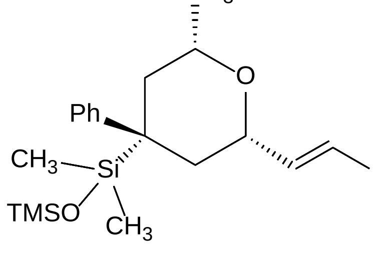

According to the general procedure, the title compound **2j** was obtained from alcohol **1a** (100 mg, 0.454 mmol) and *trans*-crotonaldehyde to give, after column chromatography (hexane/EtOAc: 30:1), a colorless oil (82 mg, 50%). **^{1}H NMR** (400 MHz, $CDCl_3$) δ 7.32 – 7.23 (m, 2H), 7.19 – 7.13 (m, 2H), 7.13 – 7.06 (m, 1H), 5.65 (dq, *J* = 15.4, 6.3 Hz, 1H), 5.50 (dd, *J* = 15.4, 7.0 Hz, 1H), 3.90 (dd, *J* = 10.9, 7.0 Hz, 1H), 3.68 – 3.53 (m, 1H), 2.26 – 2.12 (m, 2H), 1.70 (dd, *J* = 14.3, 10.9 Hz, 1H), 1.68 (d, *J* = 6.3 Hz, 3H, CH_3 crotyl), 1.59 (dd, *J* = 14.4, 10.8 Hz, 1H), 1.16 (d, *J* = 6.2 Hz, 3H), -0.00 (s, 9H), -0.10 (s, 3H), -0.11 (s, 3H).

^{13}C NMR (101 MHz, $CDCl_3$) δ 142.6 (C), 132.7 (CH=), 128.2 (CH ar), 127.6 (CH ar), 127.2 (CH=), 124.1 (CH ar), 73.2 (CH), 68.2 (CH), 36.3 (CH_2), 34.7 (CH_2), 34.0 (C), 22.2 (CH_3), 17.8 (CH_3), 1.8 (CH_3SiO), -3.2 (CH_3Si).

HRMS (ESI+) *m/z* calc. for $C_{20}H_{34}O_2Si_2Na$ ($[M+Na]^+$): 385.1990, found 385.1997.

Compound 2k: (2S*, 4R*, 6S*)-2-cyclohexyl-6-methyl-4-(dimethyl(trimethylsilyloxy)silyl)-4-phenyltetrahydropyran.

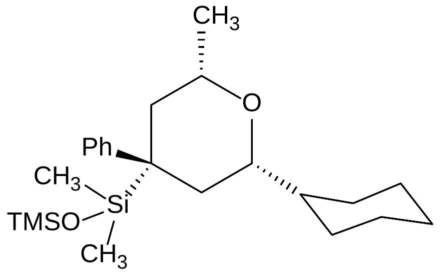

According to the general procedure, the title compound **2k** was obtained from alcohol **1a** (100 mg, 0.454 mmol) and cyclohexanecarboxaldehyde to give, after column chromatography (hexane/EtOAc: 30:1), a colorless oil (92 mg, 50%). **^{1}H RMN** (400 MHz, CDCl3) δ 7.30 – 7.22 (m, 2H), 7.17 – 7.11 (m, 2H), 7.10 – 7.04 (m, 1H), 3.54 – 3.43 (m, 1H), 3.12 (dd, *J* = 10.7, 7.2 Hz, 1H), 2.27 (d, *J* = 14.1, 1H), 2.18 (d, *J* = 14.0, 1H), 1.89 (d, *J* = 13.0 Hz, 1H, cy), 1.80 – 1.63 (m, 4H, cy), 1.59 – 1.49 (m, 2H), 1.39 – 1.28 (m, 1H, cy), 1.27 – 1.17 (m, 3H, cy), 1.12 (d, *J* = 6.2, 3H), 1.01 – 0.95 (m, 1H, cy), 0.94 – 0.88 (m, 1H, cy), 0.00 (s, 9H, $(CH_3)_3Si$), -0.11 (s, 6H, $(CH_3)_2Si$).

^{13}C NMR (101 MHz, $CDCl_3$) δ 143.0 (C), 128.1 (CH), 127.6 (CH), 124.0 (CH), 76.7 (CH), 68.7 (CH), 42.9 (CH, cy), 36.9 (CH_2), 33.9 (C), 31.0 (CH_2), 29.2 (CH_2, cy), 28.6 (CH_2, cy), 26.6 (CH_2, cy), 26.2 (CH_2, cy), 26.1 (CH_2, cy), 22.2 (CH_3), 1.8 (CH_3SiO), -3.2 (CH_3Si).

HRMS (ESI+) *m/z* calc. for $C_{23}H_{40}O_2Si_2Na$ ($[M+Na]^+$): 427.2459, found 427.2465.

Compound 2l: (2S*, 4R*, 6R*)-2-methyl-4-(dimethyl(trimethylsilyloxy)silyl)-4-phenyl-6-phenylmethyltetrahydropyran.

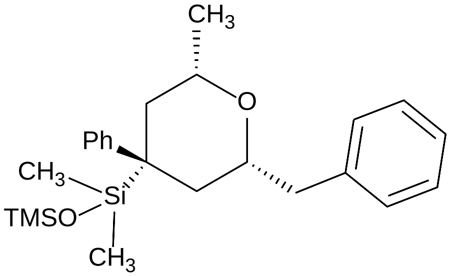

According to the general procedure, the title compound **2l** was obtained from alcohol **1a** (100 mg, 0.454 mmol) and phenylacetaldehyde to give, after column chromatography (hexane/EtOAc: 30:1), a colorless oil (140 mg, 75%). **^{1}H NMR** (400 MHz, $CDCl_3$) δ 7.31 – 7.25 (m, 1H), 7.22 – 7.15 (m, 5H), 7.09 – 7.01 (m, 1H), 6.97 – 6.92 (m, 2H), 3.71 – 3.62 (m, 1H), 3.60 – 3.51 (m, 1H), 2.92 (dd, *J* = 13.5, 6.1 Hz, 1H), 2.59 (dd, *J* = 13.5, 7.4 Hz, 1H), 2.18 (d, *J* = 14.0 Hz, 1H), 2.13 (d, *J* = 14.1 Hz, 1H), 1.59 (dd, *J* = 14.0, 10.9 Hz, 1H), 1.54 (dd, *J* = 14.1, 10.9 Hz, 1H), 1.15 (d, *J* = 6.2 Hz, 3H), -0.04 (s, 9H), -0.14 (s, 3H), -0.18 (s, 3H).

^{13}C NMR (101 MHz, $CDCl_3$) δ 142.6 (C), 138.8 (C), 129.3 (CH), 128.1 (CH), 128.1 (CH), 127.5 (CH), 126.0 (CH), 124.0 (CH), 73.3 (CH), 68.7 (CH), 42.9 (CH_2), 36.7 (CH_2), 34.0 (C), 33.6 (CH_2), 22.1 (CH_3), 1.8 (CH_3SiO), -3.2 (CH_3Si).

HRMS (ESI+) *m/z* calc. for $C_{24}H_{36}O_2Si_2Na$ ($[M+Na]^+$): 435.2146, found 435.2142.

Compound 2m: (2S*, 4R*, 6S*)-4-(dimethyl(trimethylsilyloxy)silyl)-2-phenethyl-4-phenyl-6-((*E*)-styryl)tetrahydropyran.

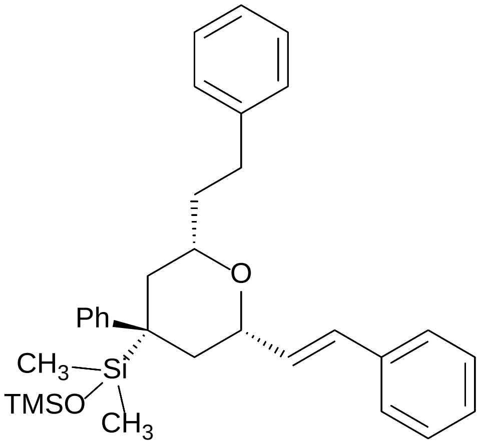

According to the general procedure, the title compound **2m** was obtained from alcohol **1b** (100 mg, 0.322 mmol) and *trans*-cinnamaldehyde to give, after column chromatography (hexane/EtOAc: 30:1), a colorless oil (126 mg, 76%). **^{1}H NMR** (400 MHz, $CDCl_3$) δ 7.45 – 7.37 (m, 2H), 7.36 – 7.10 (m, 13H), 6.58 (d, *J* = 16.1, 1H), 6.28 (dd, *J* = 16.1, 6.1 Hz, 1H), 4.14 (dd, *J* = 11.3, 6.1 Hz, 1H), 3.62 – 3.54 (m, 1H), 2.83 (ddd, *J* = 14.3, 10.4, 5.9 Hz, 1H), 2.66 (ddd, *J* = 14.3, 10.3, 5.8 Hz, 1H), 2.36 (d, *J* = 14.2, 1H), 2.28 (d, *J* = 14.1 Hz, 1H), 1.99 – 1.75 (m, 2H), 1.85 (dd, *J* = 14.2, 11.3 Hz, 1H), 1.71 (dd, *J* = 14.1, 11.0 Hz, 1H), 0.02 (s, 9H, TMSO), -0.06 (s, 3H, $SiCH_3$), -0.08 (s, 3H, $SiCH_3$).

^{13}C NMR (101 MHz, $CDCl_3$) δ 142.4 (C), 142.3 (C), 137.0 (C), 131.1 (*CH*=CHPh), 129.9 (CH=*CH*Ph), 128.5 (CH), 128.4 (CH), 128.3 (CH), 128.3 (CH), 127.6 (CH), 127.4 (CH), 126.4 (CH), 125.6 (CH), 124.3 (CH), 73.2 (CH), 72.0 (CH), 38.1 (CH_2), 35.4 (CH_2), 34.5 (CH_2), 34.0 (C), 31.9 (CH_2), 1.8 (CH_3), -3.1 (CH_3).

HRMS (ESI+) *m/z* calc. for $C_{32}H_{42}NaO_2Si_2$ ($[M+Na]^+$): 537.2616, found 537.2628.

Compound 2n: (2S*, 4R*, 6S*)-2-(*p*-chlorophenyl)-4-(dimethyl(trimethylsilyloxy)silyl)-6-phenethyl-4-phenyltetrahydropyran.

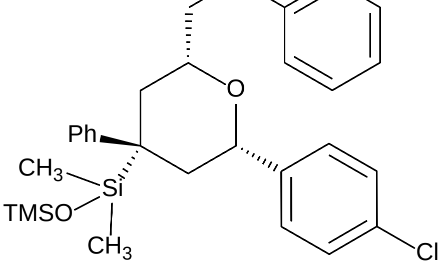

According to the general procedure, the title compound **2n** was obtained from alcohol **1b** (100 mg, 0.322 mmol) and 4-chlorobenzaldehyde to give, after column chromatography (hexane/EtOAc: 30:1), a colorless oil (126 mg, 75%). **^{1}H NMR** (500 MHz, $CDCl_3$) δ 7.37 – 7.12 (m, 14H), 4.46 (dd, *J* = 11.2, 1.8 Hz, 1H), 3.69 – 3.61 (m, 1H), 2.81 (ddd, *J* = 16.1, 10.4, 5.9 Hz, 1H), 2.65 (ddd, *J* = 16.1, 10.4, 5.9 Hz, 1H), 2.41 (dt, *J* = 14.2, 1.8 Hz, 1H), 2.30 (dt, *J* = 14.0, 2.0 Hz, 1H), 1.99 – 1.76 (m, 2H, CH_2), 1.84 (dd, *J* = 14.2, 11.2 Hz, 1H), 1.75 (dd, *J* = 14.0, 11.0 Hz, 1H), 0.00 (s, 9H, TMSO), -0.10 (s, 3H, CH_3-Si), -0.11 (s, 3H, CH_3-Si).

^{13}C NMR (101 MHz, $CDCl_3$) δ 142.4 (C, Ph), 142.3 (C, Ph), 142.2 (C, Ph), 132.7 (C, Ph), 128.4 (CH, Ph), 128.4 (Ch, Ph), 128.4 (CH, Ph), 128.3 (CH, Ph), 127.5 (CH, Ph), 127.1 (CH, Ph), 125.7 (CH), 124.4 (CH), 73.8 (CH), 72.3 (CH), 38.1 (CH_2), 37.6 (CH_2), 34.5 (C), 34.3 (CH_2), 31.8 (CH_2), 1.8 (CH_3, TMSO), -3.2 (CH_3-Si), -3.3 (CH_3-Si).

HRMS (ESI+) *m/z* calc. for $C_{30}H_{39}ClNaO_2Si_2$ ($[M+Na]^+$): 545.2069, found 545.2076.

2.2. TMSOTf-promoted cyclization of alcohol 1c and alkylic aldehydes

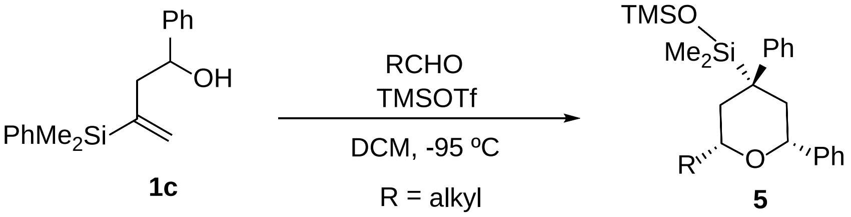

A solution of 100 mg of the benzylic alcohol **1c** and the corresponding aldehyde (1.2 equiv) in dichloromethane (3.5 ml) is cooled to -78 °C (under nitrogen). Then, TMSOTf (1.5 equiv) is added dropwise. The mixture is stirred for 30 min, and then it is hydrolyzed with 7ml of NaOH (aq) 2M. Layers are then separated, extracting the aqueous layer three times with dichloromethane and washing once with brine. The organic phases are combined and dried over $MgSO_4$. Solvent is evaporated under reduced pressure and the crude mixture is then purified by column chromatography in silica gel, using mixtures of hexane-ethyl acetate, yielding tetrahydropyrans **5**.

Compound 5a: (2R*,4S*,6R*)-4-(dimethyl(trimethylsiloxy)silyl)-2,4-diphenyl-6-phenylmethyltetrahydropyran

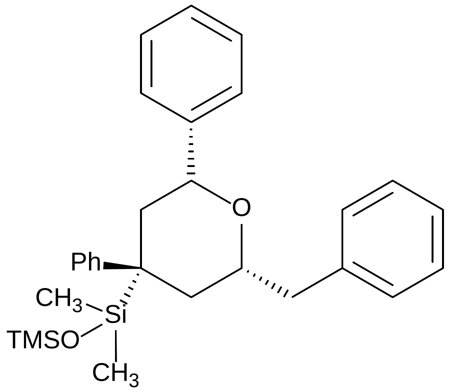

According to the general procedure, the title compound **5a** was obtained from alcohol **1c** (100 mg, 0.354 mmol) and phenylacetaldehyde to give, after column chromatography (hexane/EtOAc: 30:1), a colorless oil (129 mg, 77%). **^{1}H NMR** (500 MHz, $CDCl_3$) δ 7.40 – 7.34 (m, 4H, Ph), 7.31 – 7.25 (m, 6H, Ph), 7.25 – 7.20 (m, 2H, Ph), 7.14 – 7.09 (m, 3H, Ph), 4.53 (dd, *J* = 11.2, 1.8 Hz, 1H), 3.92 – 3.86 (m, 1H), 3.00 (dd, *J* = 13.6, 5.9 Hz, 1H), 2.75 (dd, *J* = 13.6, 6.9 Hz, 1H), 2.43 (d, *J* = 14.1 Hz, 1H), 2.25 (d, *J* = 14.2 Hz, 1H), 1.89 (dd, *J* = 14.1, 11.2 Hz, 1H), 1.71 (dd, *J* = 14.2, 11.0 Hz, 1H), -0.03 (s, 9H, TMSO), -0.11 (s, 3H, CH_3-Si), -0.15 (s, 3H, CH_3-Si).

^{13}C NMR (101 MHz, $CDCl_3$) δ 143.8 (C, Ph), 142.3 (C, Ph), 138.7 (C, Ph), 129.5 (CH, Ph), 128.3 (CH, Ph), 128.6 (CH, Ph), 128.1 (CH, Ph), 127.5 (CH, Ph), 127.1 (CH, Ph), 126.1 (CH, Ph), 125.7 (CH, Ph), 124.6 (CH, Ph), 74.7 (CH), 73.5 (CH), 42.9 (CH_2), 37.5 (CH_2), 34.4 (C), 33.6 (CH_2), 1.8 (CH_3, TMSO), -3.2 (CH_3-Si).

HRMS (ESI+) *m/z* calc. for $C_{29}H_{38}NaO_2Si_2$ ($[M+Na]^+$): 497.2303, found 497.2305.

Compound 5b: (2R*,4S*,6R*)-4-(dimethyl(trimethylsiloxy)silyl)-2,4-diphenyl-6-(2-phenylethyl)-tetrahydropyran

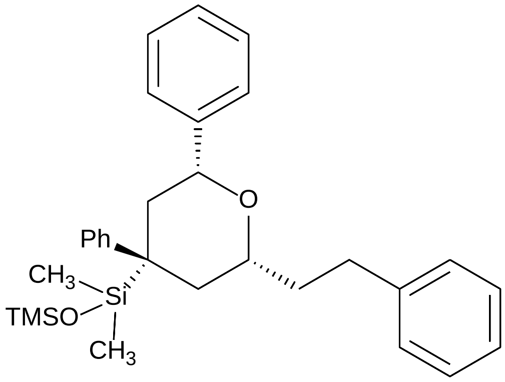

According to the general procedure, the title compound **5b** was obtained from alcohol **1c** (100 mg, 0.354 mmol) and hydrocinnamaldehyde to give, after column chromatography (hexane/EtOAc: 30:1), a colorless oil (95 mg, 55%). **^{1}H NMR** (500 MHz, $CDCl_3$) δ 7.41 – 7.31 (m, 6H, Ph), 7.29 – 7.15 (m, 9H, Ph), 4.51 (d, *J* = 11.1 Hz, 1H), 3.73 – 3.63 (m, 1H), 2.90 – 2.80 (m, 1H), 2.74 – 2.63 (m, 1H), 2.47 (d, *J* = 14.2 Hz, 1H), 2.32 (d, *J* = 14.0 Hz, 1H), 2.00 – 1.94 (m, 1H), 1.92 (dd, *J* = 14.2, 11.1 Hz, 1H), 1.88 – 1.82 (m, 1H, CH**H**), 1.80 (dd, *J* = 14.0, 11.3 Hz, 1H), 0.02 (s, 9H, TMSO), -0.08 (s, 3H, CH_3-Si), -0.09 (s, 3H, CH_3-Si).

^{13}C NMR (126 MHz, $CDCl_3$) δ 143.8 (C), 142.5 (C), 142.4 (C), 128.4 (CH, Ph), 128.4 (CH, Ph), 128.3 (CH, Ph), 127.6 (CH, Ph), 127.1 (CH, Ph), 125.8 (CH, Ph), 125.6 (CH, Ph), 124.3 (CH, Ph), 74.5 (CH), 72.3 (CH), 38.2 (CH_2), 37.4 (CH_2), 34.5 (C), 34.4 (CH_2), 31.8 (CH_2), 1.9 (CH_3, TMSO), -3.2 (CH_3-Si), -3.2 (CH_3-Si).

HRMS (ESI+) *m/z* calc. for $C_{30}H_{40}NaO_2Si_2$ ($[M+Na]^+$): 511.2459, found 511.2467.

2.3. TMSOTf-promoted cyclization of alcohol 1c and arylic or vinylic aldehydes

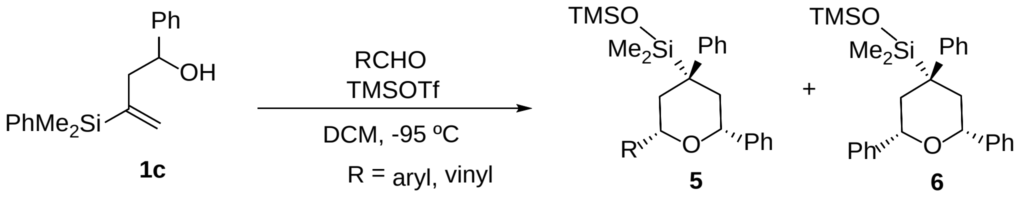

A solution of 0.708 mmol (2 equiv) of the corresponding aldehyde in dichloromethane (3.5 ml) is cooled to -95 °C (under nitrogen). Then, 96 µl TMSOTf (0.531 mmol, 1.5 equiv) is added dropwise. A solution of 100 mg (0.354 mmol) of the alcohol **1c** in 200 µl dichloromethane is also added dropwise**.** The mixture is stirred for 30 min and is then hydrolyzed with 7ml of NaOH (aq) 2M. Layers are then separated, extracting the aqueous layer three times with dichloromethane and washing once with brine. The organic phases are combined and dried over $MgSO_4$. Solvent is evaporated under reduced pressure and the crude mixture is then purified by column chromatography in silica gel, using mixtures of hexane-ethyl acetate, yielding tetrahydropyrans **5** and **6**.

Compound 6: (2S*, 4s, 6R*)-4-(dimethyl(trimethylsilyloxy)silyl)-2,4,6-triphenyltetrahydropyran.

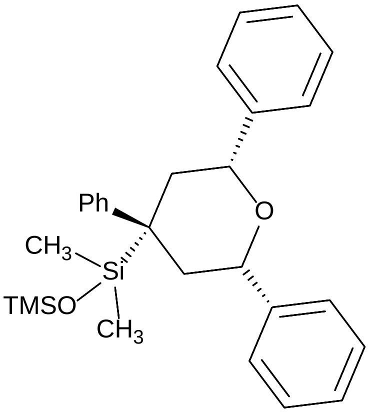

Compound **6** was isolated in small amounts in some of the reactions after column chromatography (hexane/EtOAc: 30:1) and was characterized as follows: **^{1}H NMR** (400 MHz, $CDCl_3$) δ 7.47 – 7.41 (m, 5H, Ph), 7.40 – 7.33 (m, 6H, Ph), 7.30 – 7.18 (m, 4H, Ph), 4.72 (d, J = 11.2 Hz, 2H), 2.53 (d, J = 14.2 Hz, 2H), 2.05 (dd, J = 14.2, 11.2 Hz, 2H), -0.00 (s, 9H, TMSO), -0.09 (s, 6H, 2×CH_3-Si).

^{13}C NMR (101 MHz, $CDCl_3$) δ 143.6 (C, Ph), 142.1 (C, Ph), 128.6 (CH, Ph), 128.3 (CH, Ph), 127.5 (CH, Ph), 127.2 (CH, Ph), 125.9 (CH, Ph), 124.5 (CH, Ph), 75.0 (CH), 37.5 (CH_2), 35.1 (C), 1.8 (CH_3, TMSO), -3.3 (CH_3-Si).

HRMS (ESI+) *m/z* calc. for $C_{28}H_{36}NaO_2Si_2$ ($[M+Na]^+$): 483.2146, found 483.2150.

Compound 5c: (2S*,4R*,6R*)-4-(dimethyl(trimethylsiloxy)silyl)-2-(*p*-methoxyphenyl)-4,6-diphenyltetrahydropyran

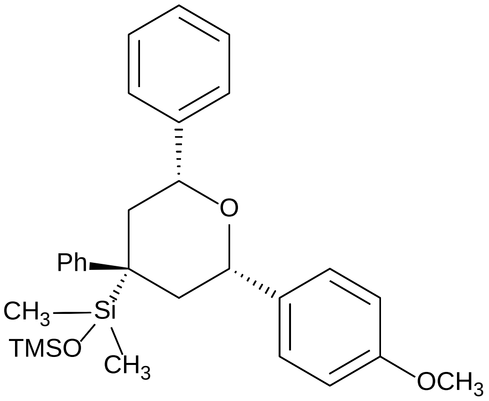

According to the general procedure, the title compound **5c** was obtained from alcohol **1c** (100 mg, 0.354 mmol) and anisaldehyde to give, after column chromatography (hexane/EtOAc: 30:1), a colorless oil (104 mg, 60%). **^{1}H NMR** (400 MHz, $CDCl_3$) δ 7.44 – 7.32 (m, 10H), 7.29 – 7.23 (m, 1H), 7.22 – 7.17 (m, 1H), 6.92 – 6.87 (m, 2H), 4.72 – 4.65 (m, 2H), 3.80 (s, 3H), 2.55 – 2.44 (m, 2H), 2.09 – 1.98 (m, 2H), -0.01 (s, 9H, TMSO), -0.10 (s, 6H, 2×CH_3-Si).

^{13}C NMR (101 MHz, $CDCl_3$) δ 158.8 (C), 143.6 (C), 142.1 (C), 135.9 (C), 128.6 (CH), 128.3 (CH), 127.5 (CH), 127.2 (CH), 127.0 (CH), 125.8 (CH), 124.5 (CH), 113.7 (CH), 75.1 (CH), 74.6 (CH), 55.3 (CH_3O), 37.5 (CH_2), 37.4 (CH_2), 35.1 (C), 1.8 (TMSO), -3.3 (CH_3-Si).

HRMS (ESI+) *m/z* calc. for $C_{29}H_{38}NaO_3Si_2$ ($[M+Na]^+$): 513.2252, found 513.2252.

Compound 5d: (2S*,4R*,6R*)- 2-(*p*-chlorophenyl)-4-(dimethyl(trimethylsiloxy)silyl)-4,6-diphenyltetrahydropyran

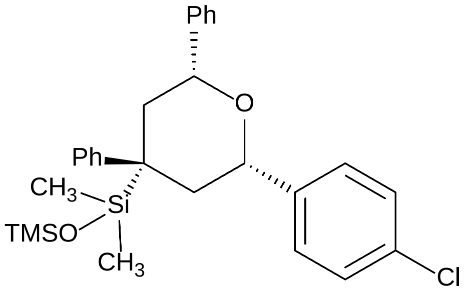

According to the general procedure, the title compound **5d** was obtained from alcohol **1c** (100 mg, 0.354 mmol) and 4-chlorobenzaldehyde to give, after column chromatography (hexane/EtOAc: 30:1), a mixture of the Prins (**5d**) and the Cope (**6**) products as a colorless oil (123 mg, 70%). **^{1}H NMR** (500 MHz, $CDCl_3$) δ (recognisable signals) 4.73 – 4.67 (m, 2H**)**, 2.55 – 2.47 (m, 2H), 2.08 – 1.95 (m, 2H) -0.01 (s, 9H, TMSO), -0.09 (s, 6H, 2×CH_3-Si).

^{13}C NMR (101 MHz, $CDCl_3$) δ (recognisable signals) 143.4 (C, Ph), 141.9 (C, Ph), 128.7 (CH, Ph), 128.4 (CH, Ph), 128.3 (CH, Ph), 127.5 (CH, Ph), 127.1 (CH, Ph), 125.7 (CH, Ph),124.6 (CH, Ph), 75.1 (CH), 74.4 (CH), 37.6 (CH_2), 37.4 (CH_2), 35.0 (C), 1.8 (TMSO), -3.3 (CH_3-Si).

HRMS (ESI+) *m/z* calc. for $C_{28}H_{35}NaClO_2Si_2$ ([M+Na]$^+$): 517.1756, found 517.1762.

Compound 5e:(2S*,4R*,6R*)-4-(dimethyl(trimethylsiloxy)silyl)-2,4-diphenyl-6-((*E*)-styryl)tetrahydropyran

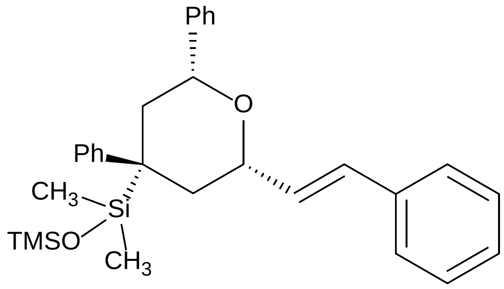

According to the general procedure, the title compound **5e** was obtained from alcohol **1c** (100 mg, 0.354 mmol) and 4-chlorobenzaldehyde to give, after column chromatography (hexane/EtOAc: 30:1), a mixture of the Prins (**5e**) and the Cope (**6**) products as a colorless oil (78 mg, 45%): **^{1}H NMR** (500 MHz, $CDCl_3$) δ 7.52 – 7.16 (m, 15H, Ph), 6.63 (d, *J* = 16.0 Hz, 1H, =C*H*H), 6.36 – 6.30 (m, 1H, =CH*H*), 4.63 (d, *J* = 11.4 Hz, 1H**)**, 4.37 – 4.34 (m, 1H, CH), 2.50 – 2.44 (m, 2H, CH_2), 2.01 – 1.95 (m, 2H, CH_2), -0.02 (s, 9H, TMSO), -0.06 (s, 6H, 2×CH_3-Si).

^{13}C NMR (101 MHz, $CDCl_3$) δ (recognisable signals) 131.0 (CH=), 130.0 (CH=), 74.7 (CH), 73.6 (CH), 37.2 (CH_2), 35.1 (CH_2), 34.6 (C), 1.8 (TMSO), -3.2 (CH_3-Si).

HRMS (ESI+) *m/z* calc. for $C_{30}H_{38}NaO_2Si_2$ ([M+Na]$^+$): 509.2303, found 509.2306.

2.4. TMSOTf-promoted cyclization of alcohols 1d-f

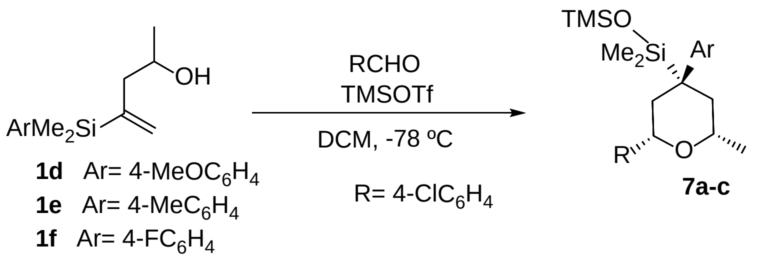

A solution of 100 mg of the homoallylic alcohol **1d-f** and 4-chlorobenzaldehyde (1.1 equiv) in 4.5 ml dichloromethane is cooled to -78 °C (under nitrogen). Then, TMSOTf (1.5 equiv) is added dropwise. The mixture is stirred for 30 min-1 hour while monitored by TLC. When starting materials are consumed, it is hydrolyzed with 4.5 ml of NaOH (aq) 2M. Phases are then separated, extracting the aqueous phase three times with dichloromethane. The organic phases are combined and dried over anhydrous $MgSO_4$. The solvent is then evaporated under reduced pressure. The crude mixture is analyzed by NMR and then purified by column chromatography in silica gel, using mixtures of hexane-ethyl acetate, yielding tetrahydropyrans **7**.

Compound 7a: (2S*, 4R*, 6S*)-2-(*p*-chlorophenyl)-4-(*p*-methoxyphenyl)-6-methyl-4-(dimethyl(trimethylsilyloxy)silyl)-tetrahydropyran.

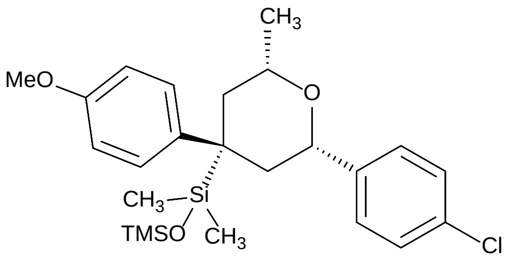

According to the general procedure, the title compound **7a** was obtained from alcohol **1d** (100 mg, 0.399 mmol) and 4-chlorobenzaldehyde to give, after column chromatography (hexane/EtOAc: 30:1), a yellow oil (92 mg, 50%). **^{1}H RMN** (500 MHz, $CDCl_3$) δ 7.32 – 7.27 (m, 4H), 7.16 – 7.12 (m, 2H), 6.92 – 6.89 (m, 2H), 4.48 (dd, *J* = 11.2, 1.8 Hz, 1H), 3.82 (s, 3H, OCH_3), 3.80 – 3.73 (m, 1H), 2.30 (dt, *J* = 14.1, 2.1 Hz, 1H, C*H*H), 2.23 (dt, *J* = 14.3, 1.9 Hz, 1H, C*H*H), 1.83 (dd, J = 14.1, 11.2 Hz, 1H, CH*H*), 1.70 (dd, J = 14.3, 11.0 Hz, 1H, CH*H*), 1.23 (d, *J* = 6.1 Hz, 3H), 0.02 (s, 9H, TMSO), -0.10 (s, 3H, CH_3Si), -0.11 (s, 3H, CH_3Si).

^{13}C RMN (101 MHz, $CDCl_3$) δ 156.6 (C, Ph), 142.3 (C, Ph), 134.0 (C, Ph), 132.7 (C, Ph), 128.5 (CH, Ph), 128.4 (CH, Ph), 127.2 (CH, Ph), 113.9 (CH, Ph), 74.0 (CH), 68.8 (CH), 55.2 (OCH_3), 37.4 (CH_2), 36.3 (CH_2), 33.5 (C), 22.2 (CH_3), 1.9 (CH_3SiO), -3.3 (CH_3Si).

HRMS (ESI+) *m/z* calc. for $C_{24}H_{35}ClNaO_3Si_2$ ($[M+Na]^+$): 485.1705, found 485.1717.

Compound 7b: (2S*, 4R*, 6S*)-2-(*p*-chlorophenyl)-6-methyl-4-(dimethyl(trimethylsilyloxy)silyl)-4-tolyltetrahydropyran.

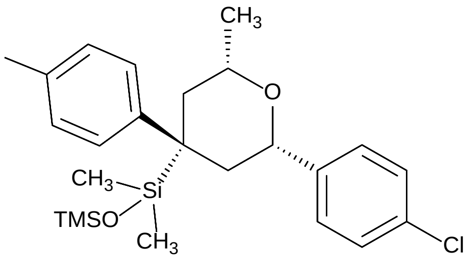

According to the general procedure, the title compound **7b** was obtained from alcohol **1e** (100 mg, 0.427 mmol) and 4-chlorobenzaldehyde to give, after column chromatography (hexane/EtOAc: 30:1), a yellow oil (162 mg, 85%). **^{1}H RMN** (500 MHz, $CDCl_3$) δ 7.31 – 7.27 (m, 4H, Ph), 7.17 – 7.10 (m, 4H, Ph), 4.48 (dd, *J* = 11.2, 1.7 Hz, 1H, CH), 3.79 – 3.72 (m, 1H, CH), 2.34 (s, 3H, CH_3), 2.33 (dt, *J* = 14.2, 2.1 Hz, 1H), 2.25 (dt, *J* = 14.1, 1.8 Hz, 1H, C*H*H), 1.83 (dd, *J* = 14.2, 11.2 Hz, 1H, CH*H*), 1.69 (dd, *J* = 14.1, 11.0 Hz, 1H, CH*H*), 1.22 (d, *J* = 6.1 Hz, 3H, CH_3), 0.01 (s, 9H, TMSO), -0.10 (s, 3H, CH_3Si), -0.11 (s, 3H, CH_3Si).

^{13}C RMN (101 MHz, $CDCl_3$) δ 142.3 (C, Ph), 139.0 (C, Ph), 133.7 (C, Ph), 132.7 (C, Ph), 129.2 (CH, Ph), 128.4 (CH, Ph), 127.4 (CH, Ph), 127.3 (CH, Ph), 74.1 (CH), 68.9 (CH), 37.3 (CH_2), 36.2 (CH_2), 34.1 (C), 22.2 (CH_3), 20.8 (CH_3, tolyl), 1.9 (CH_3SiO), -3.3 (CH_3Si).

HRMS (ESI+) *m/z* calc. for $C_{24}H_{35}ClNaO_2Si_2$ ($[M+Na]^+$): 469.1756, found 469.1759.

Compound 7c: (2S*, 4R*, 6S*)-2-(*p*-chlorophenyl)-4-(*p*-fluorophenyl)-6-methyl-4-(dimethyl(trimethylsilyloxy)silyl)- tetrahydropyran.

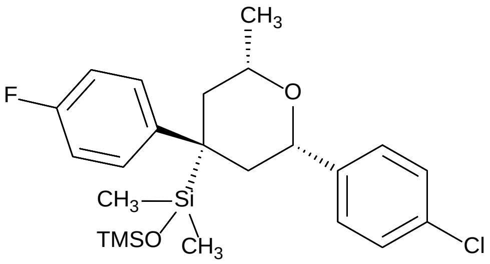

According to the general procedure, the title compound **7c** was obtained from alcohol **1f** (100 mg, 0.419 mmol) and 4-chlorobenzaldehyde to give, after column chromatography (hexane/EtOAc: 30:1), a yellow oil (146 mg, 77%). **^{1}H RMN** (500 MHz, $CDCl_3$) δ 7.33 – 7.26 (m, 4H, Cl-Ph), 7.20 – 7.16 (m, 2H, F-Ph), 7.08 – 7.02 (m, 2H, F-Ph), 4.44 (dd, *J* = 11.2, 2.0 Hz, 1H), 3.77 – 3.70 (m, 1H), 2.30 (dt, *J* = 14.3, 2.0 Hz, 1H,), 2.23 (dt, *J* = 14.3, 2.0 Hz, 1H), 1.84 (dd, *J* = 14.3, 11.2 Hz, 1H), 1.71 (dd, *J* = 14.3, 11.0 Hz, 1H, CH*H*), 1.23 (d, *J* = 6.2 Hz, 3H, CH_3), 0.01 (s, 9H, TMSO), -0.09 (s, 3H, CH_3Si), -0.10 (s, 3H, CH_3Si).

13C RMN (101 MHz, $CDCl_3$) δ 160.2 (d, $^1J_{13C-19F}$ = 243.5 Hz, C, Ph), 142.0 (C, Ph), 137.9 (d, $J_{13C-19F}$ = 3.1 Hz, C, Ph), 132.9 (C, Ph), 128.8 (d, $^3J_{13C-19F}$ = 7.5 Hz, CH, Ph), 128.5 (CH, Ph), 127.2 (CH, Ph), 115.2 (d, $^2J_{13C-19F}$ = 20.7 Hz, CH, Ph), 74.0 (CH), 68.8 (CH), 37.5 (CH_2), 36.3 (CH_2), 34.1 (C), 22.2 (CH_3), 1.8 (CH_3SiO), -3.3 (CH_3Si).

HRMS (ESI+) *m/z* calc. for $C_{23}H_{32}ClFNaO_2Si_2$ ($[M+Na]^+$): 473.1506, found 473.1507.

3. X-Ray Crystallographic Data of compound 2e

The crystal structure has been deposited at the Cambridge Crystallographic Date Center and allocated the deposition number CCDC: 2042835. This data can be obtained free of charge from the Cambridge Crystallographic Date Center via www.ccdc.cam.ac.uk/data_request/ci

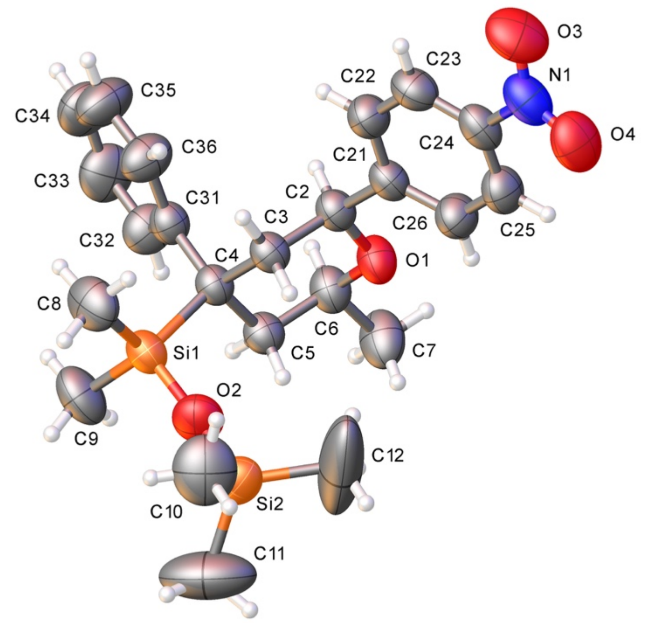

Figure 1. X-ray crystal structure of (2S*, 4R*, 6S*)-6-(4-nitrophenyl)-2-methyl-4-(dimethyl(trimethylsilyloxy)silyl)-4-phenyltetrahydropyran **2e**

Experimental

Single crystals of $C_{23}H_{33.99}NO_{4.5}Si_2$ (**2e**) were grown from a hexane-dichloromethane (50:50) saturated solution, by slow evaporation. A suitable crystal was selected kept at 296.15 K during data collection. Diffraction data were collected using an Oxford Diffraction Supernova diffractometer equipped with an Atlas CCD area detector and a four-circle kappa goniometer. For the data collection, Cu micro-focused source with multilayer optics was used. Data integration, scaling, and empirical absorption correction were performed using the CrysAlisPro software package.[2] The structure was solved by direct methods and refined by full-matrix-least-squares against F2 with SHELX[3] in OLEX2.[4] Non-hydrogen atoms were refined anisotropically, and hydrogen atoms were placed at idealized positions and refined using the riding model. Graphic was made using MERCURY.[5]

X-Ray crystallographic data of **2e** (Figure 1). Crystal data and structure refinement for **2e**

Bond precision: C-C = 0.0036 A Wavelength=1.54184

Cell:	a=9.3870(3)	b=16.8052(6)	c=17.3430(7)
	alpha=82.085(3)	beta=82.880(3)	gamma=78.425(3)
Temperature:	296 K		

	Calculated	Reported
Volume	2641.58(17)	2641.58(17)
Space group	P -1	P -1
Hall group	-P 1	-P 1
Moiety formula	C23 H33 N O4 Si2, 0.034(H4 O2), 0.425(H2 O)	C23 H33 N O4 Si2, 0.5(H2 O)
Sum formula	C23 H33.99 N O4.49 Si2	C23 H33.99 N O4.50 Si2
Mr	452.57	452.64
Dx,g cm-3	1.138	1.138
Z	4	4
Mu (mm-1)	1.449	1.449
F000	971.7	972.0
F000'	976.32	
h,k,lmax	11,21,21	11,20,21
Nref	10910	10533
Tmin,Tmax	0.629,0.693	0.347,1.000
Tmin'	0.391	

Correction method= # Reported T Limits: Tmin=0.347 Tmax=1.000
AbsCorr = GAUSSIAN

Data completeness= 0.965 Theta(max)= 75.217

R(reflections)= 0.0504(7989) wR2(reflections)= 0.1504(10533)

S = 1.044 Npar= 620

4. NMR spectra of compounds

¹H NMR (500 MHz, CDCl₃)

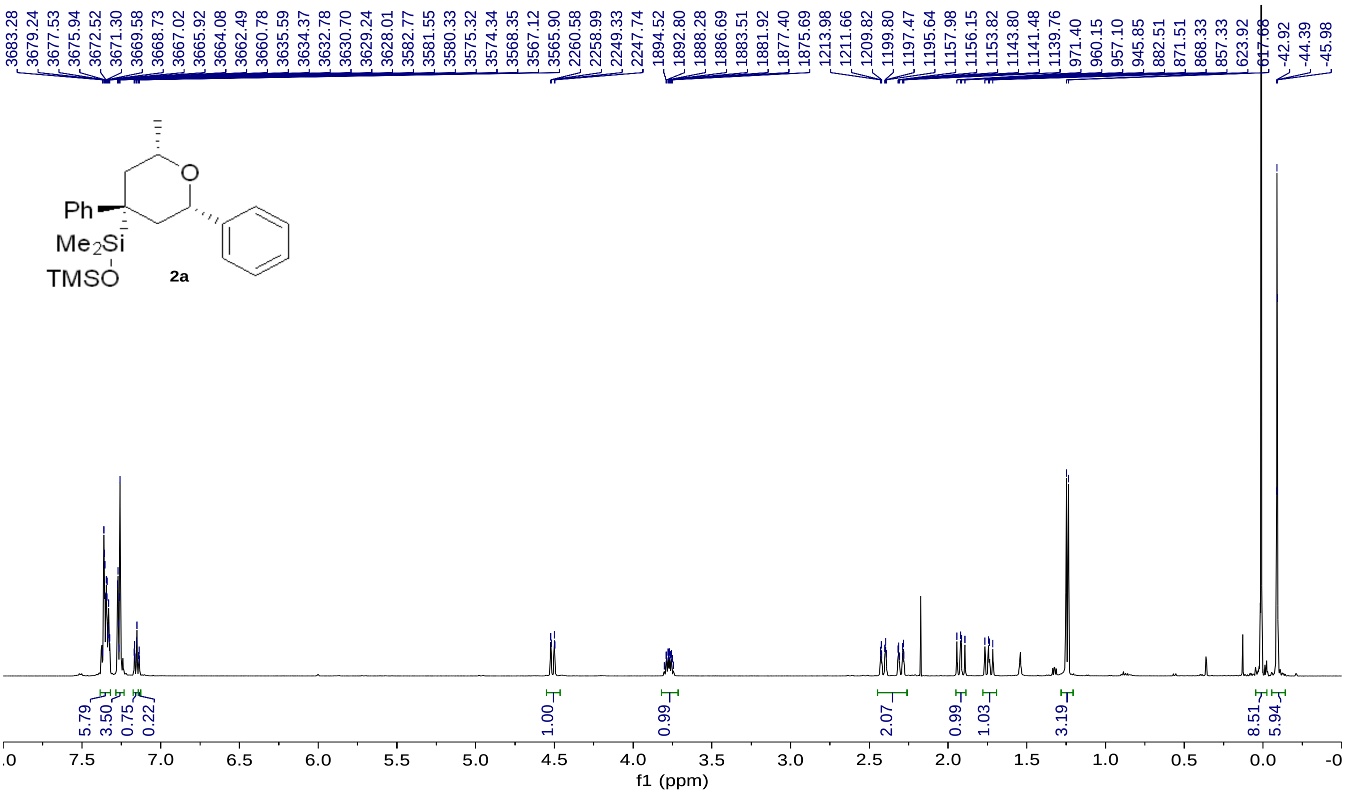

¹³C NMR (101 MHz, CDCl₃)

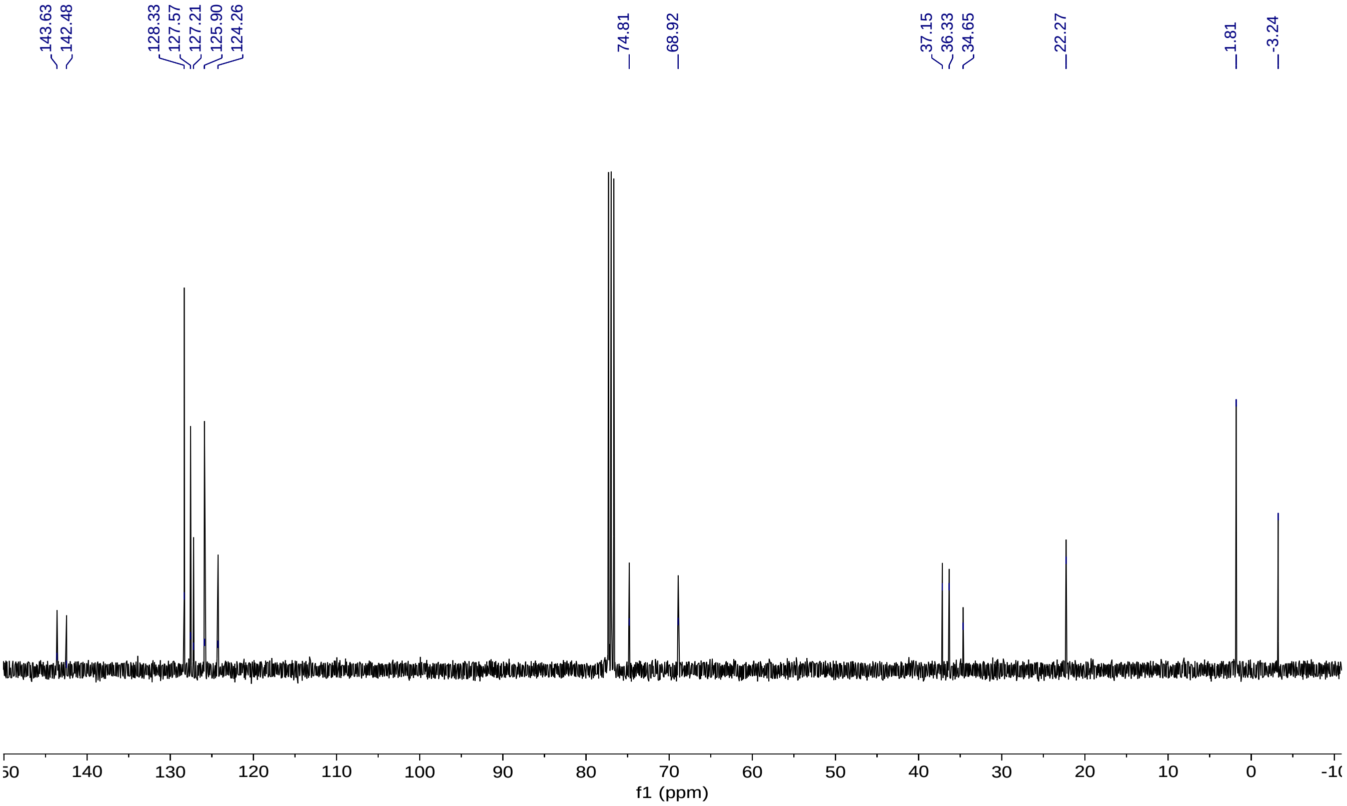

^{1}H NMR (500 MHz, $CDCl_3$)

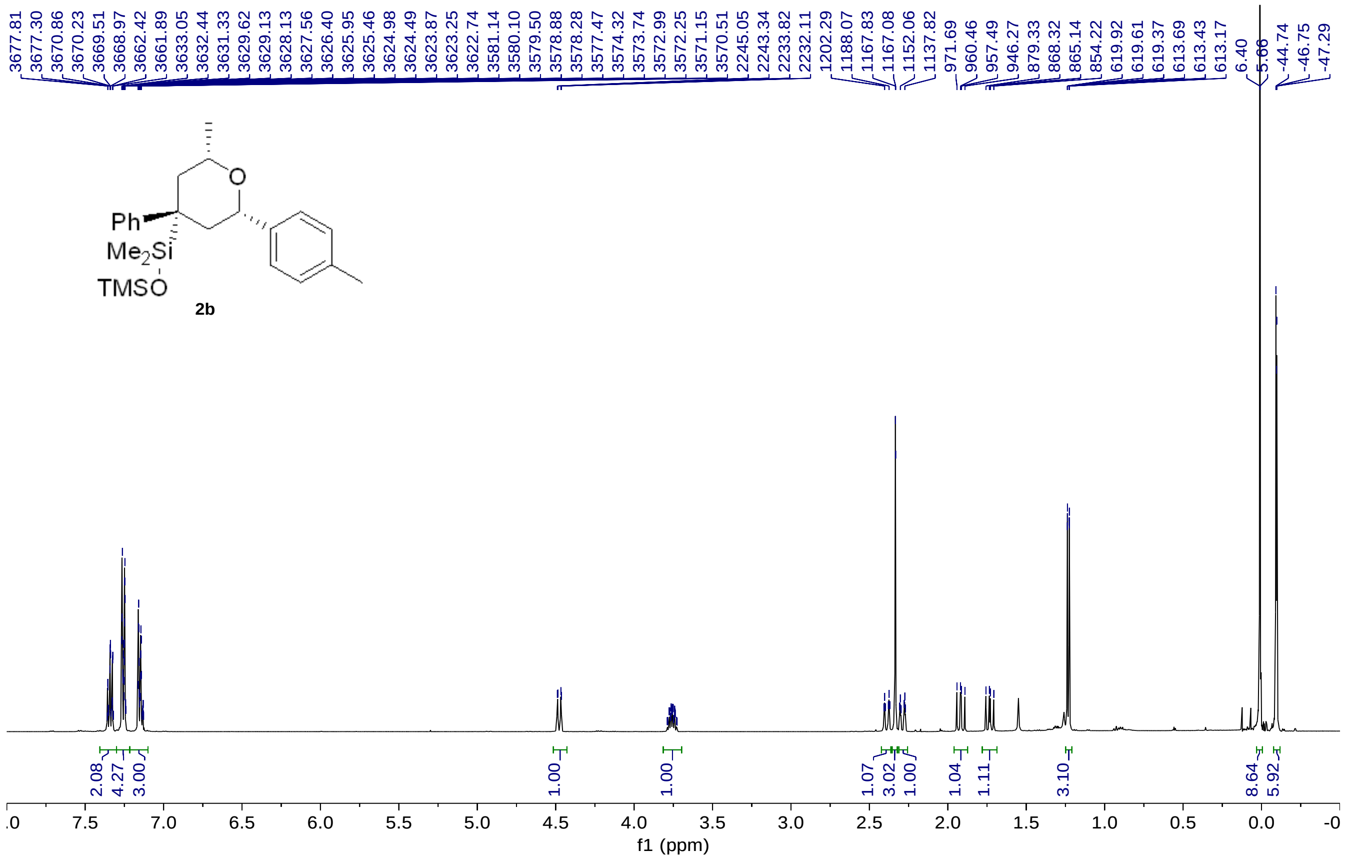

^{13}C NMR (101 MHz, $CDCl_3$)

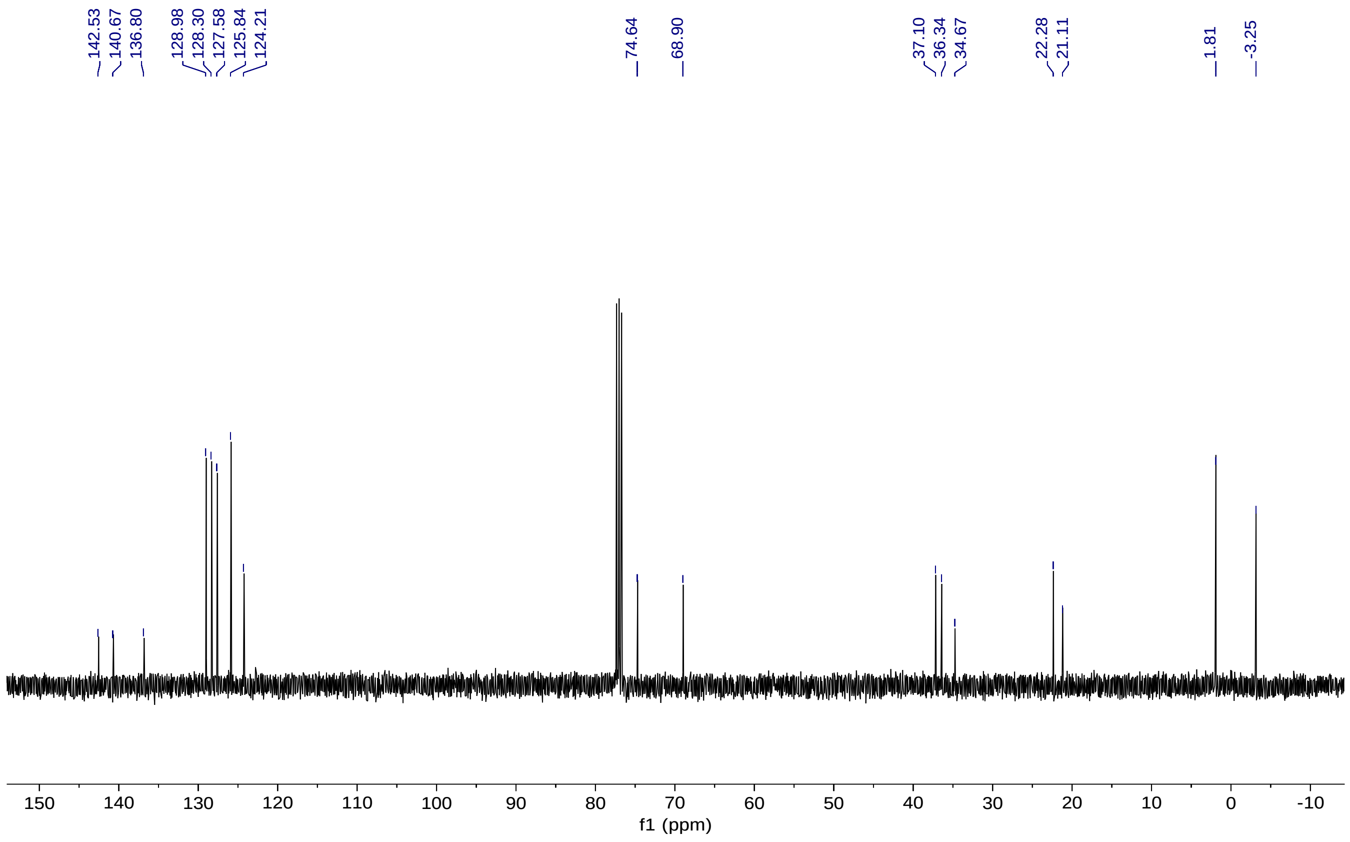

^{1}H NMR (400 MHz, CDCl3)

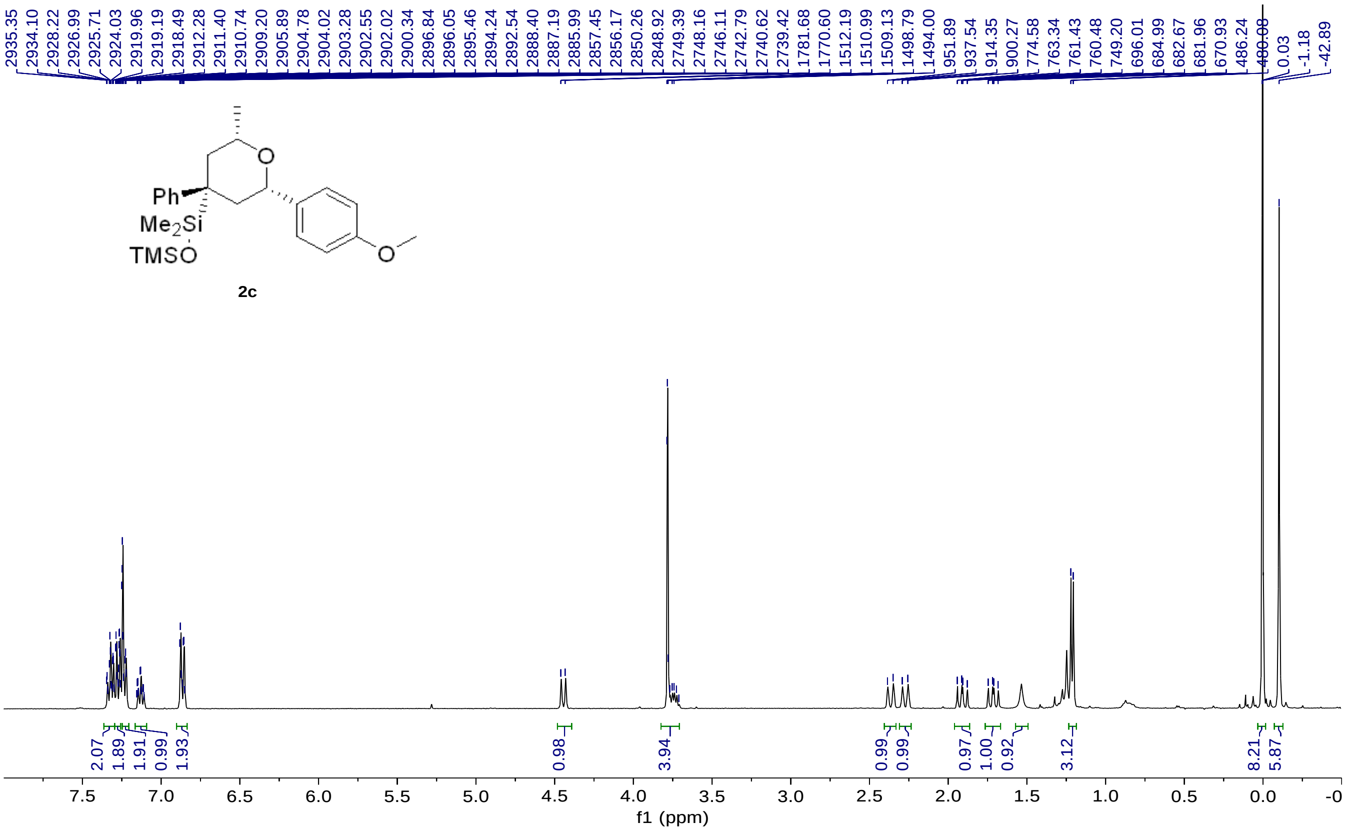

^{13}C NMR (101 MHz, CDCl3)

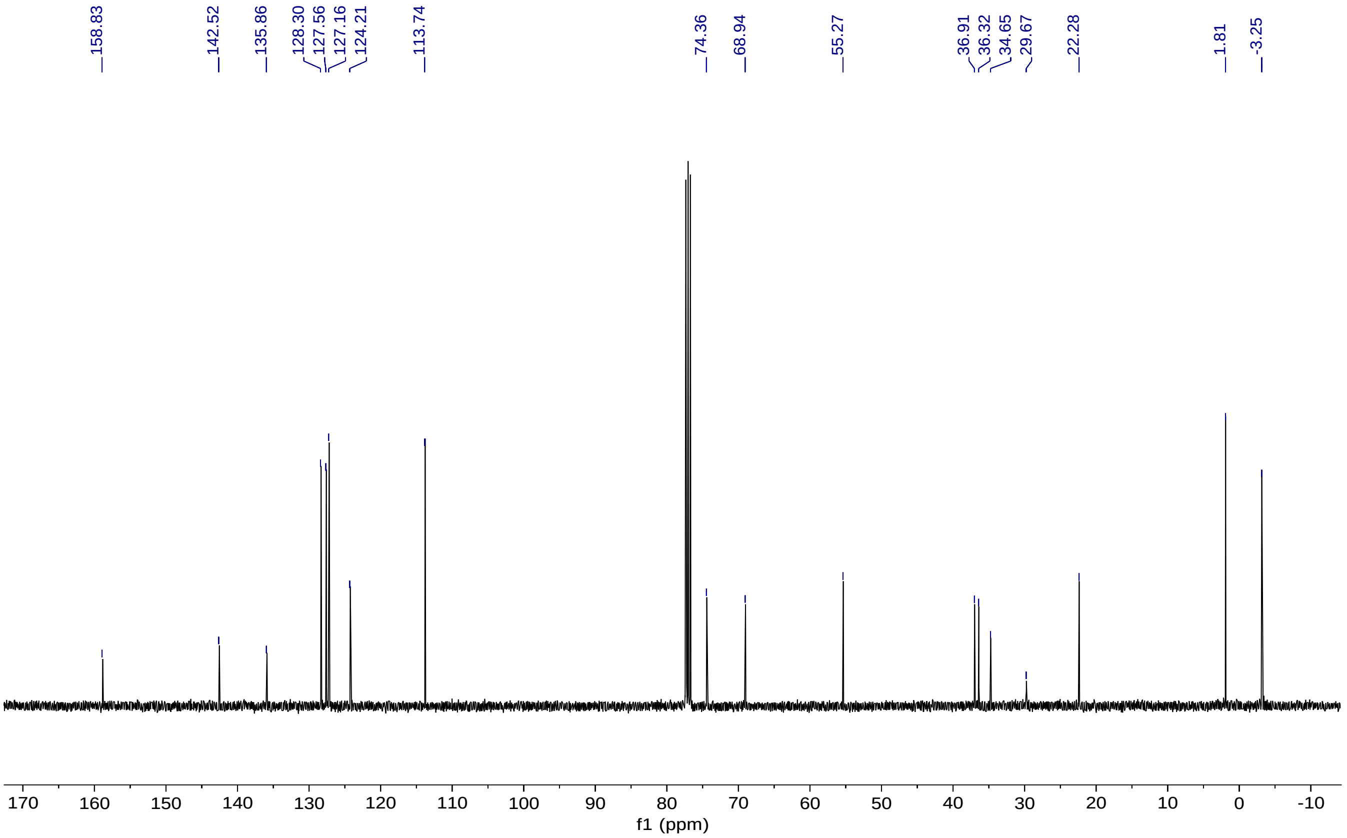

^{1}H NMR (400 MHz, CDCl₃)

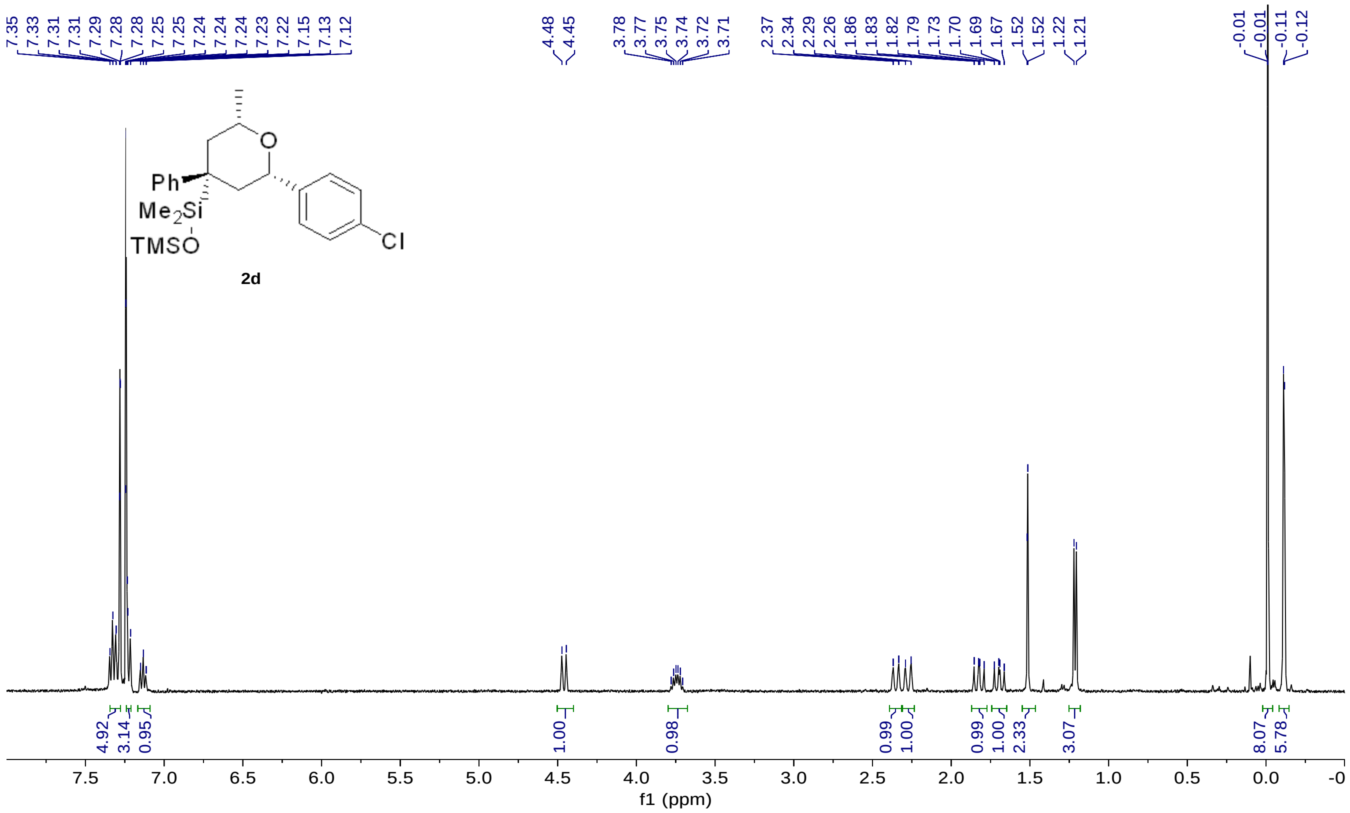

^{13}C NMR (101 MHz, CDCl₃)

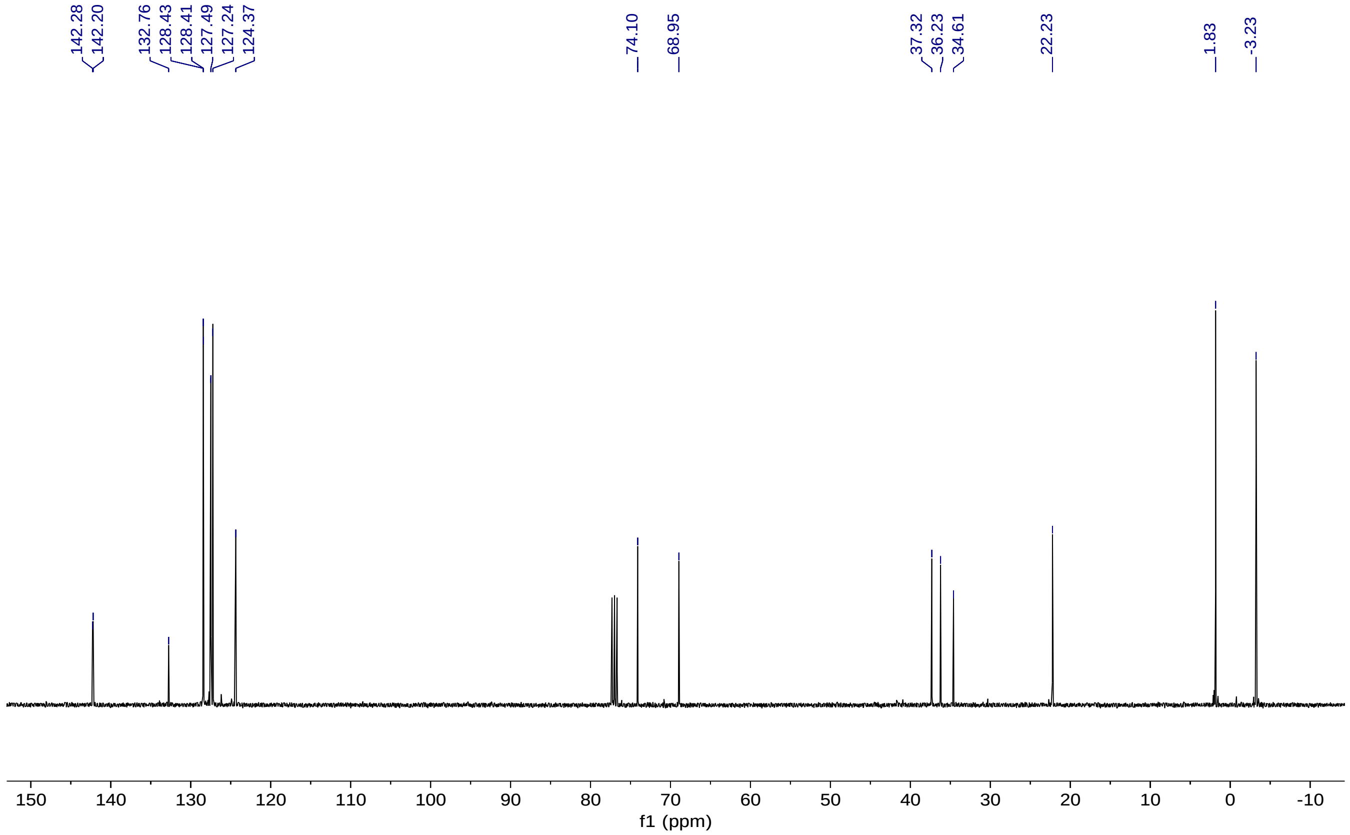

^{1}H NMR (400 MHz, CDCl3)

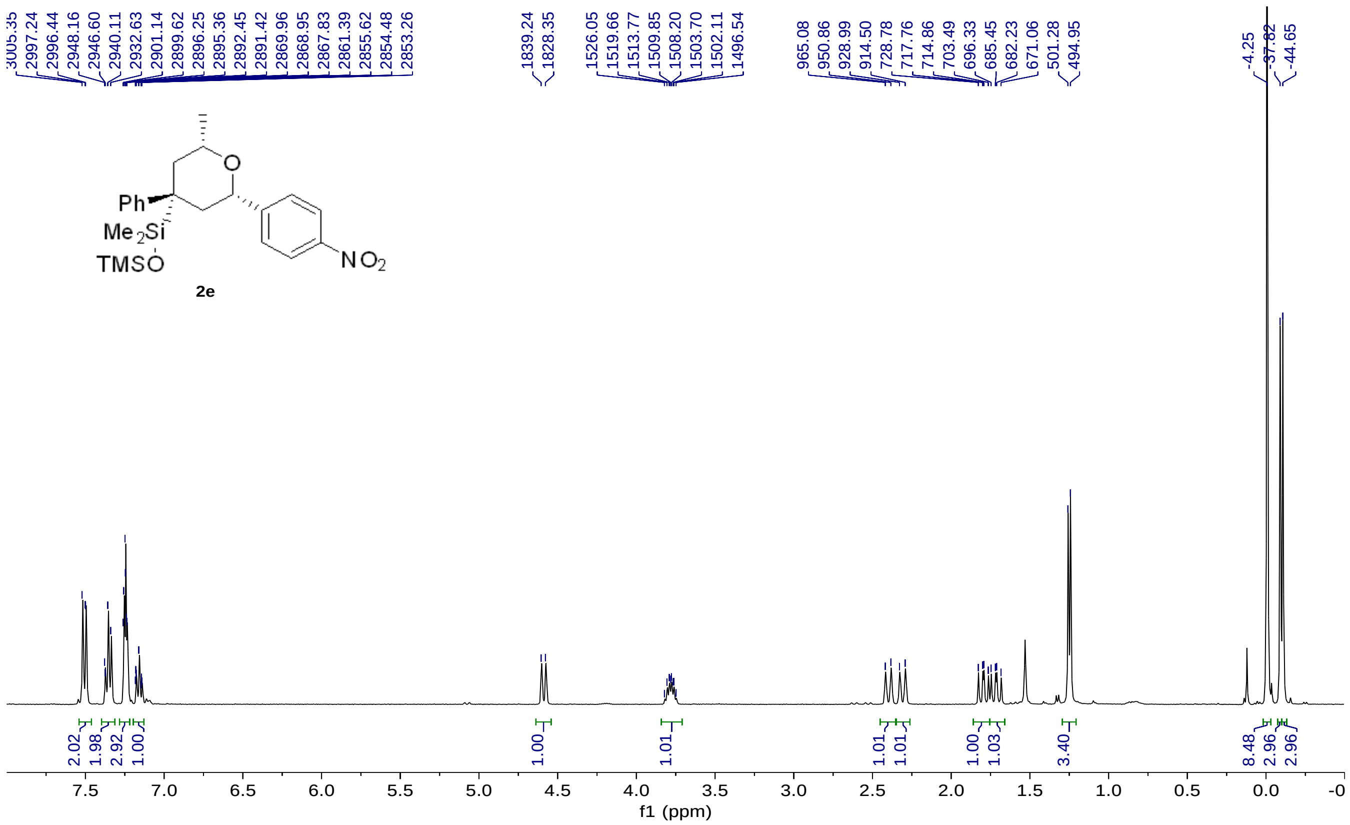

^{13}C NMR (101 MHz, CDCl3)

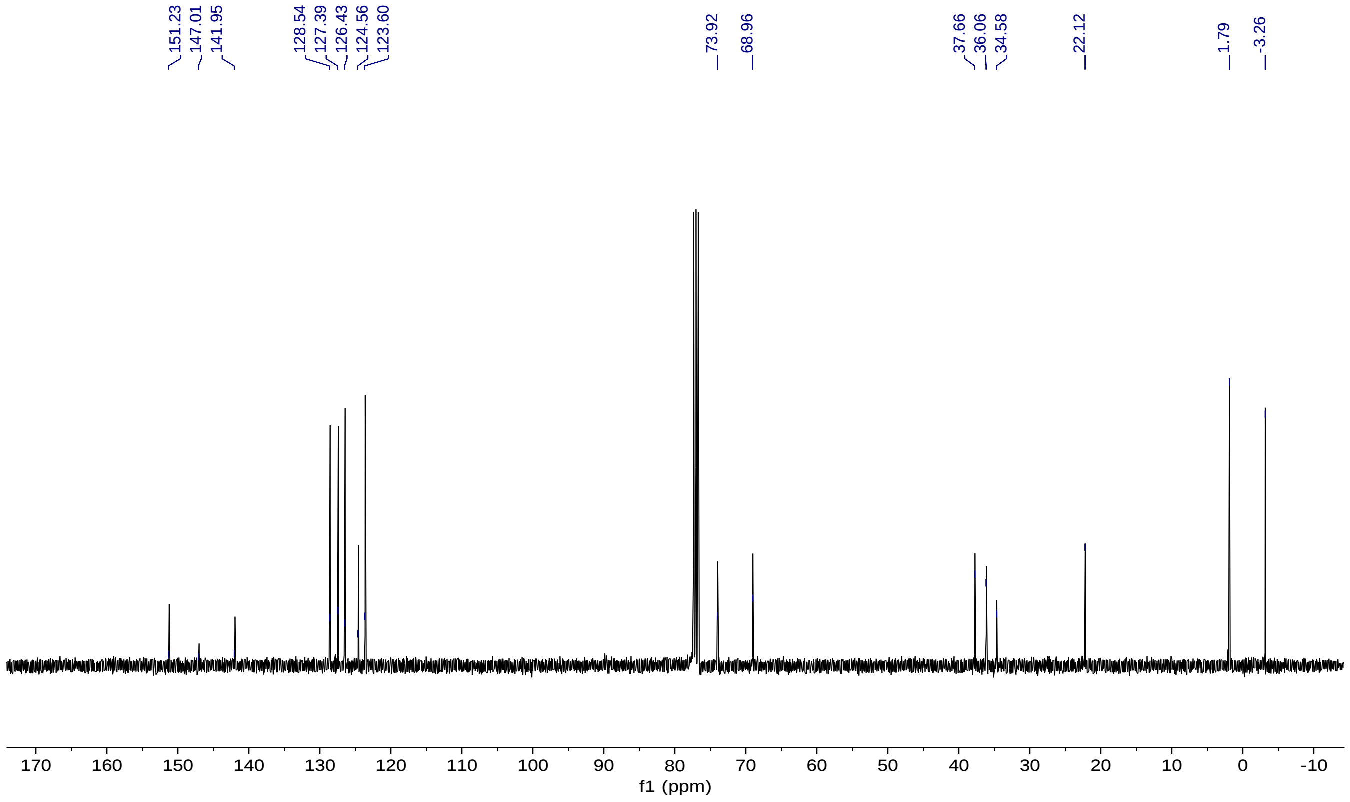

^{1}H NMR (400 MHz, $CDCl_3$)

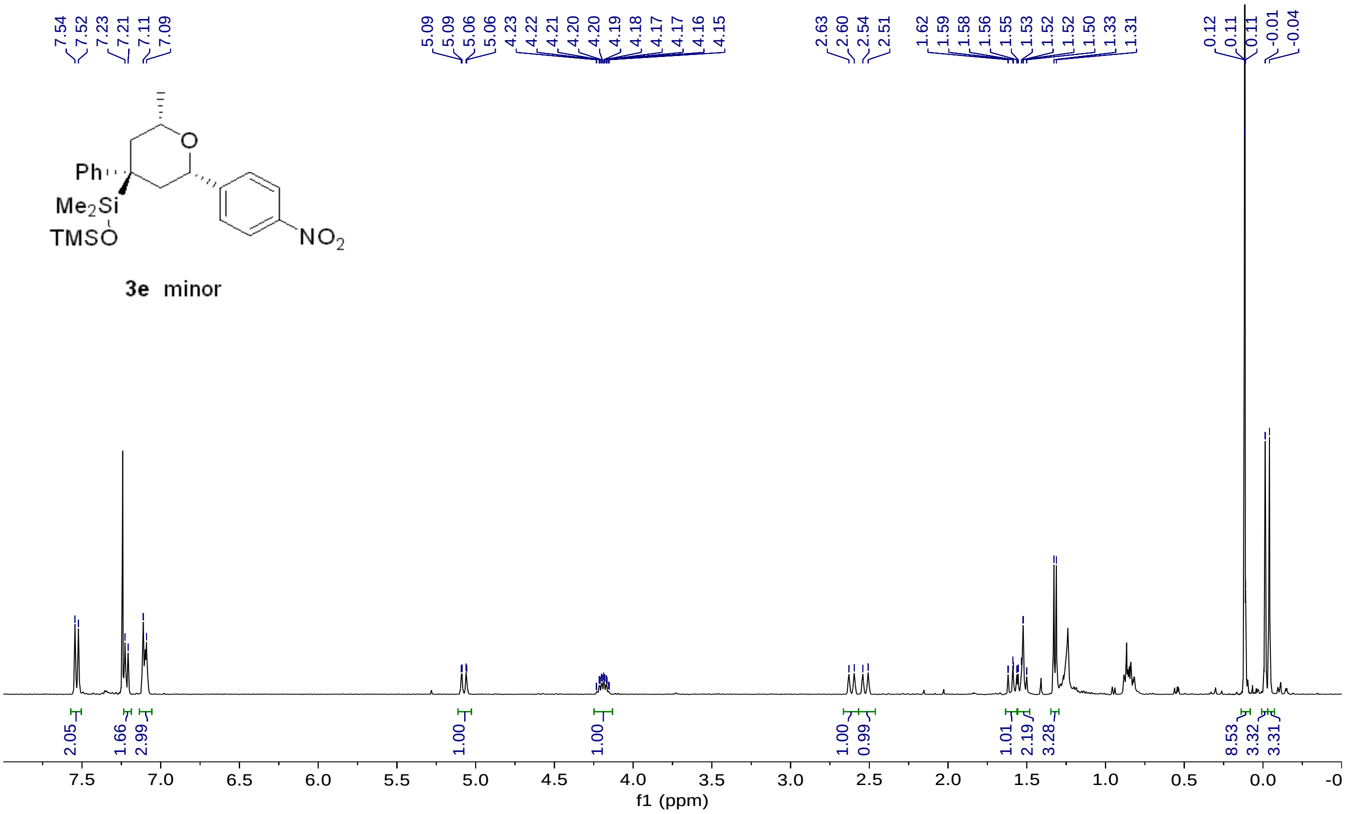

^{13}C NMR (101 MHz, $CDCl_3$)

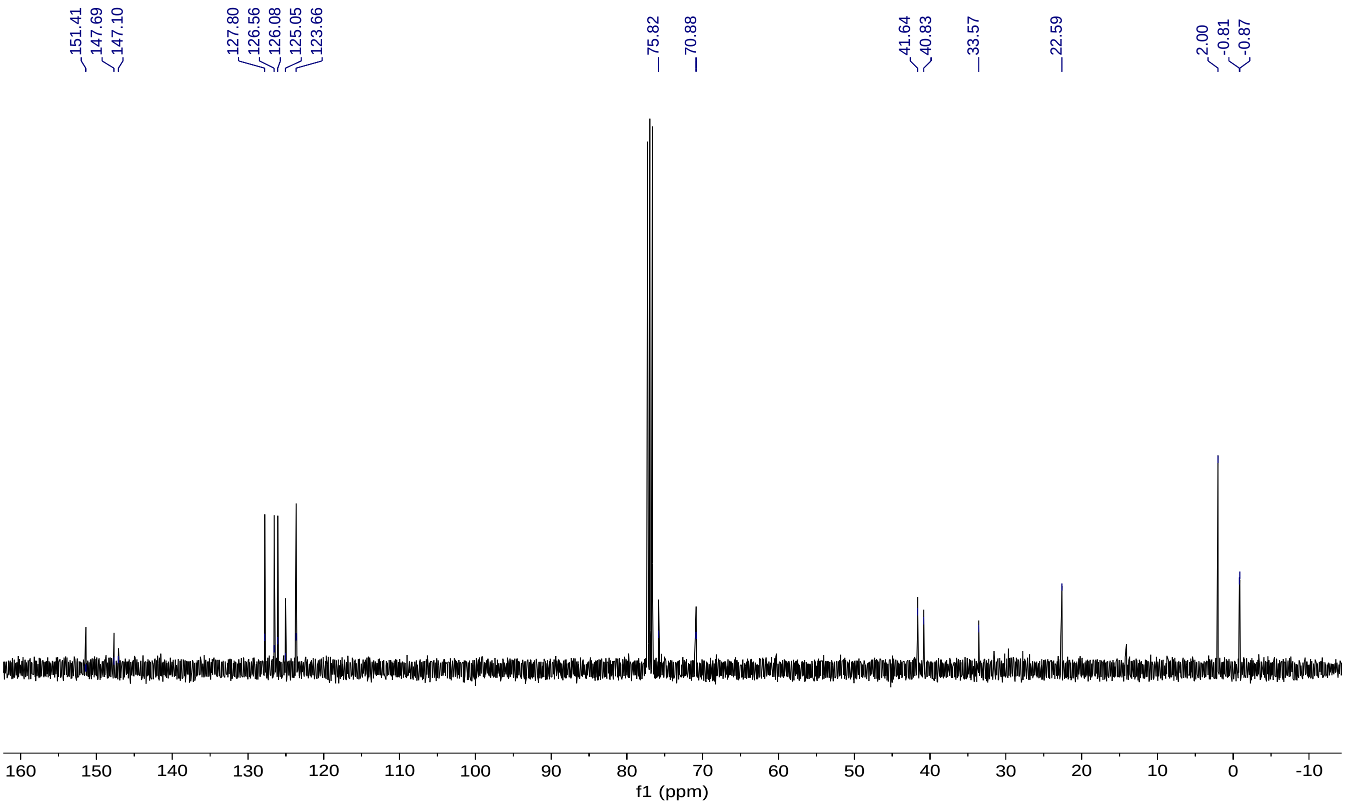

^{1}H NMR (500 MHz, $CDCl_3$)

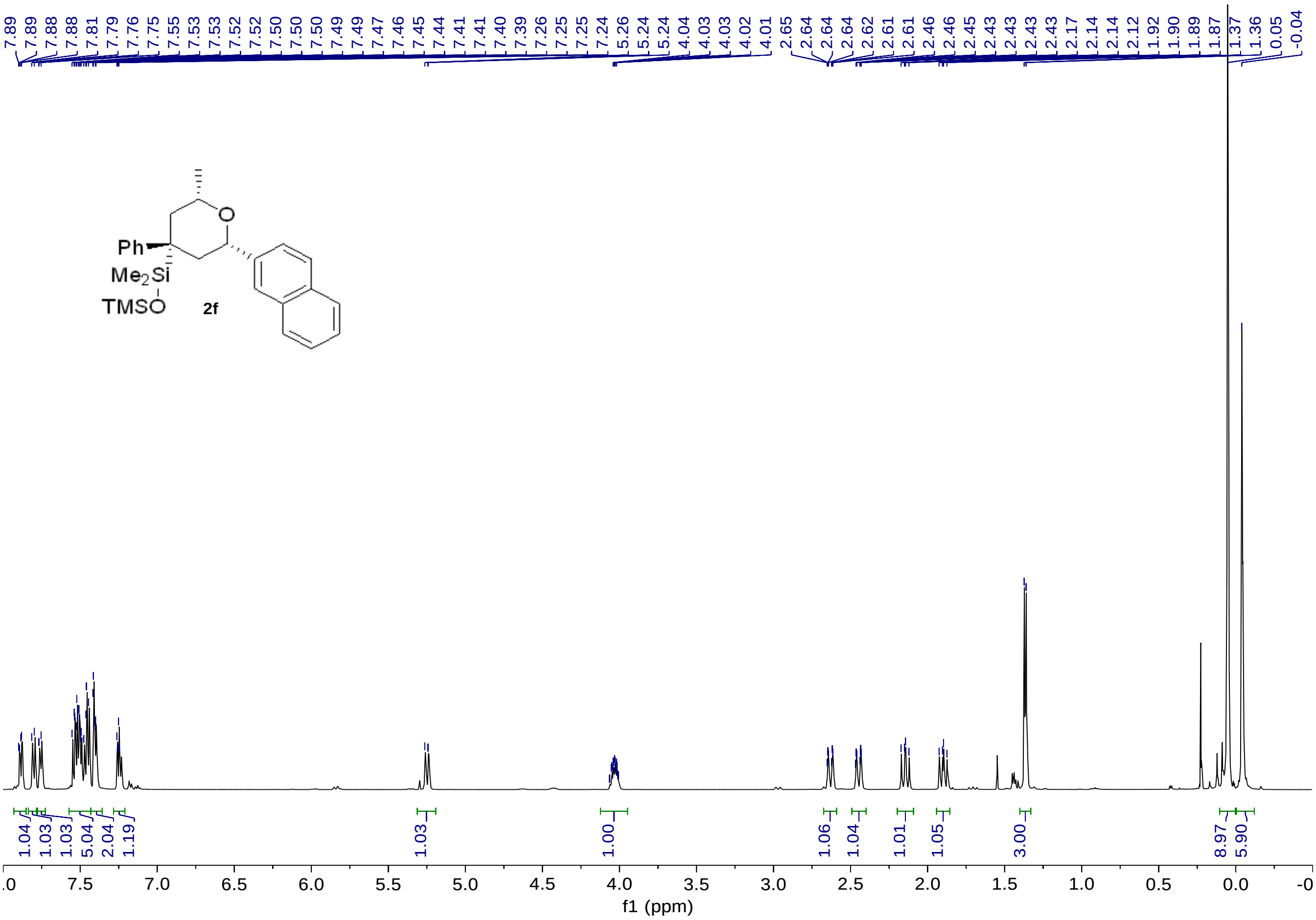

^{13}C NMR (101 MHz, $CDCl_3$)

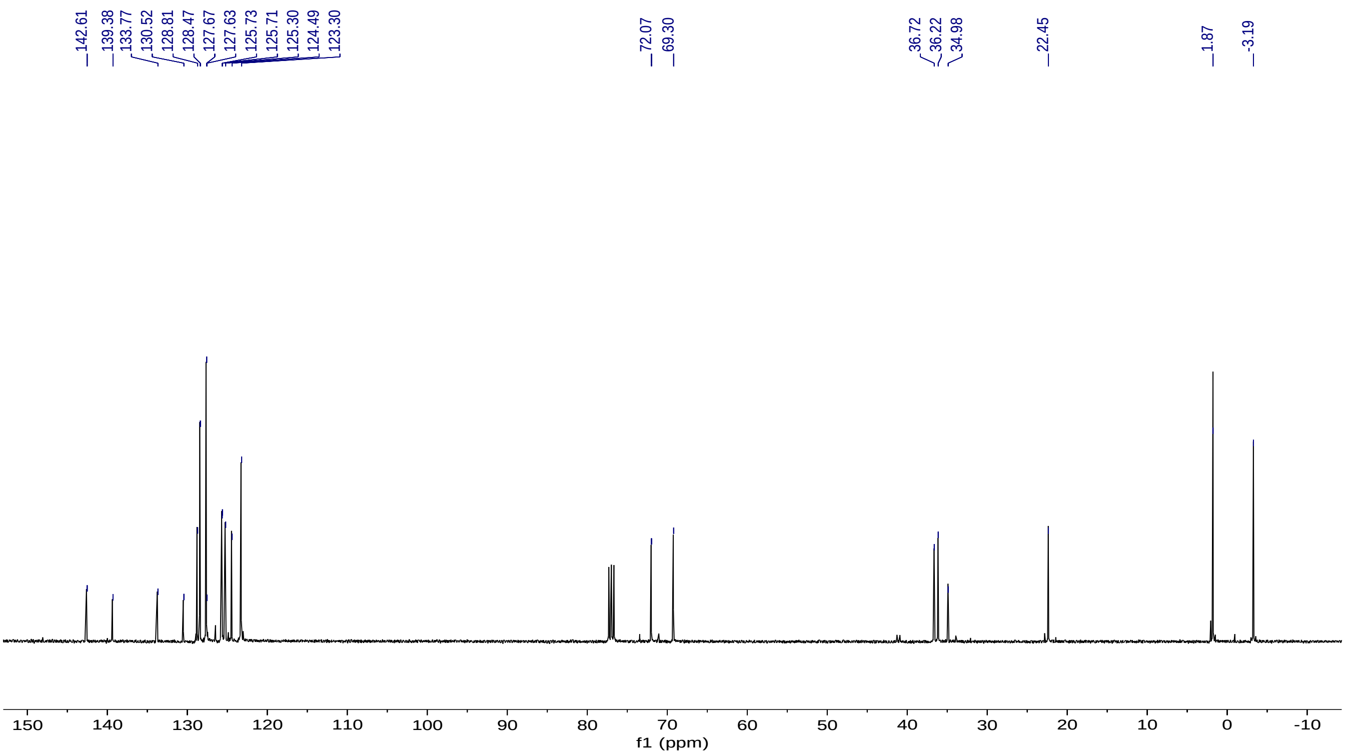

^{1}H NMR (500 MHz, $CDCl_3$)

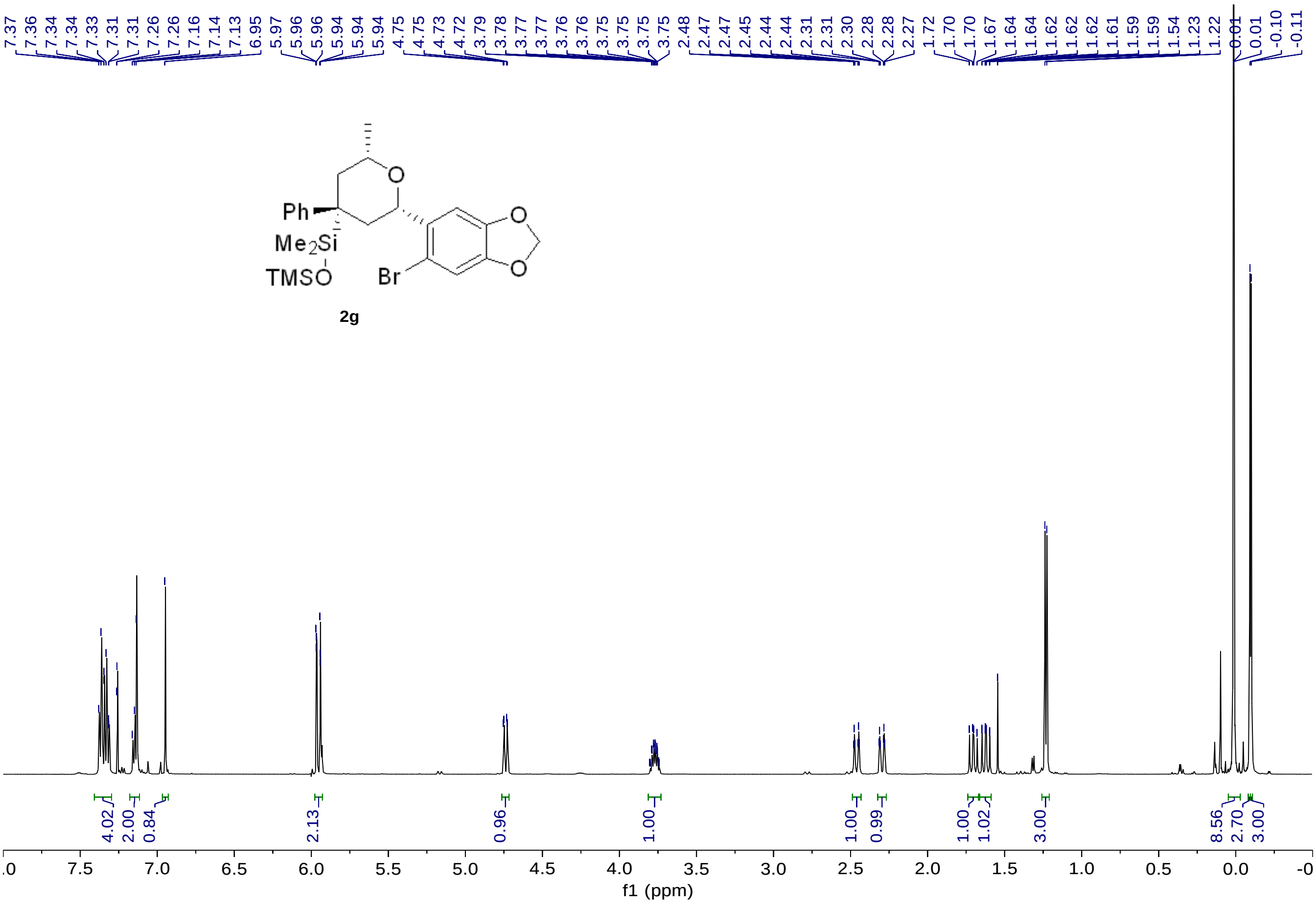

^{13}C NMR (101 MHz, $CDCl_3$)

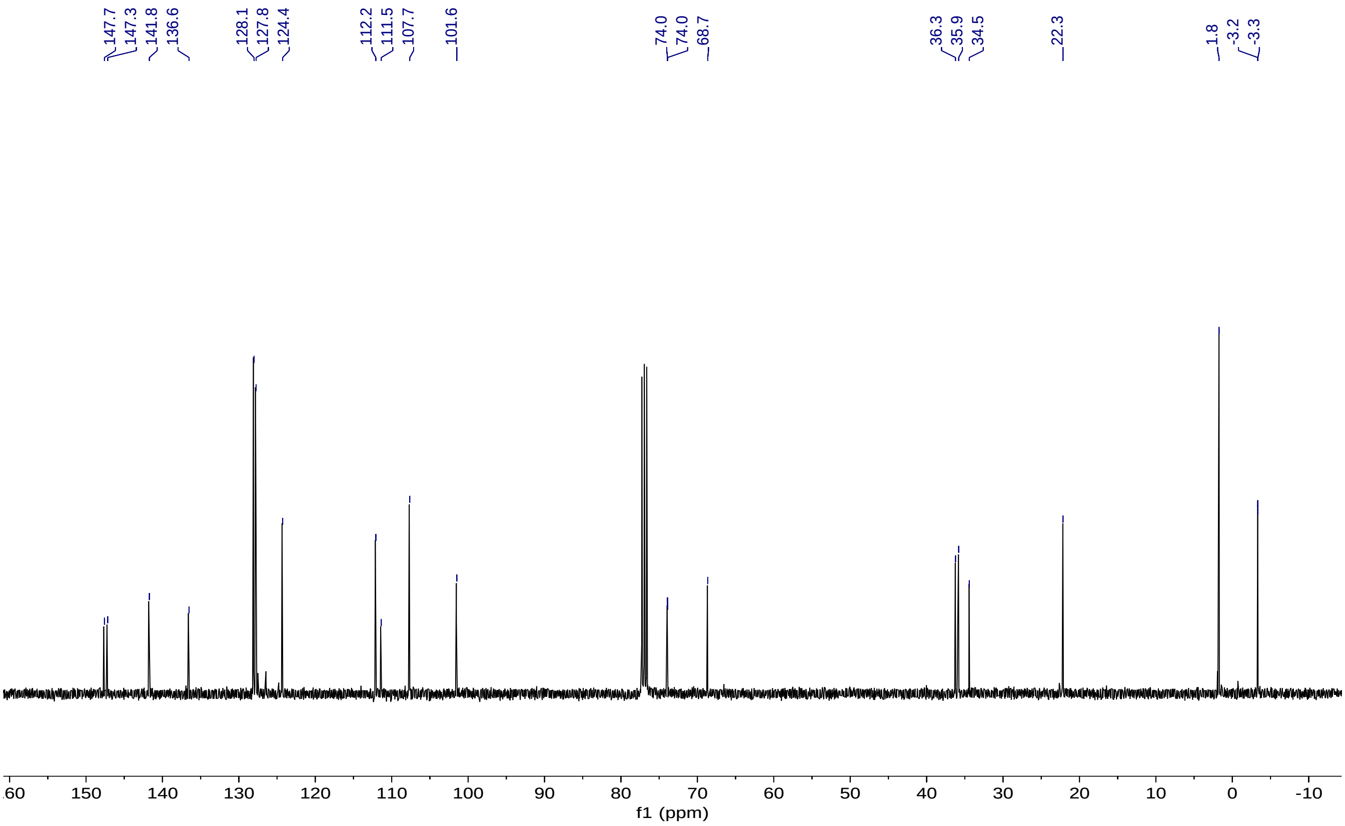

^{1}H NMR (400 MHz, $CDCl_3$)

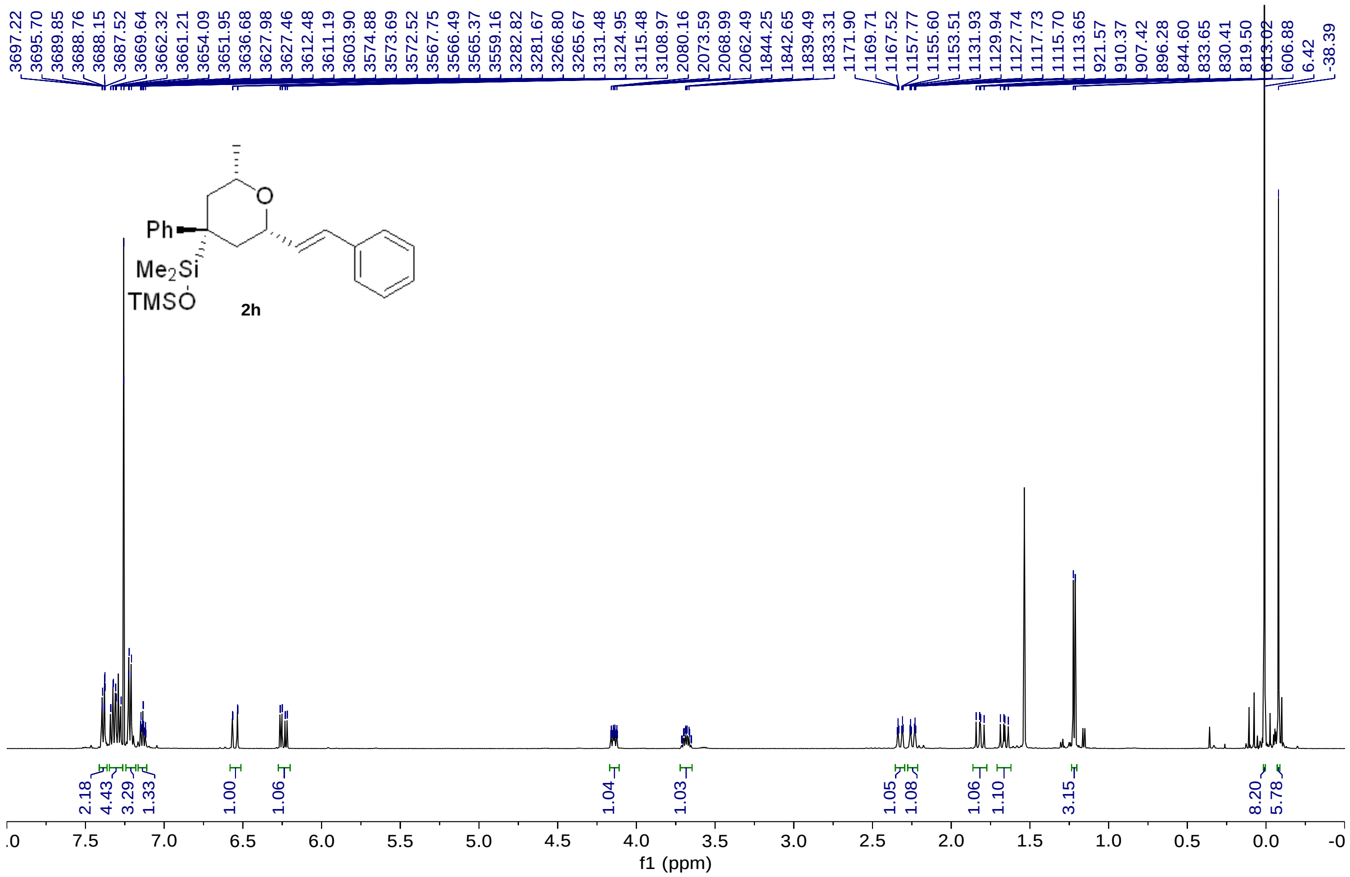

^{13}C NMR (101 MHz, $CDCl_3$)

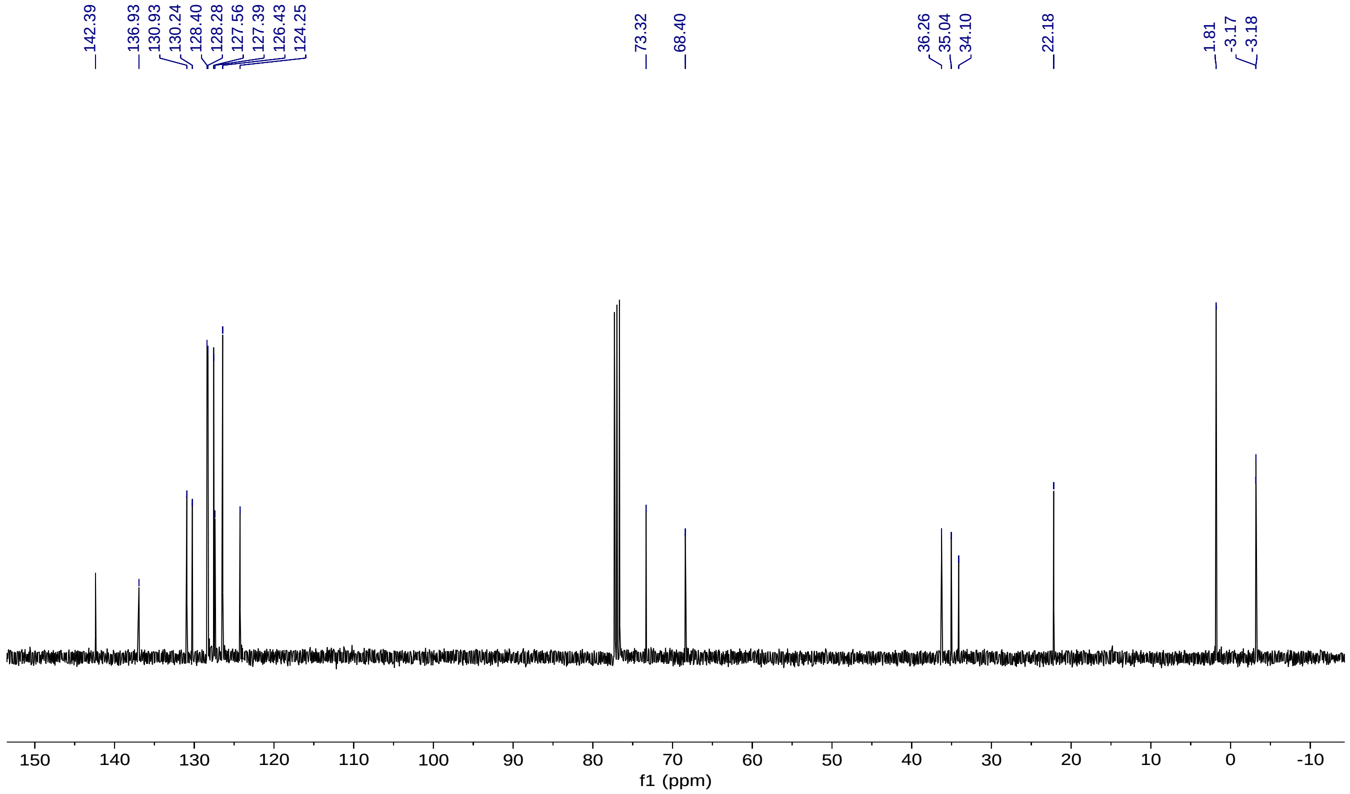

^{1}H NMR (400 MHz, $CDCl_3$)

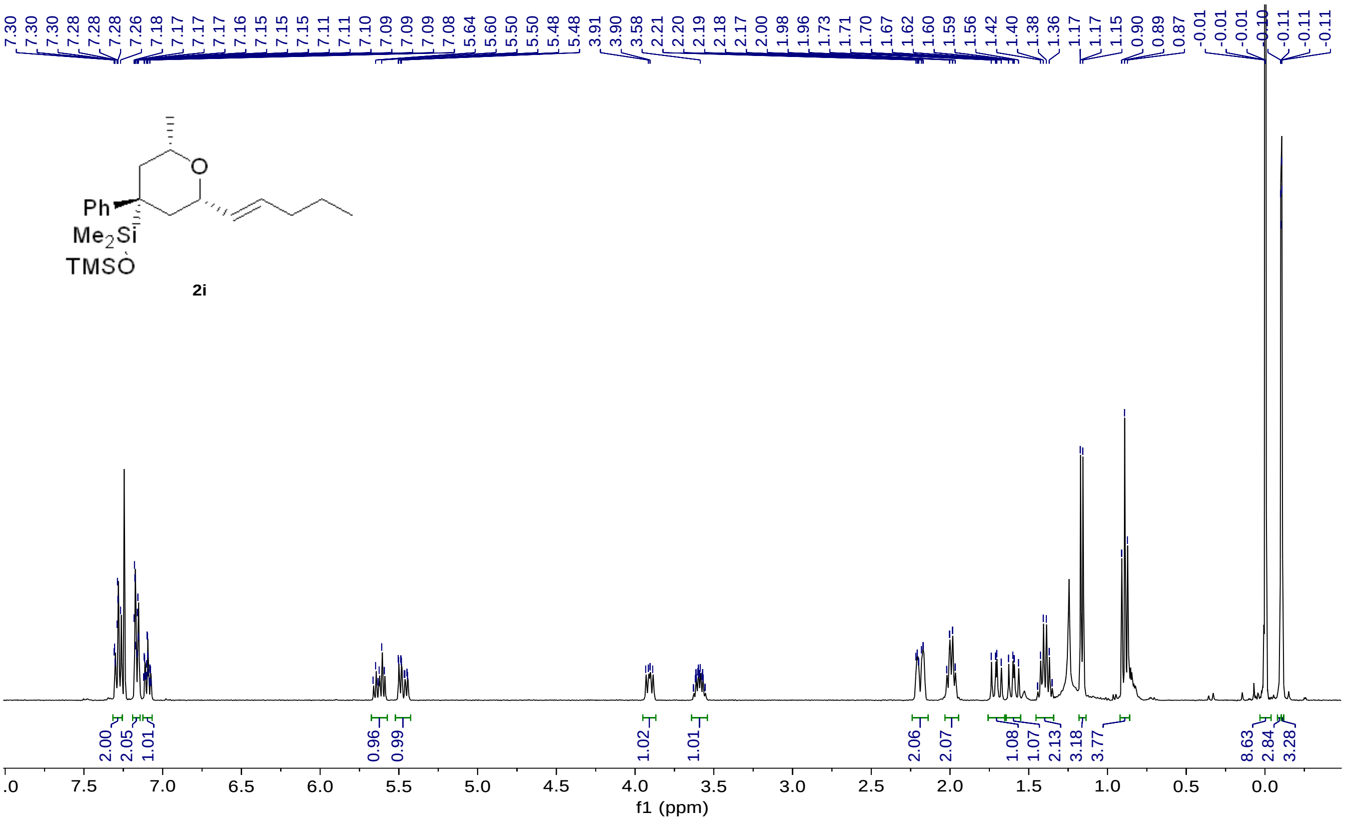

^{13}C NMR (101 MHz, $CDCl_3$)

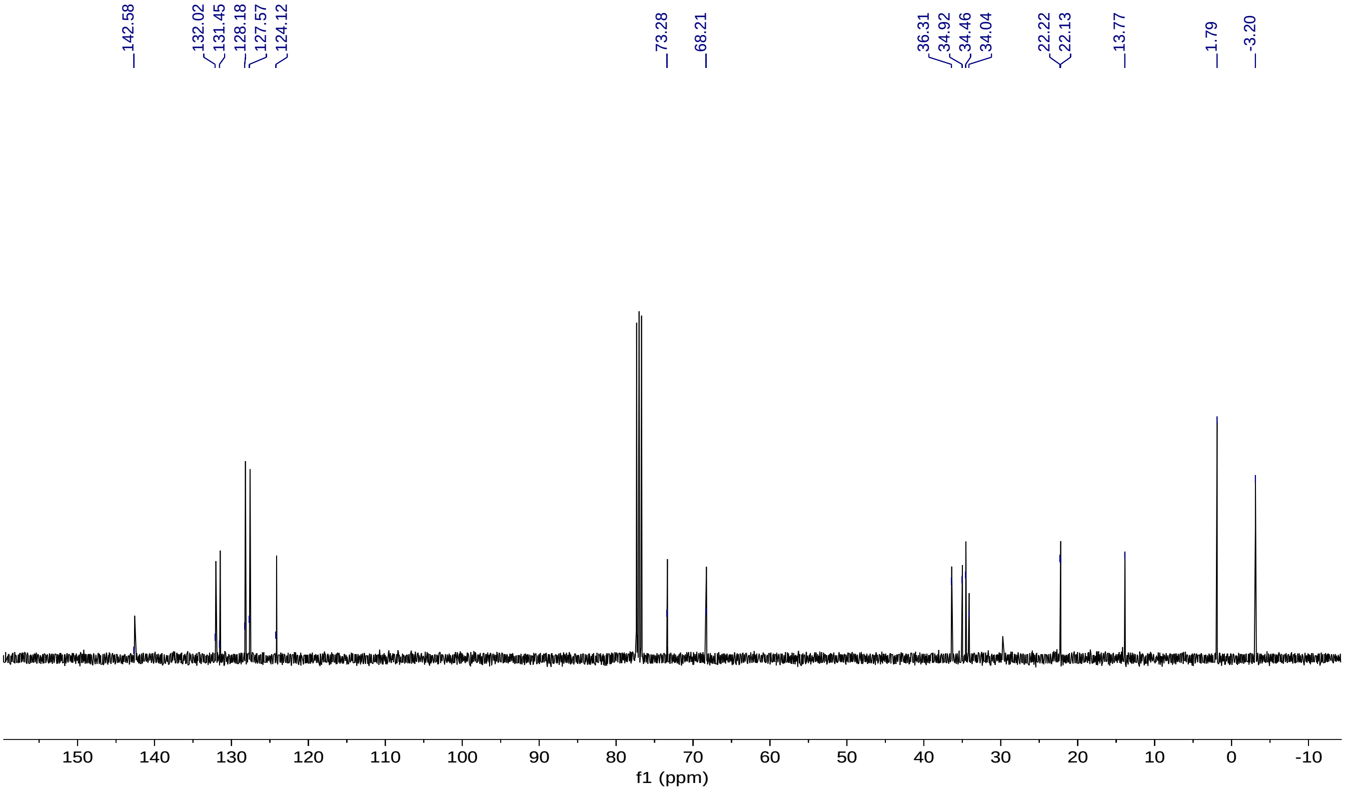

^{1}H NMR (400 MHz, $CDCl_3$)

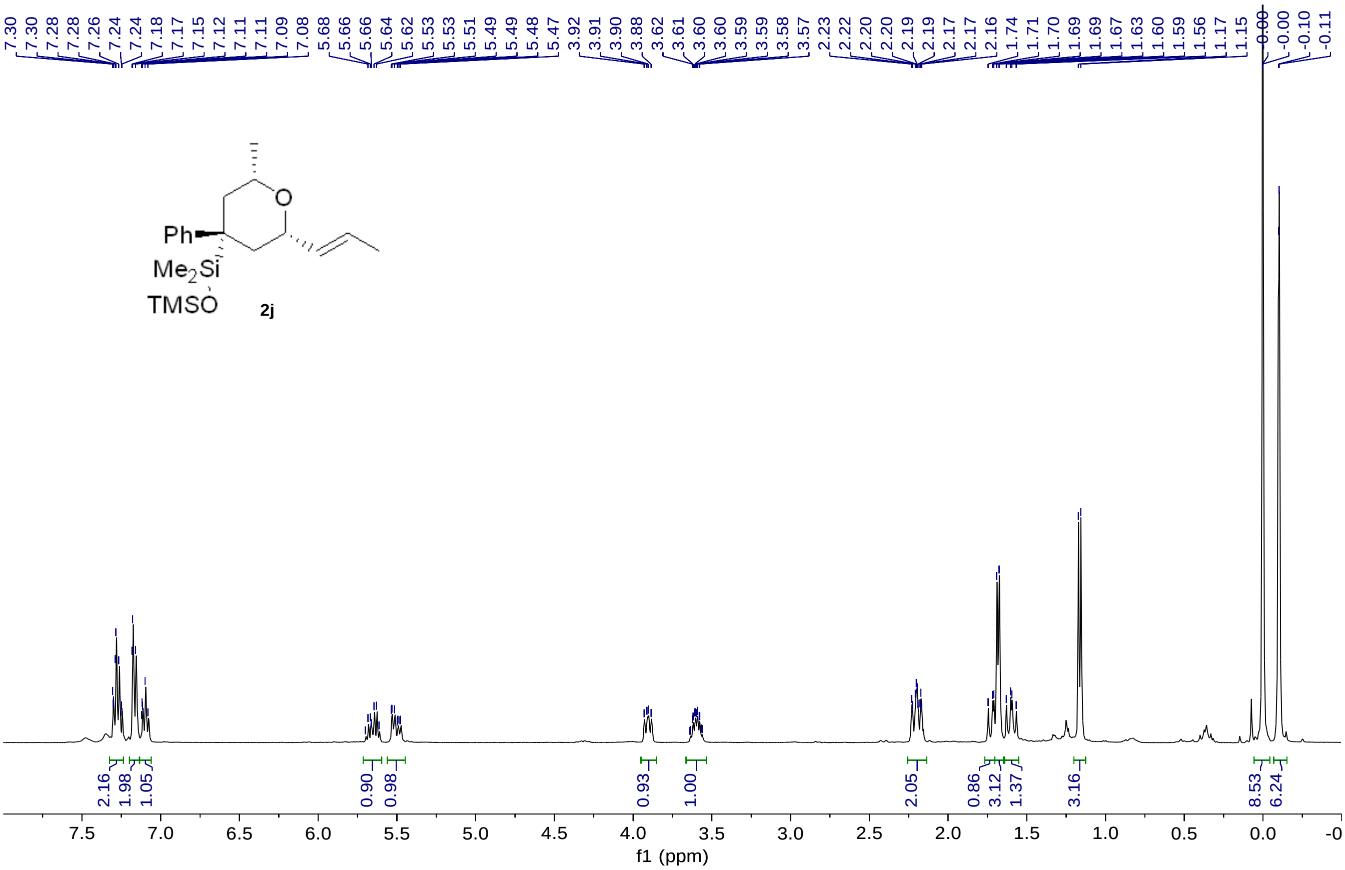

^{13}C NMR (101 MHz, $CDCl_3$)

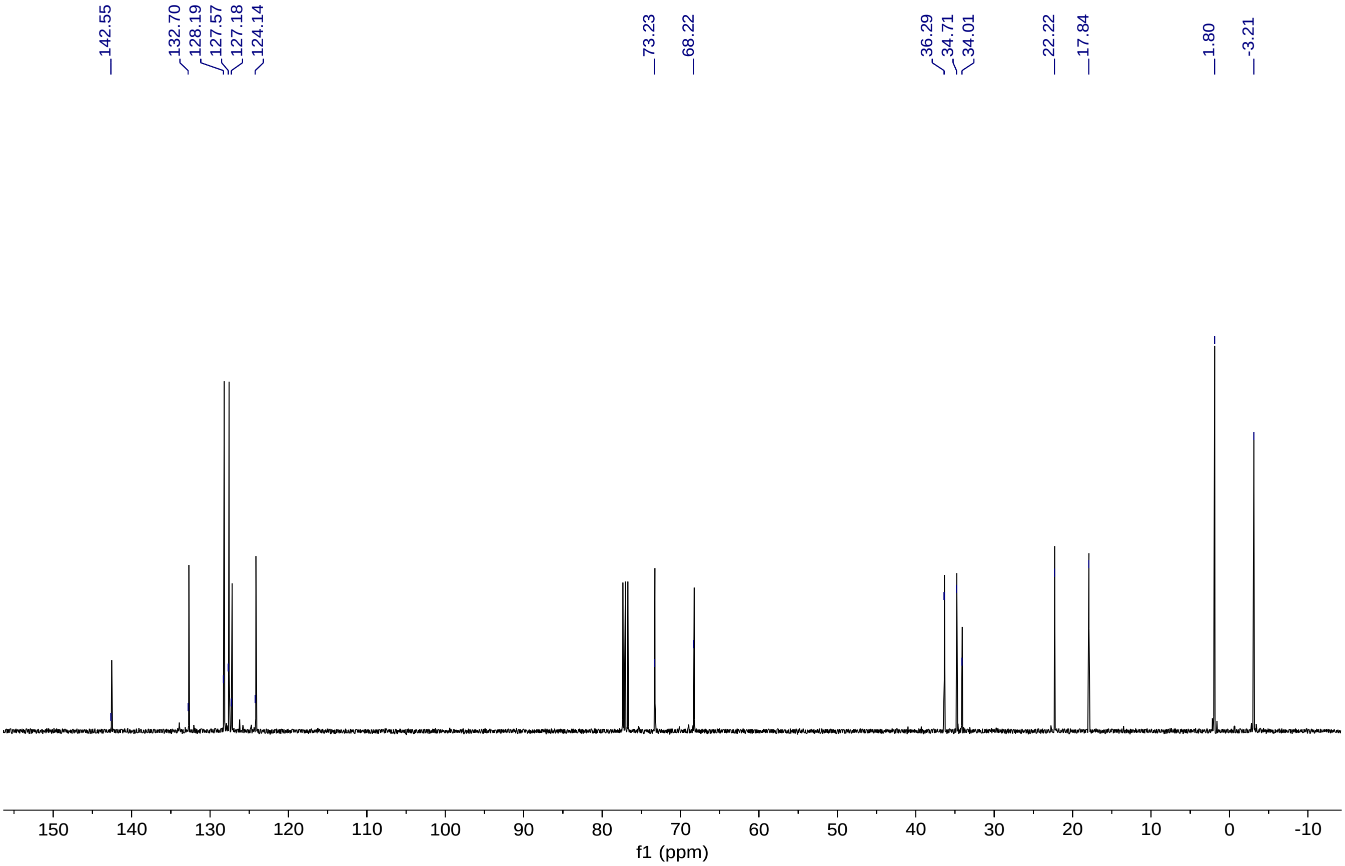

^{1}H NMR (400 MHz, $CDCl_3$)

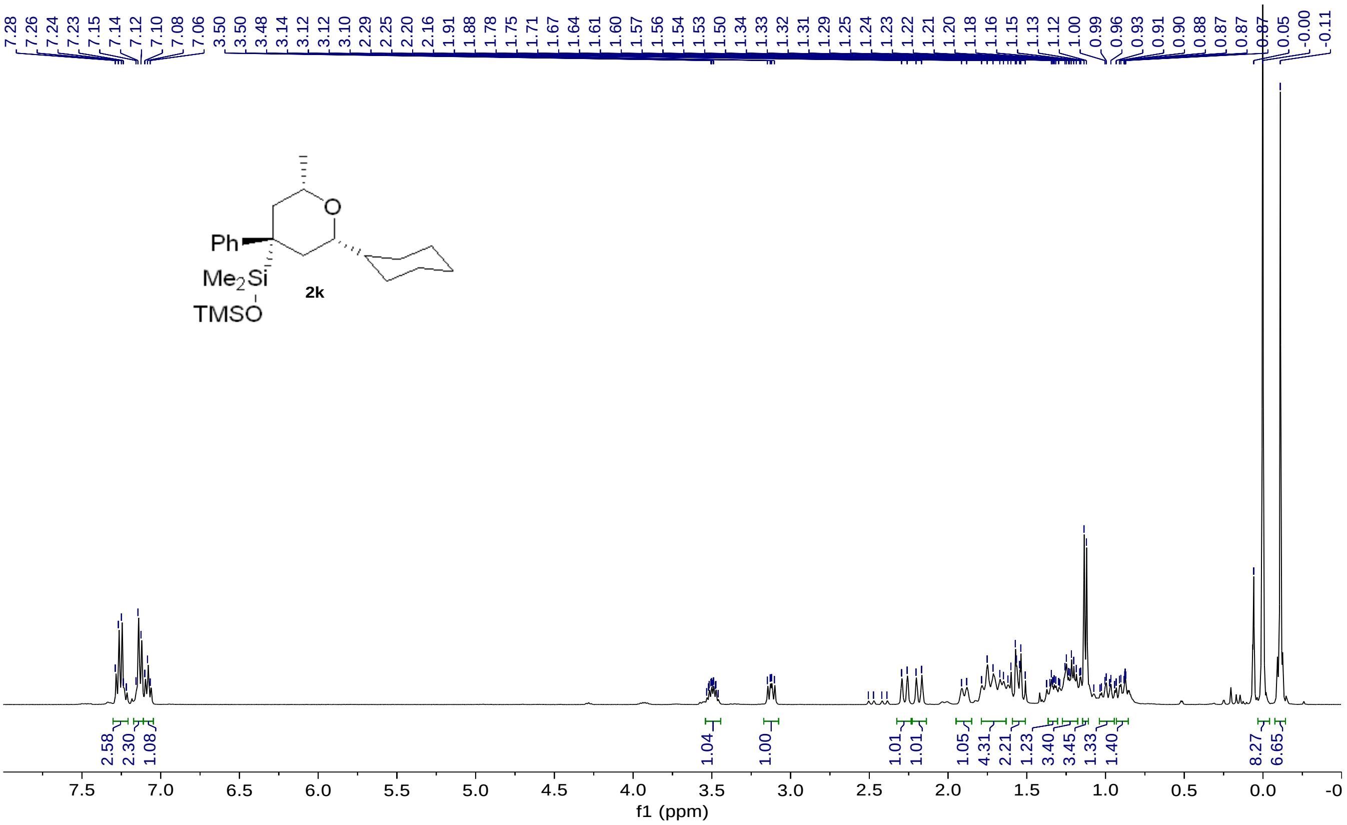

^{13}C NMR (101 MHz, $CDCl_3$)

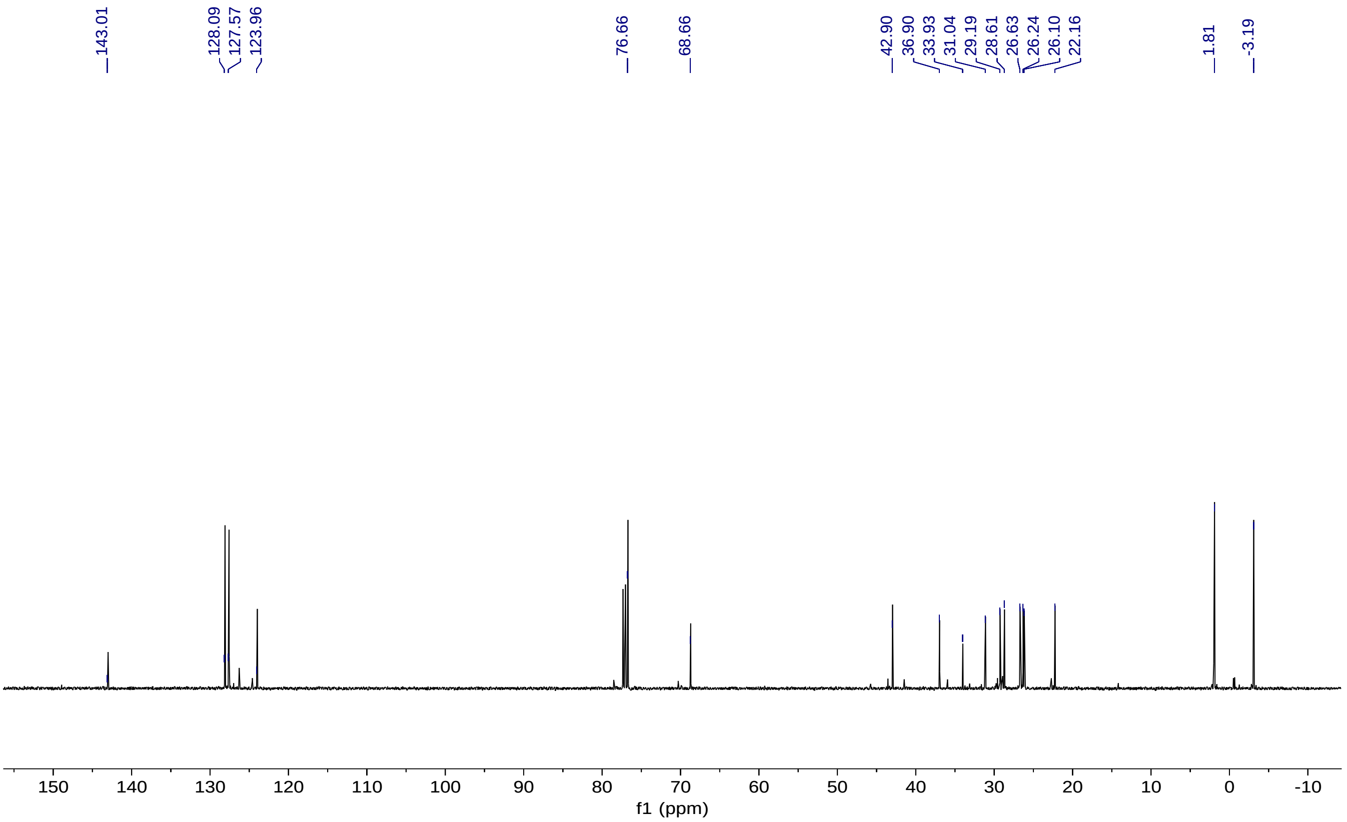

^{1}H NMR (400 MHz, $CDCl_3$)

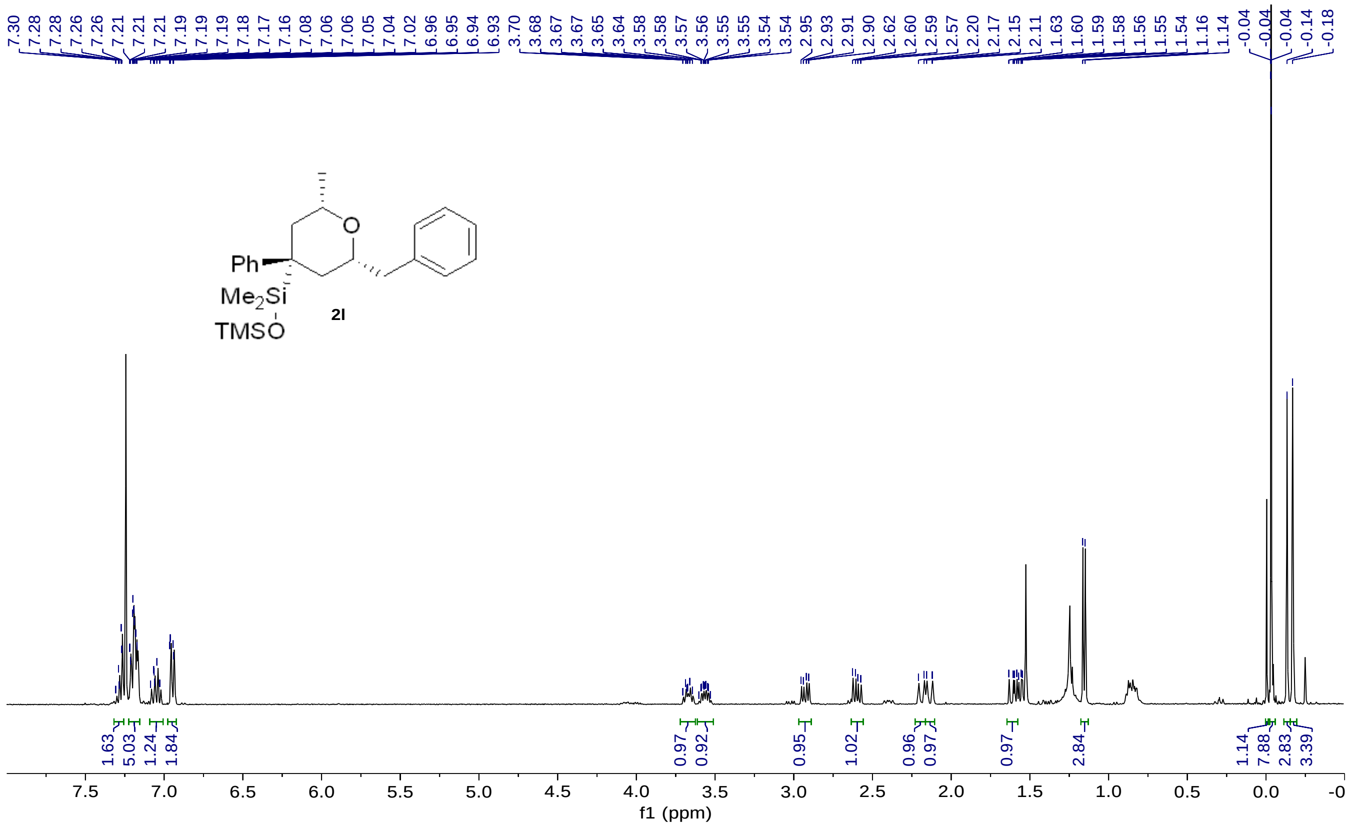

^{13}C NMR (101 MHz, $CDCl_3$)

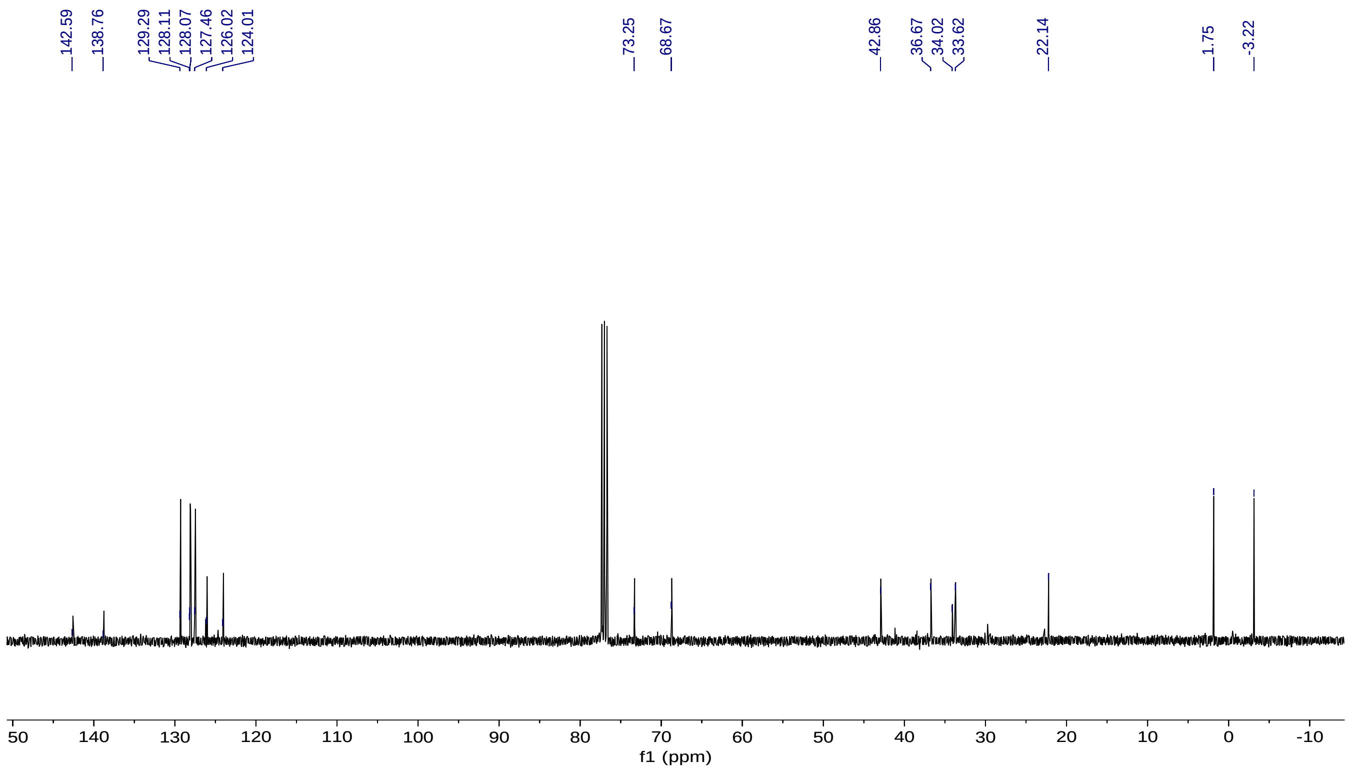

^{1}H NMR (400 MHz, CDCl3)

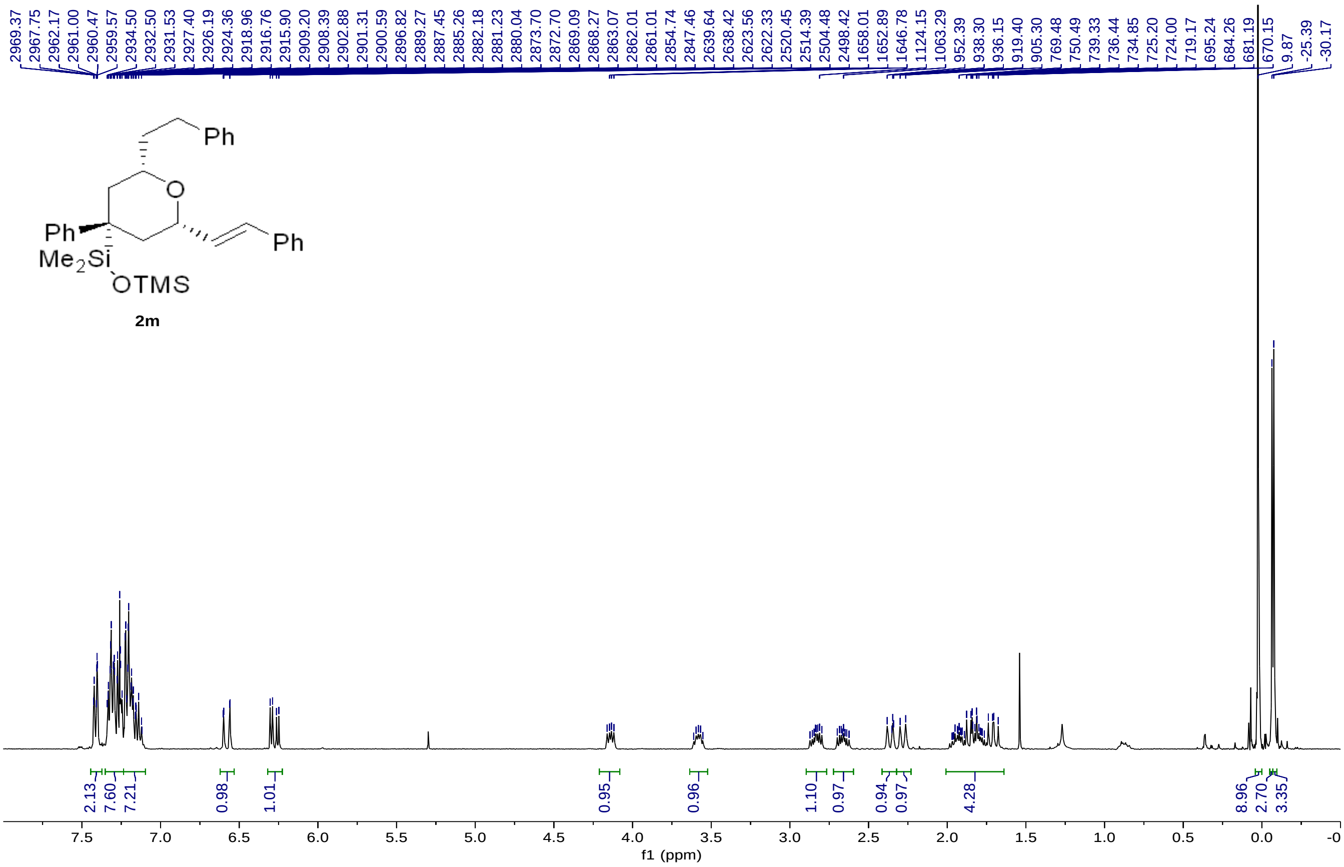

2m

^{13}C NMR (101 MHz, CDCl3)

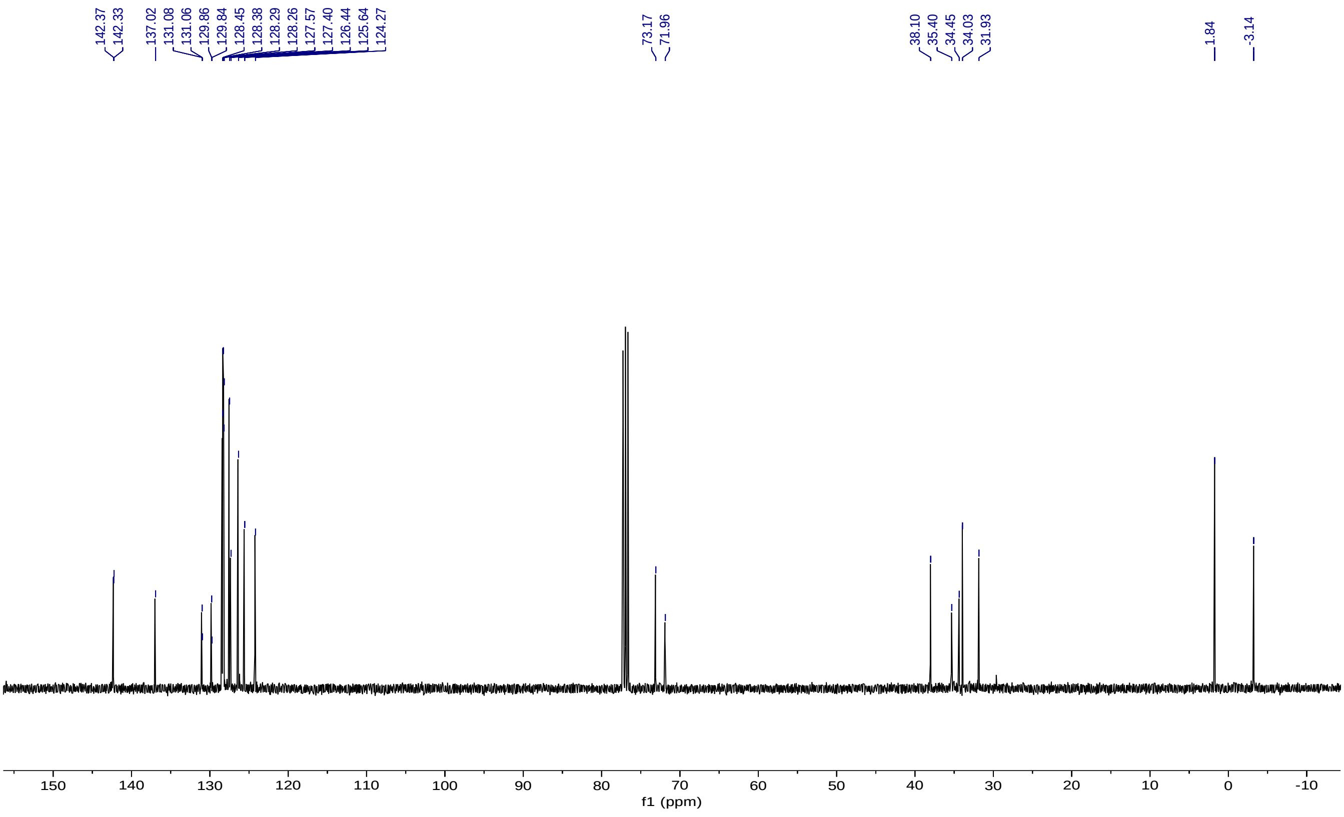

^{1}H NMR (500 MHz, $CDCl_3$)

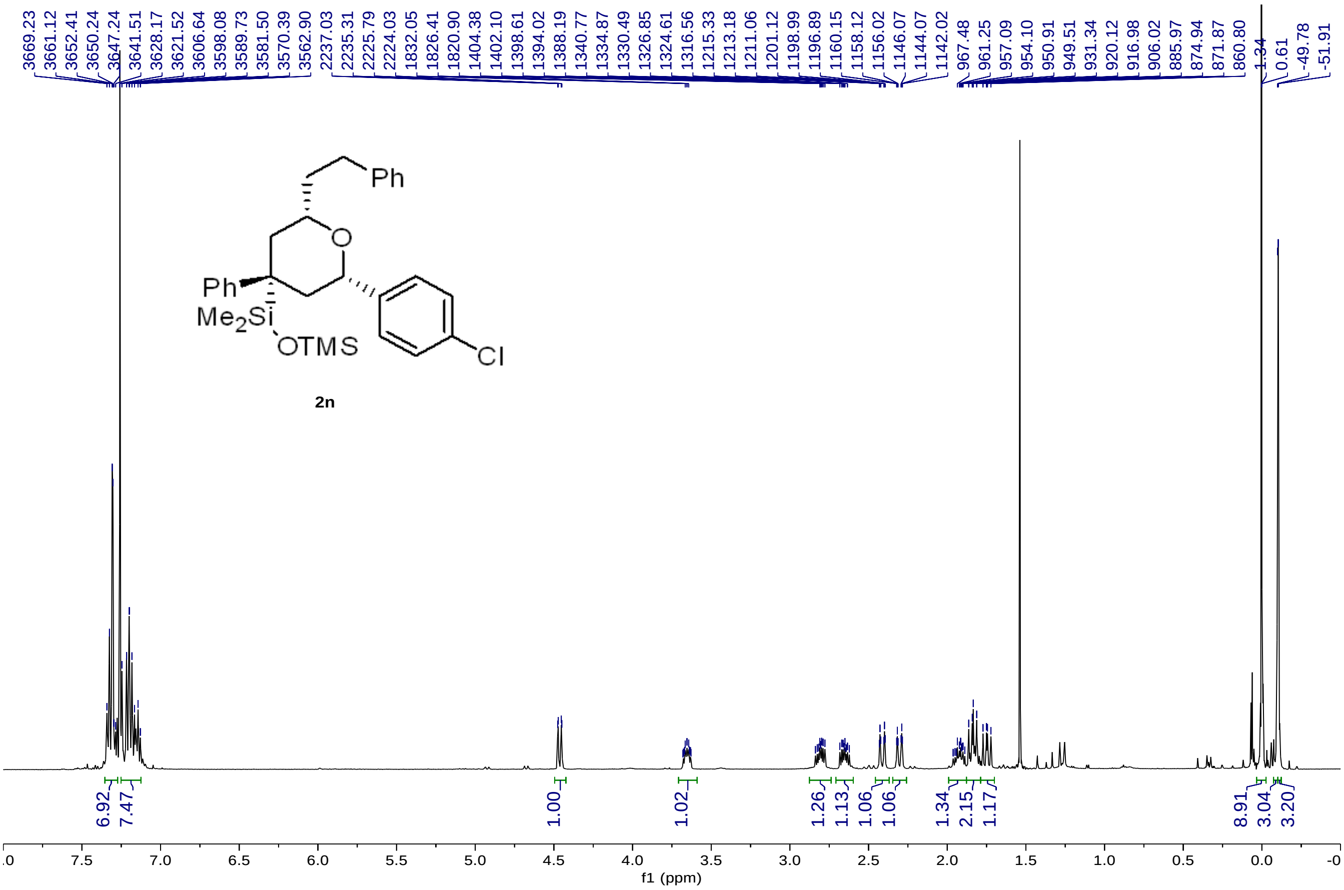

^{13}C NMR (101 MHz, $CDCl_3$)

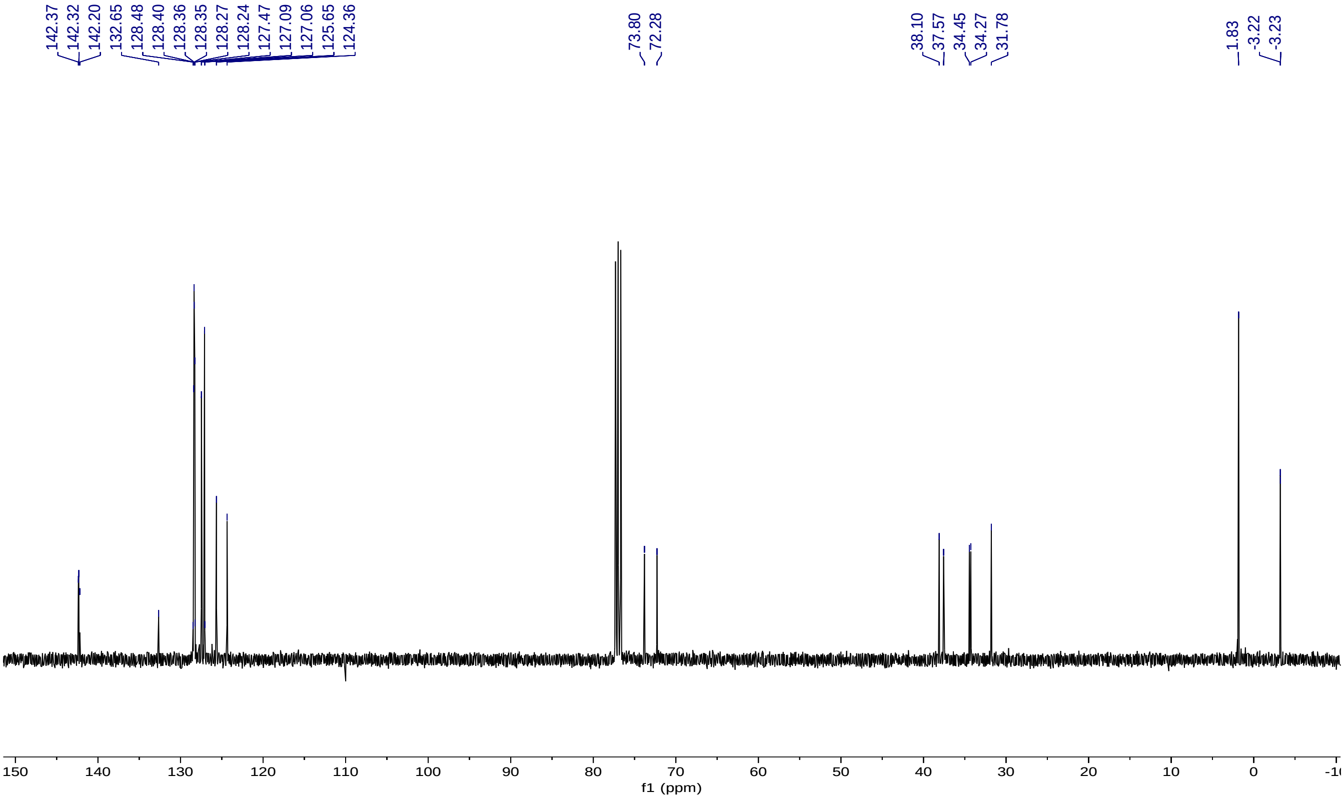

^{1}H NMR (500 MHz, CDCl3)

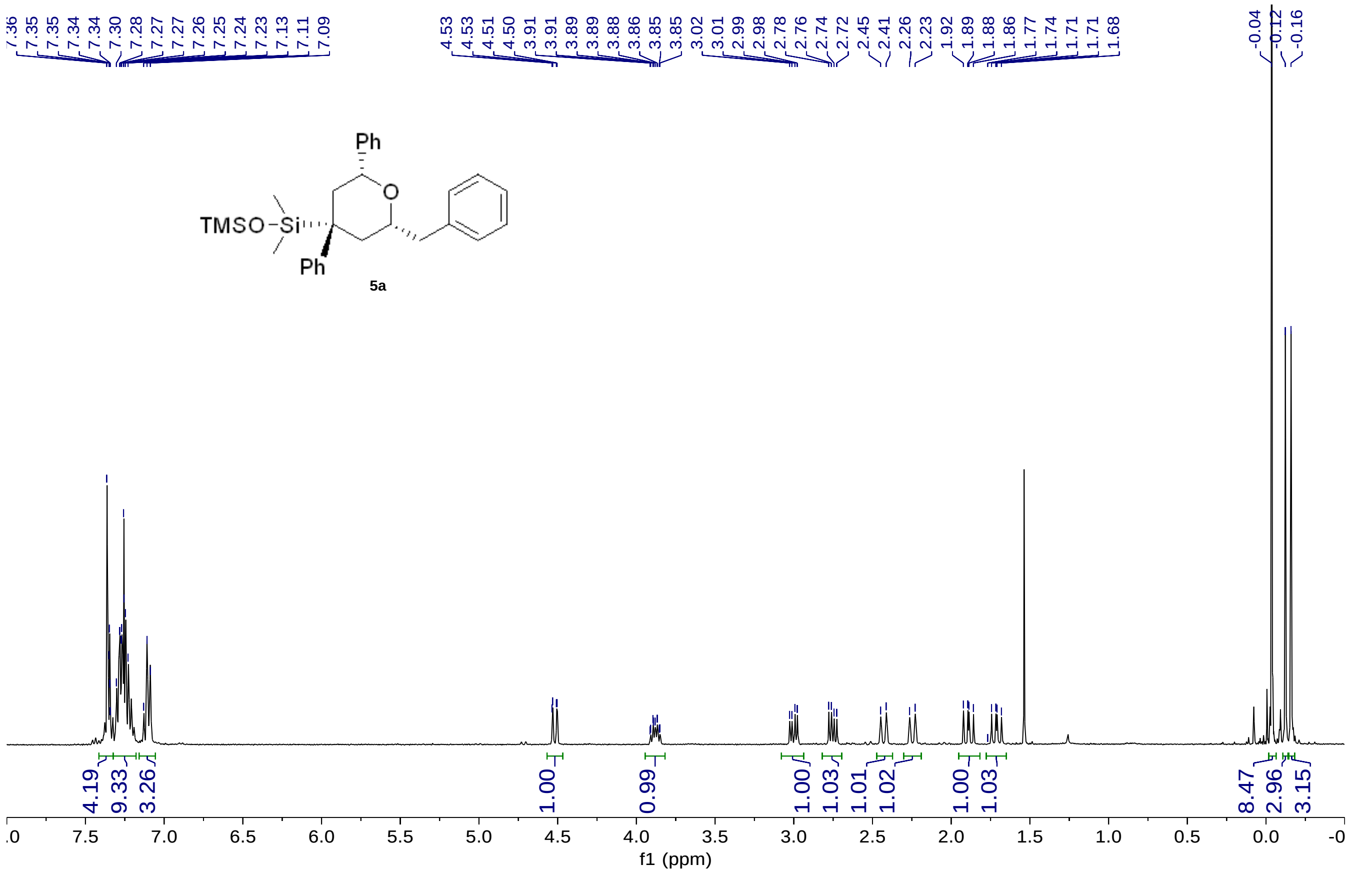

^{13}C NMR (101 MHz, CDCl3)

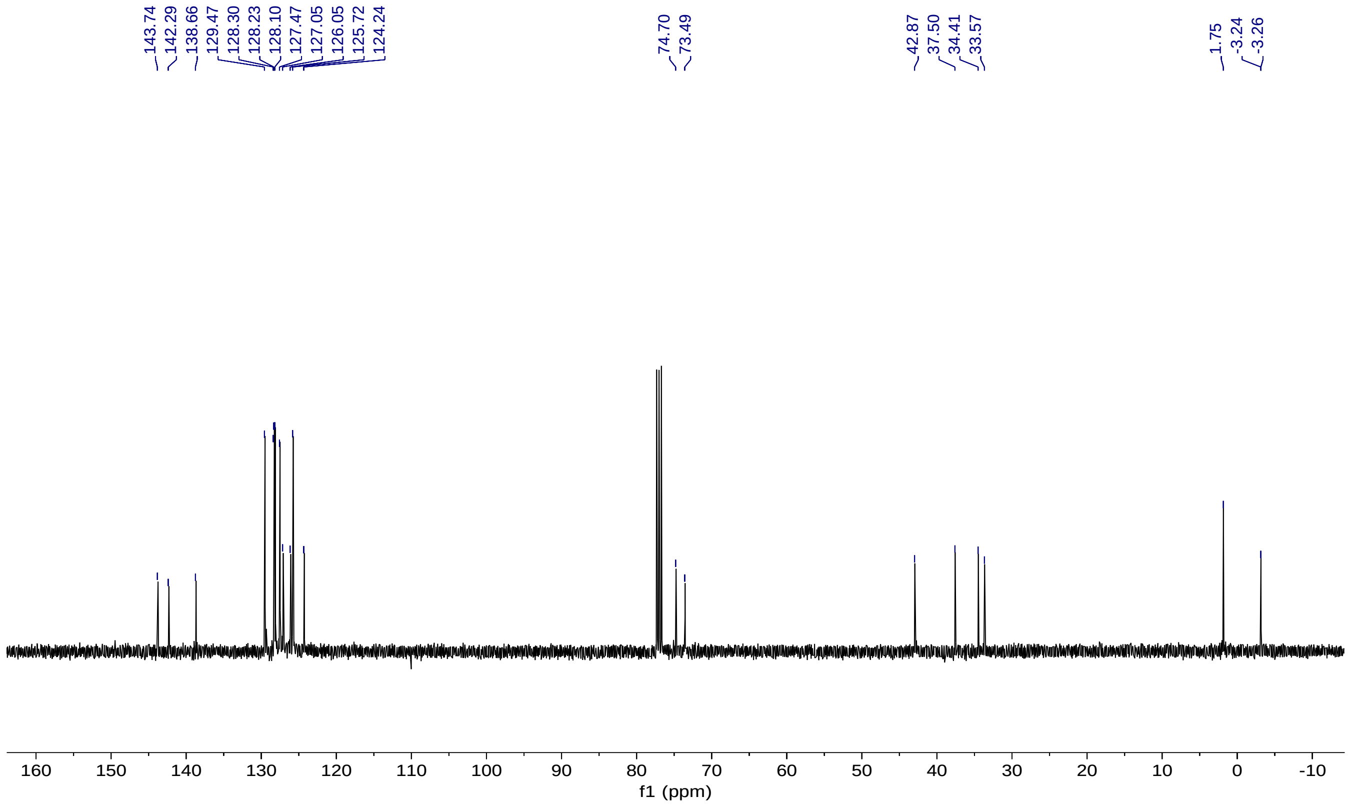

^{1}H NMR (500 MHz, CDCl$_3$)

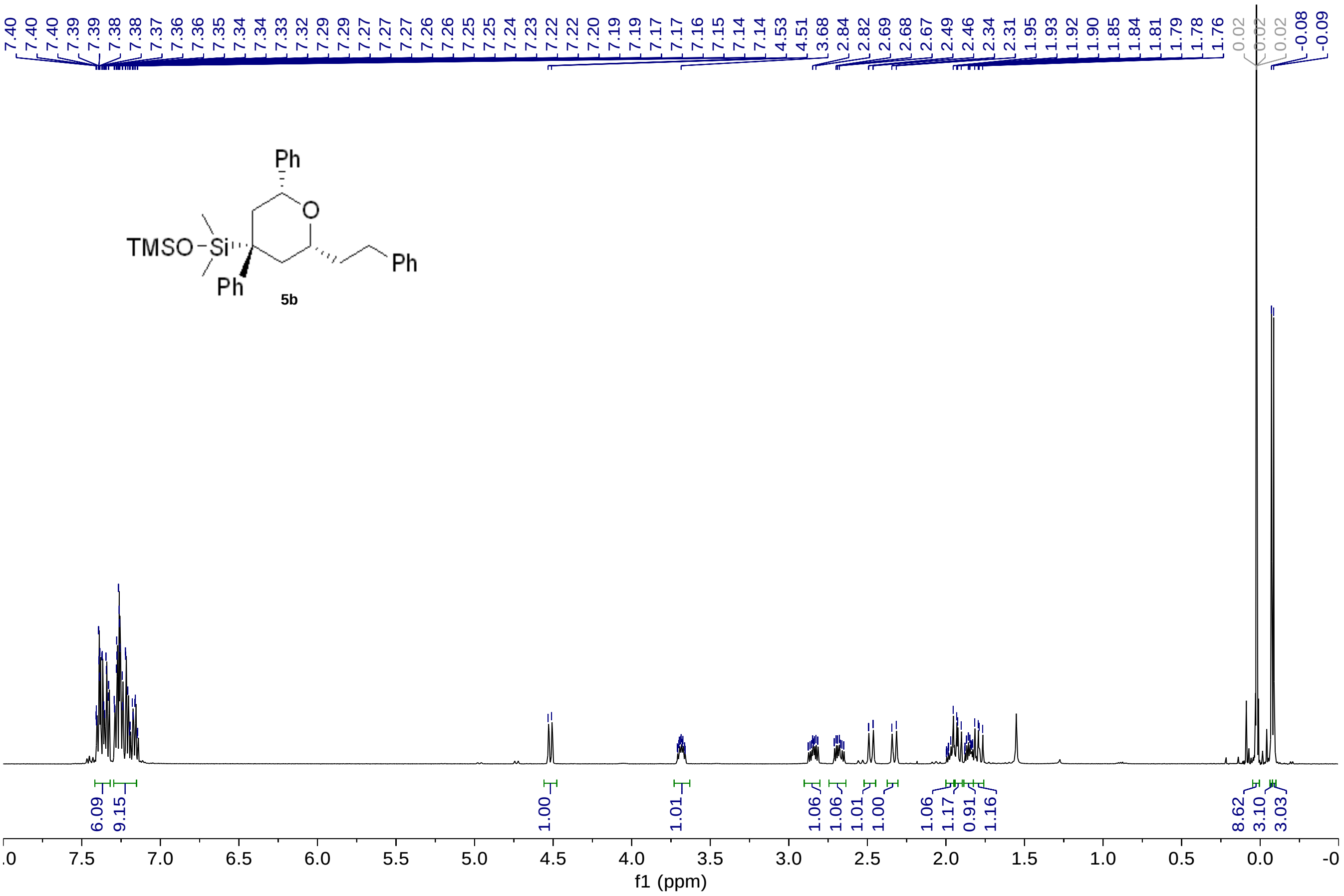

^{13}C NMR (126 MHz, CDCl$_3$)

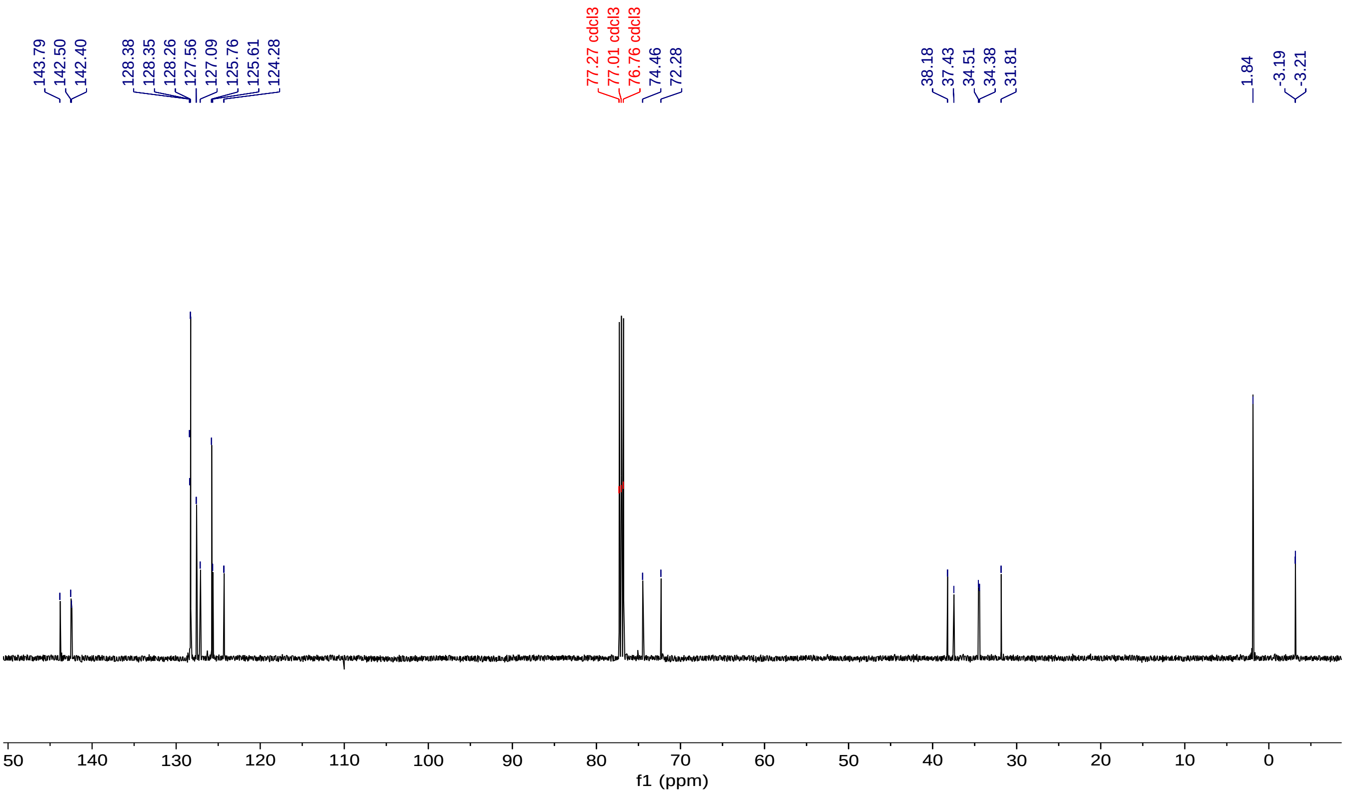

^{1}H NMR (400 MHz, $CDCl_3$)

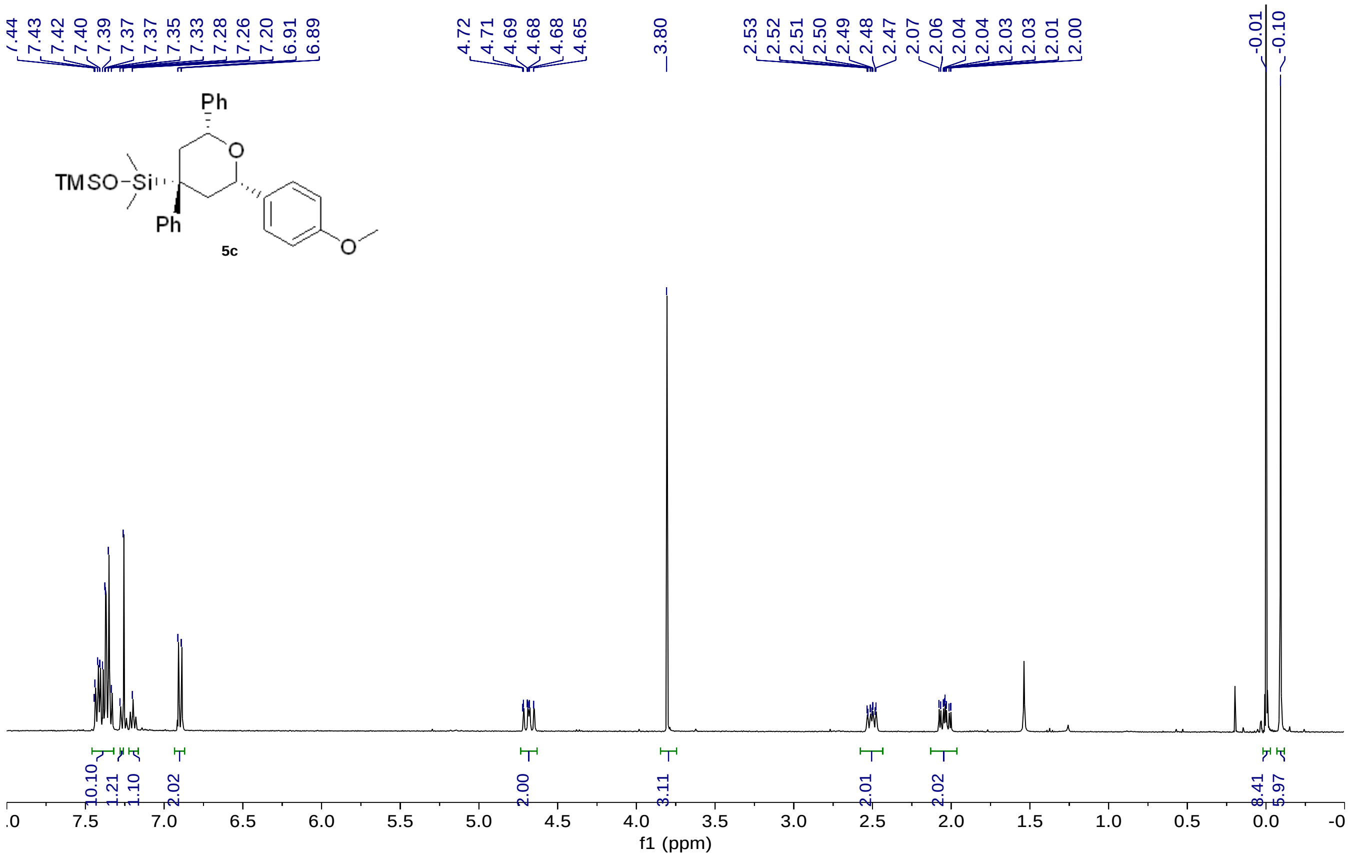

^{13}C NMR (101 MHz, $CDCl_3$)

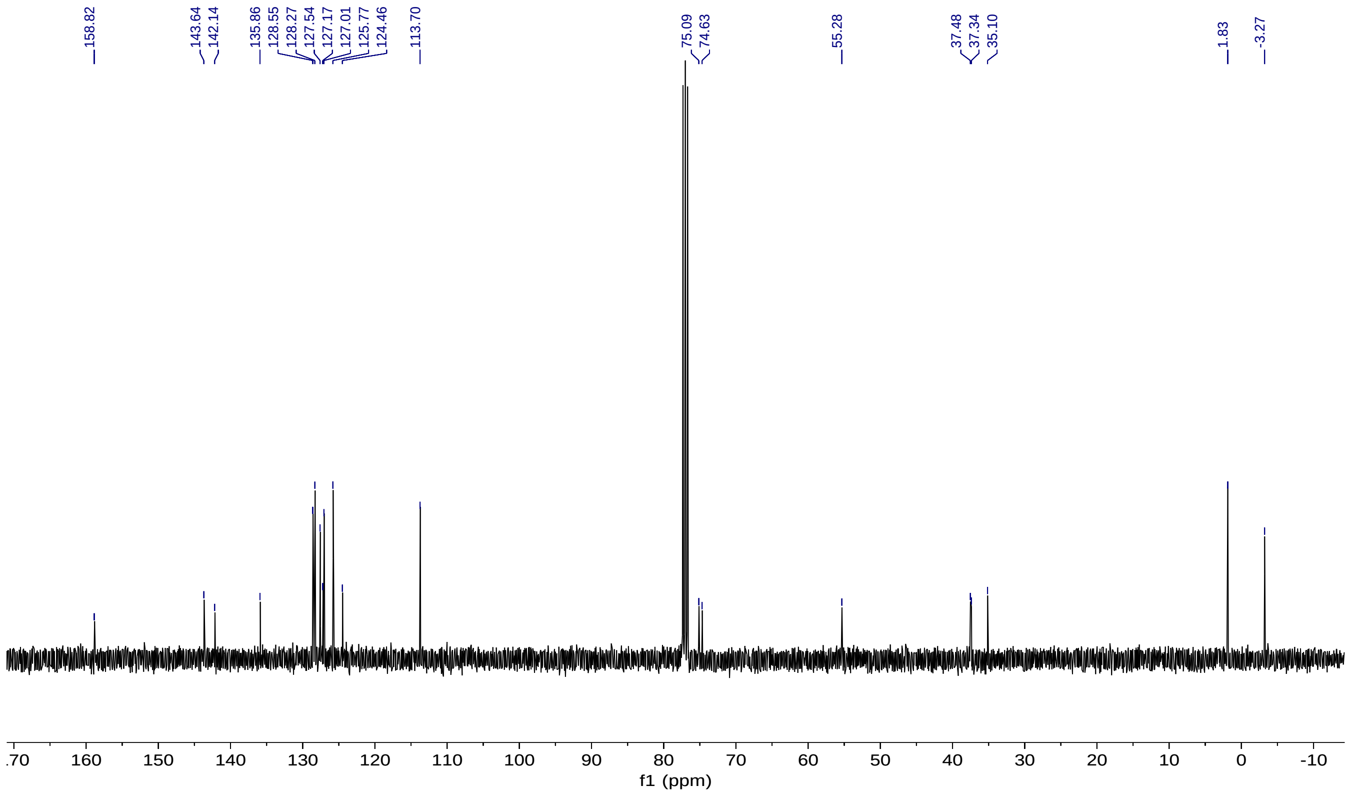

^{1}H NMR (400 MHz, CDCl$_3$)

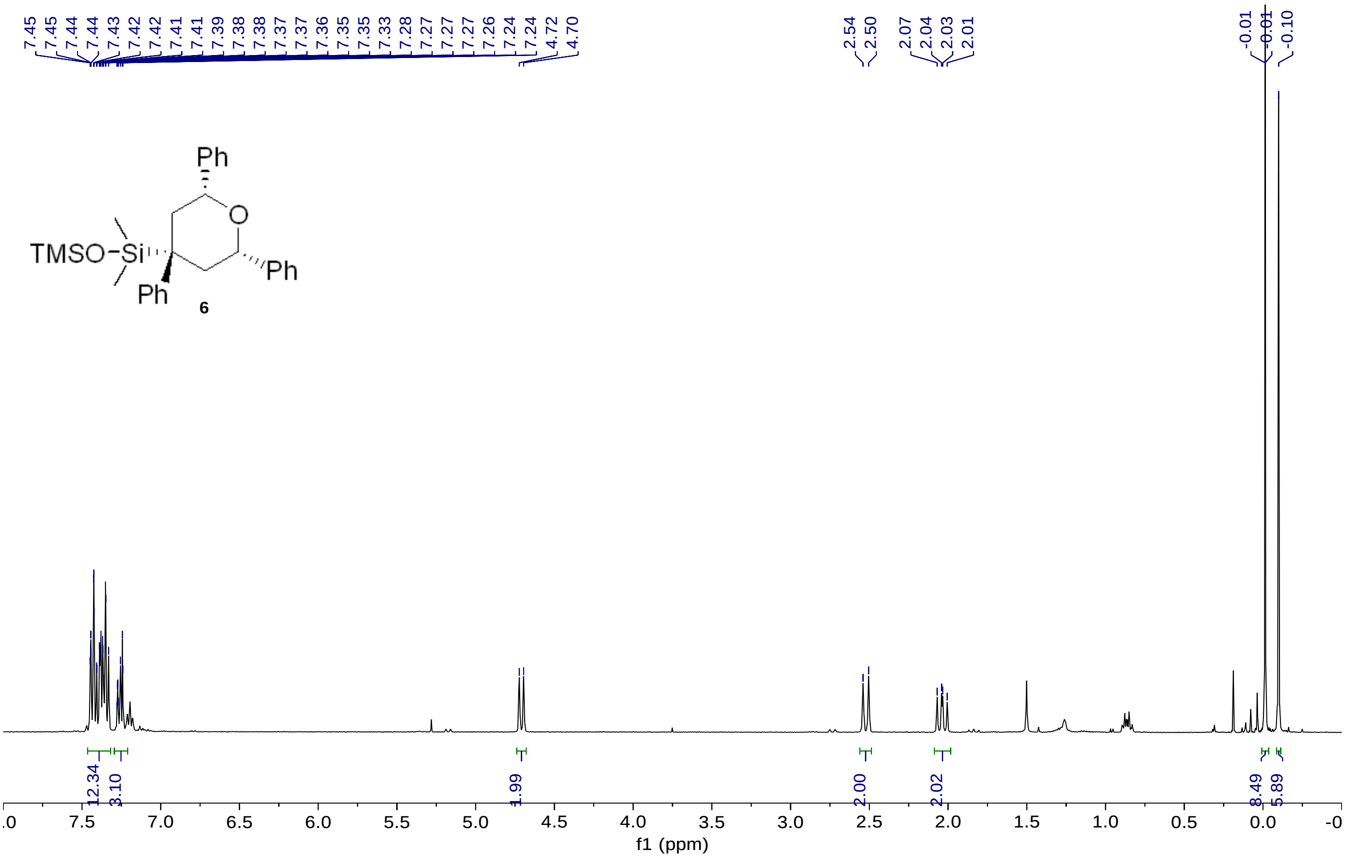

^{13}C NMR (101 MHz, CDCl$_3$)

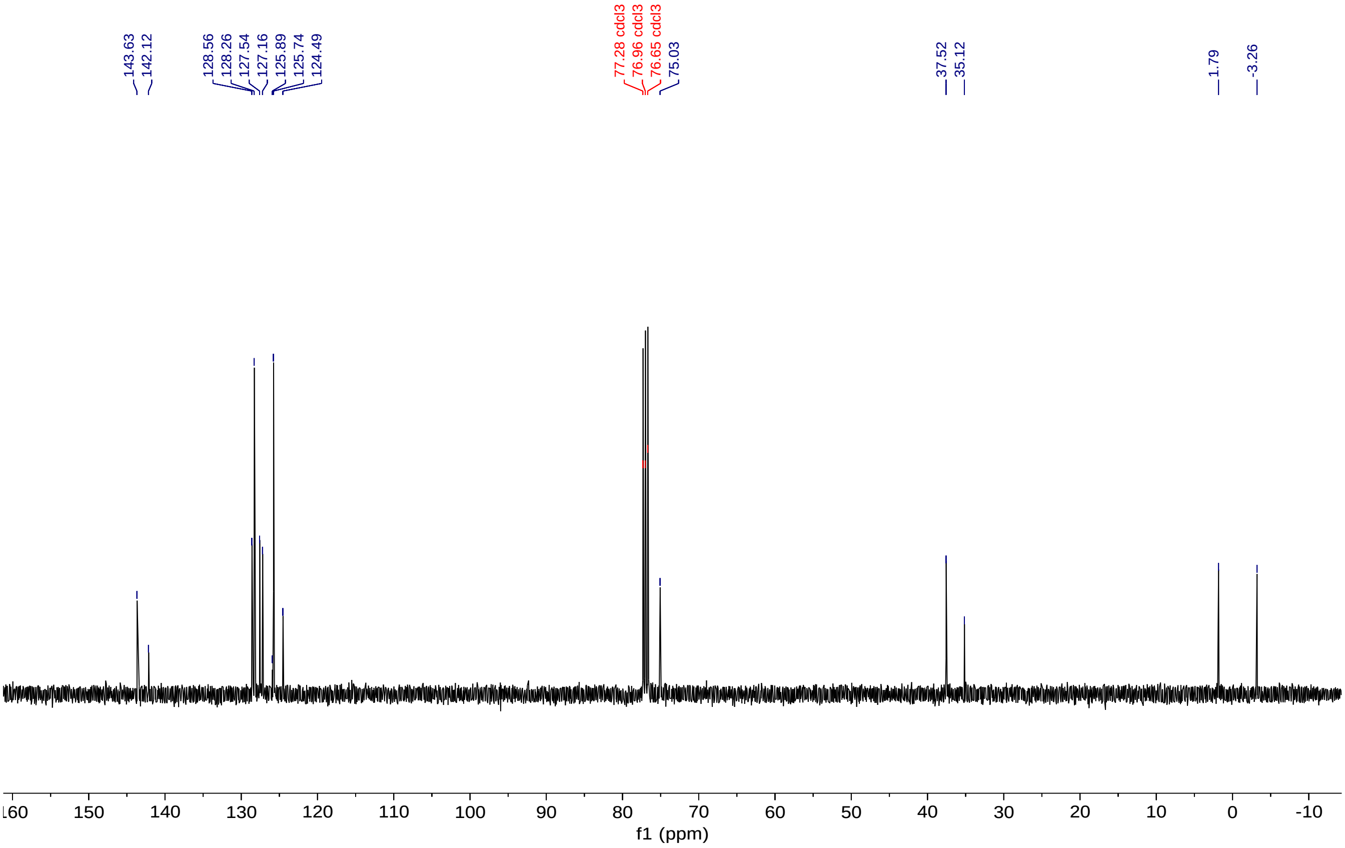

^{1}H NMR (500 MHz, $CDCl_3$)

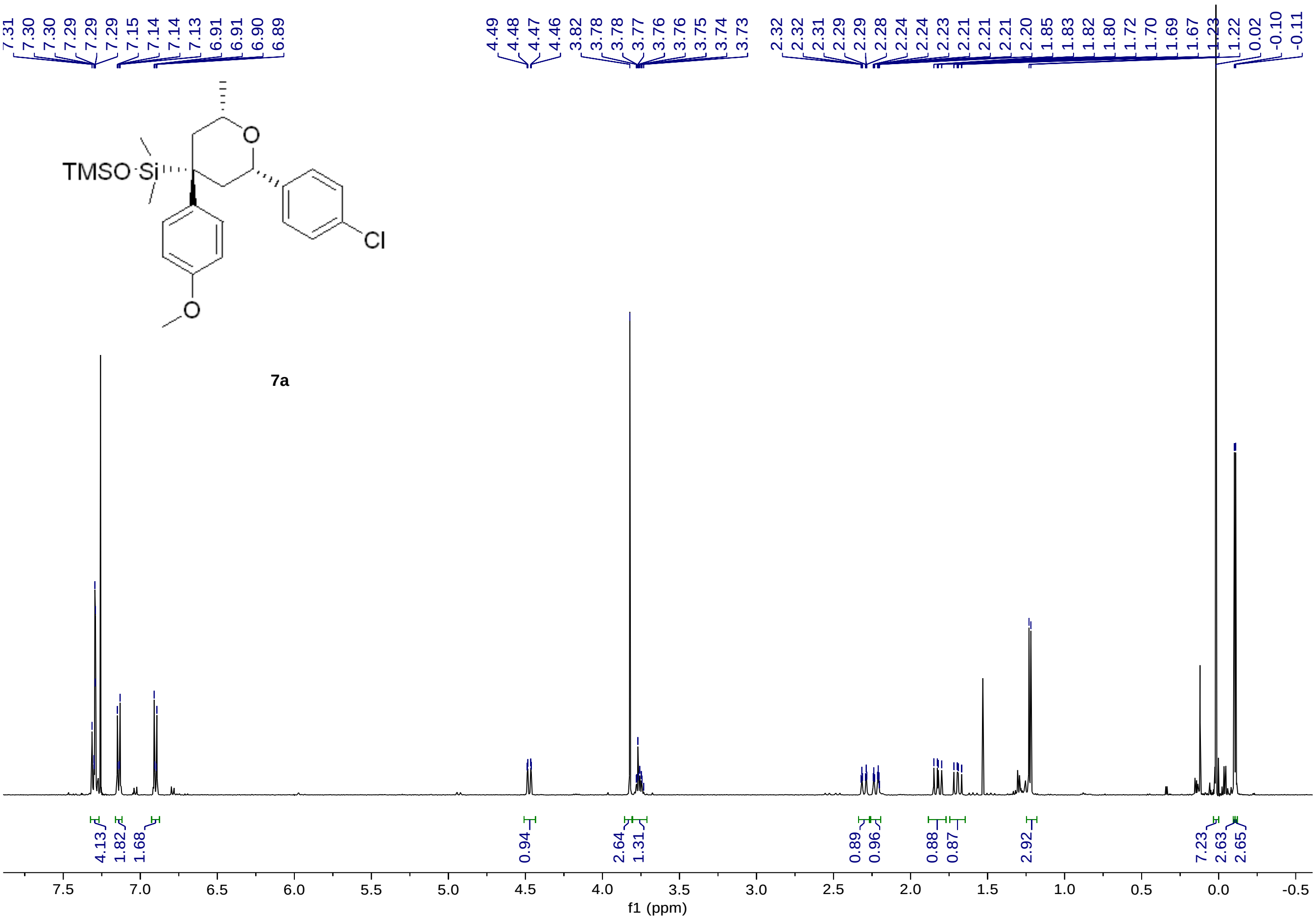

^{13}C NMR (101 MHz, $CDCl_3$)

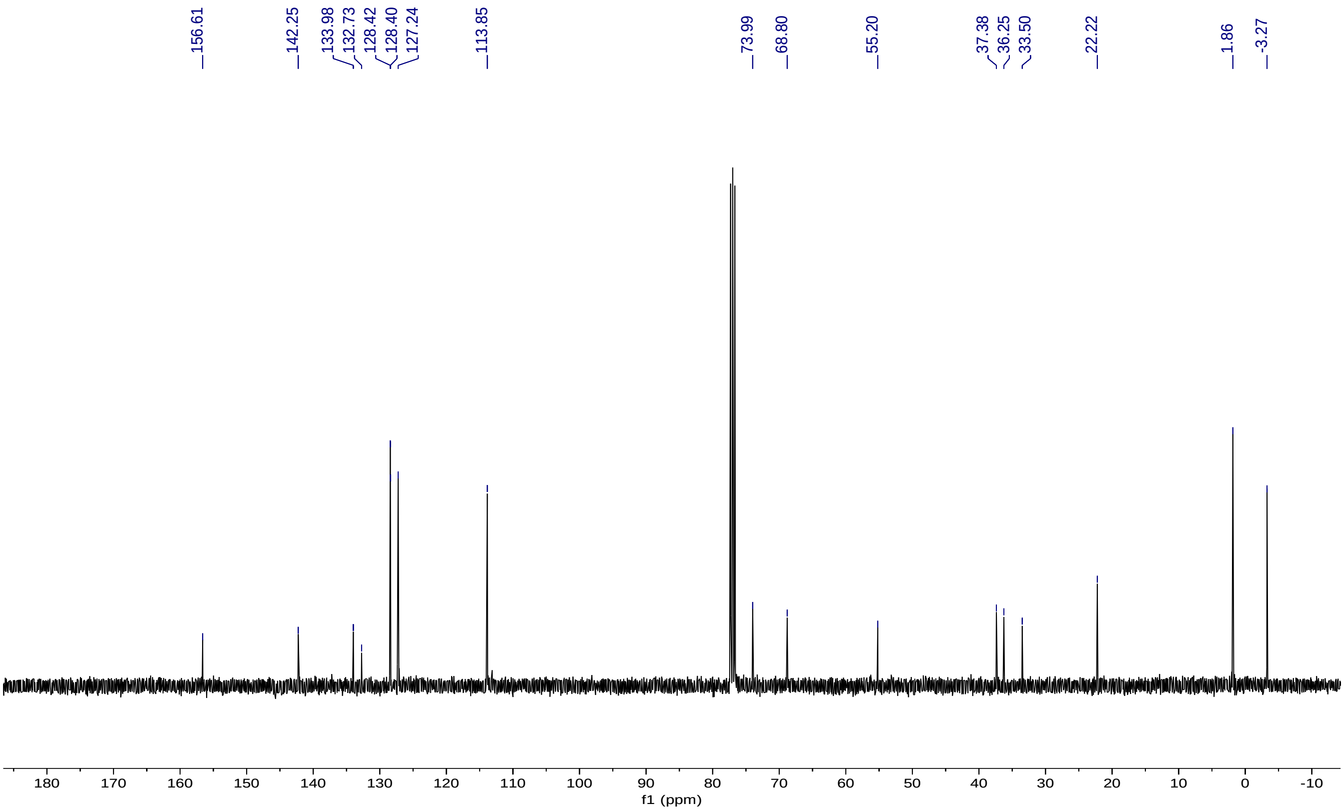

^{1}H NMR (500 MHz, $CDCl_3$)

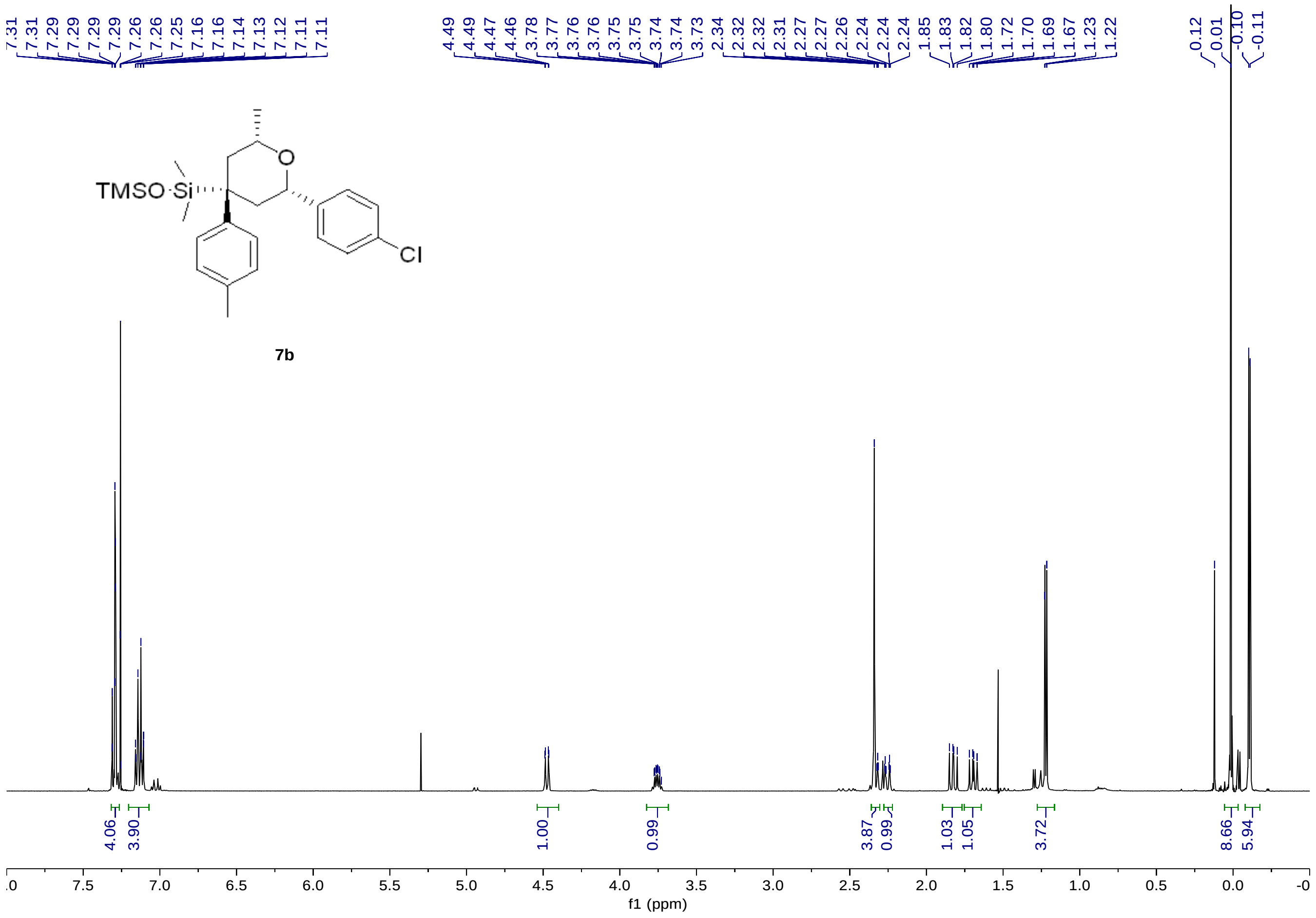

^{13}C NMR (101 MHz, $CDCl_3$)

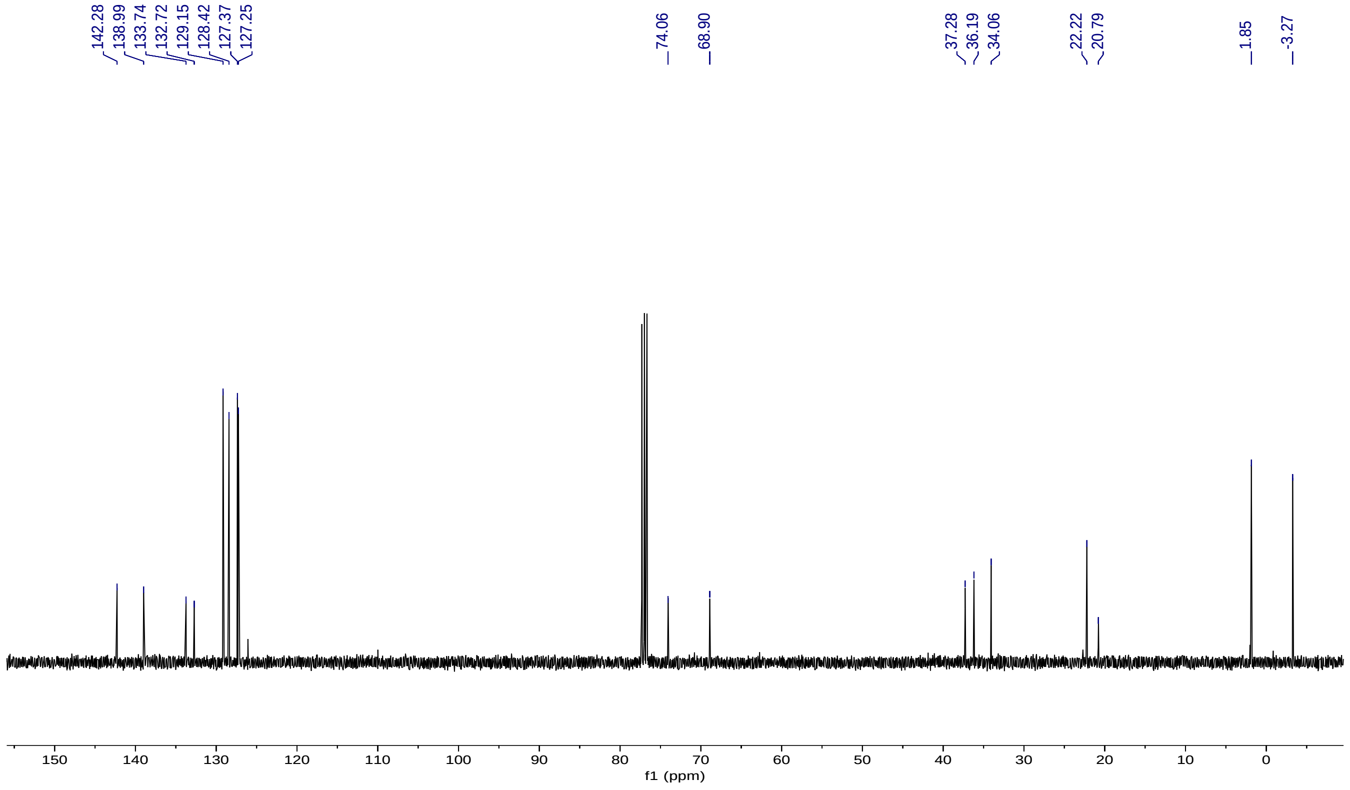

^{1}H NMR (500 MHz, $CDCl_3$)

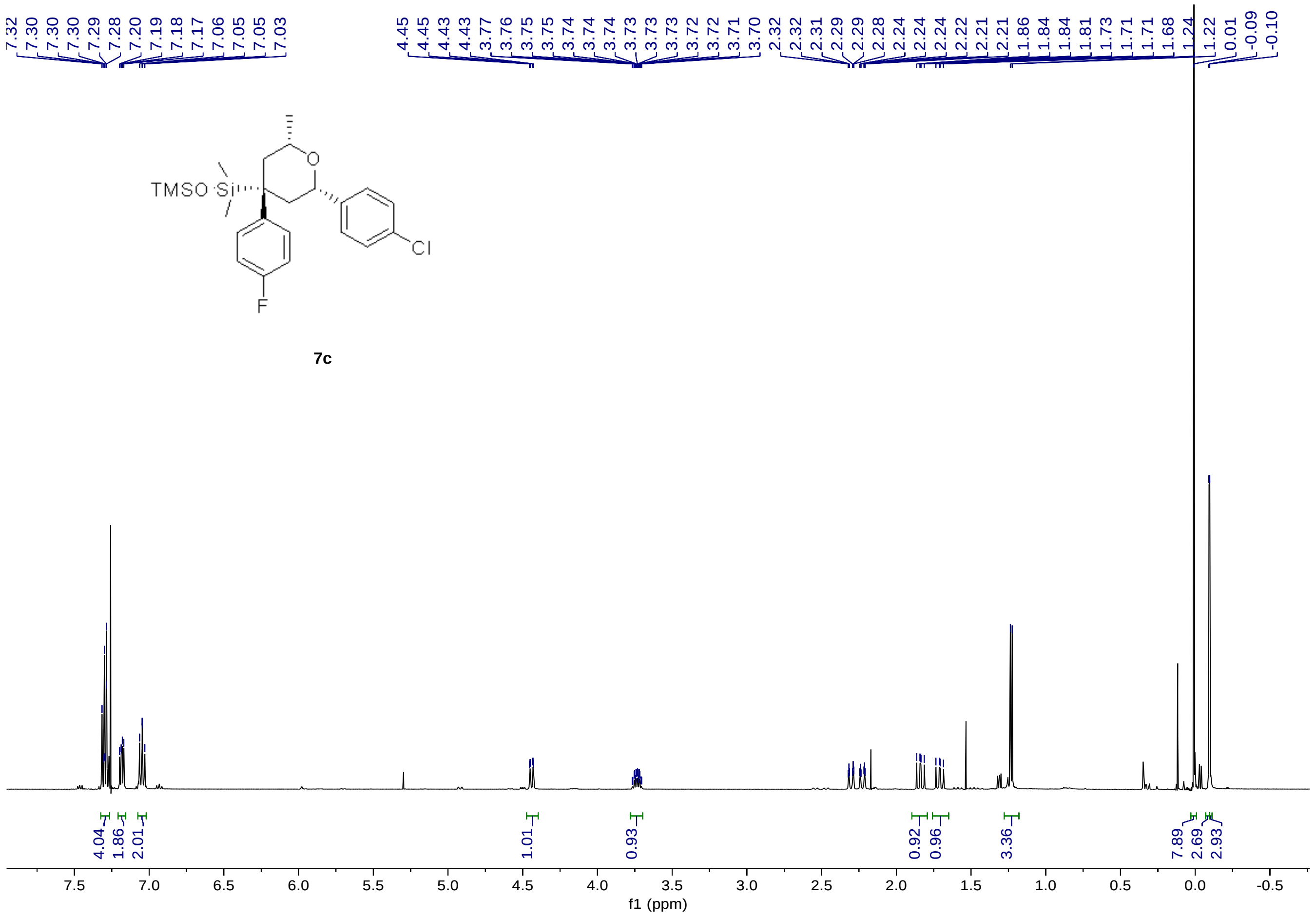

^{13}C NMR (101 MHz, $CDCl_3$)

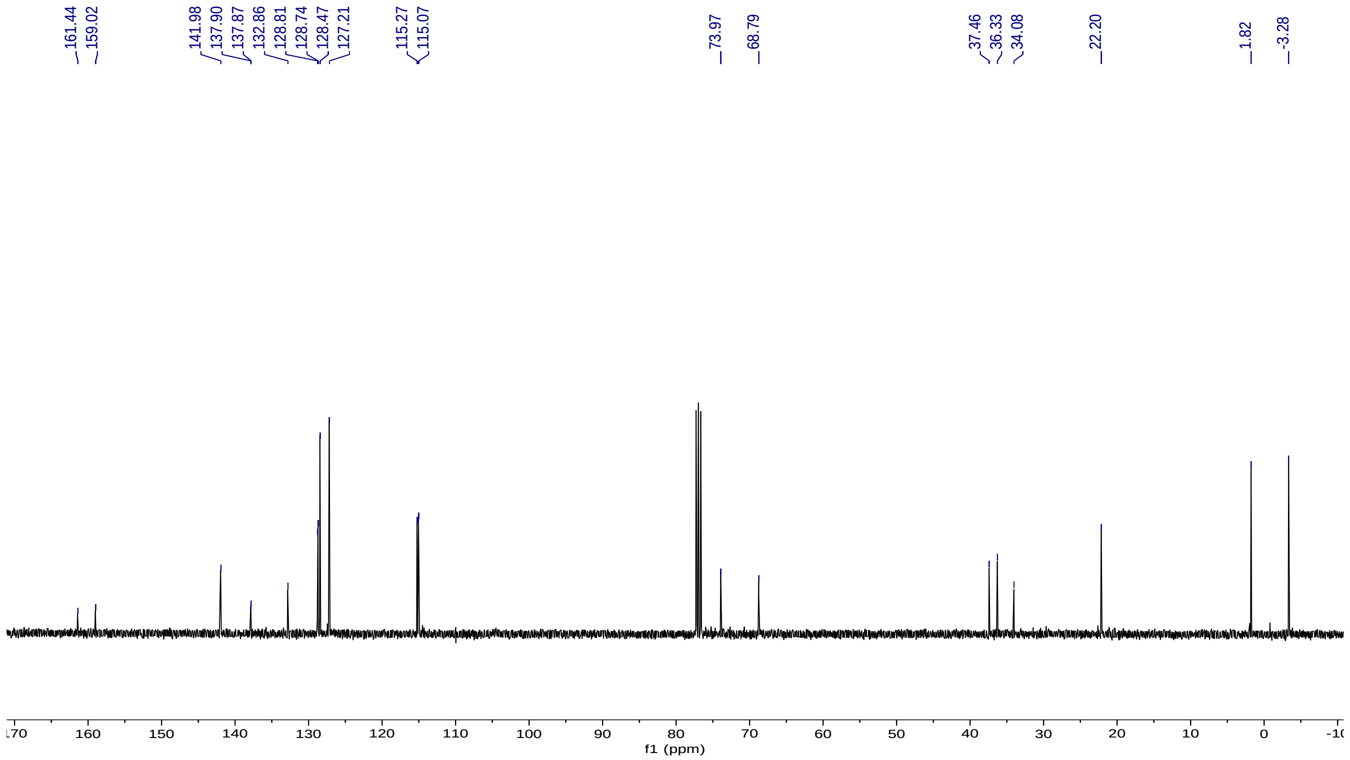

5. References

[1] Y. Su, Q.-F. Li, Y.-M. Zhao, P. Gu, *Org. Lett.* **2016**, *18*, 4356–4359.

[2] CrysAlisPro-Data Collection and Integration Software, Agilent Technologies UK Ltd, Oxford, UK, 2011.

[3] Dolomanov, O. V; Bourhis, L. J.; Gildea, R. J.; Howard, J. A. K.; Puschmann, H. OLEX2: A Complete Structure Solution, Refinement and Analysis Program. *J. Appl. Crystallogr.* **2009**, *42*, 339–341.

[4] Sheldrick, G. M. Crystal Structure Refinement with SHELXL. *Acta Crystallogr., Sect. C.* **2015**, 71, 3–8.

[5] Macrae, C. F.; Sovago, I.; Cottrell, S. J.; Galek, P. T. A.; McCabe, P.; Pidcock, E.; Platings, M.; Shields, G. P.; Stevens, J. S.; Towler, M.; Wood, P. A. Mercury 4.0: from visualization to analysis, design and prediction *J. Appl. Crystallogr.* **2020**, 53, 226-235.
